# A Comparison of Mechanisms Driving Lesion Outcomes During Lung Tumor and Tuberculosis Granuloma Formation

**DOI:** 10.1007/s11538-026-01763-8

**Published:** 2026-10-06

**Authors:** Christian T. Michael, Maral Budak, Denise Kirschner

**Affiliations:** Kirschner Lab, Department of Microbiology & Immunology, University of Michigan - Michigan Medicine, Ann Arbor, MI USA

**Keywords:** Small-cell lung cancer, agent-based models

## Abstract

Small cell lung cancer (SCLC) and tuberculosis (TB) are both deadly diseases that present with spatially complex lung lesions. These lesions share similarities, including spatial interactions between T cells and macrophages. Both SCLC and TB exhibit heterogeneous disease progression and responses to treatment; current experimental methods have few tools to investigate the spatiotemporal evolution of these lesions within human lungs. We have applied our computational agent-based model (ABM), *GranSim*, to extensively study heterogeneity of TB granuloma formation and treatment efficacy. We introduce *TumorSim*, an analogous ABM designed to understand heterogeneity of SCLC lung tumors. *TumorSim* mechanistically captures immune-tumor interactions, many of which are well-studied in isolation, including cytokine-based recruitment of immune cells and PD-1/PD-L1-based inhibition of cytotoxic T cells. Drawing from lung immunology literature, we define and explore wide parameter ranges to characterize *TumorSim* behavior using global sensitivity analysis. We compare factors that drive both tumor and granuloma outcomes. As model validation, sensitivity analysis captures several well-known correlates of improved SCLC outcomes including macrophage-mediated cytotoxic T-cell recruitment. Surprisingly, both models predict a two-phase formation process occurring with an abrupt change in tumor/granuloma dynamics upon arrival of adaptive immune cells into the lung. Simulations suggest that while C–C motif chemokine ligand 5 (CCL5) is associated with improved tumor control later during tumor growth, CCL5 plays a pro-tumor role early during tumor growth by recruiting regulatory T cells. We find that, like virtual granulomas, *TumorSim* tumors become enlarged when immunosuppressive mechanisms outweigh pro-inflammatory responses. This novel tumor model enables future studies of both immunotherapeutics and anti-cancer drugs.

## Introduction

Small cell lung cancer (SCLC) and pulmonary Tuberculosis (TB), two leading causes of death worldwide, both manifest in lungs with lesions that share anatomic and functional similarities that directly impact disease progression and responses to therapies. SCLC, an aggressive form of lung cancer with a  < 7% five-year survival, accounts for about 15% of lung cancer cases (Zhong et al., 2026). TB is a bacterial disease caused by infection with *Mycobacterium tuberculosis* (Mtb), and is the world’s leading cause of death by a single infectious pathogen and has been for decades (World Health Organization 2025). Discrete lung masses form in and on lung stroma during both SCLC and pulmonary Mtb infection, and in each disease these masses result from recruitment of multiple, overlapping immune cells to both lesions and their ensuing complex interactions. In fact, due to the similarities between TB and SCLC, TB patients are often misdiagnosed with lung cancer based on PET-CT scans in regions with high TB incidence (Low et al. 2006; Werutsky et al. 2019). Moreover, many studies have provided evidence that patients with TB have an increased risk of lung cancer (Wu et al. 2011; Yu et al. 2011; Ho et al. 2021; Fang et al. 2025). Treatment of both SCLC and TB presents formidable challenges necessitating multi-drug regimens designed to overcome heterogeneous spatial drug delivery and pre-existing and/or acquired drug resistance. While effective (though intensive and expensive) treatment for TB exists (Saukkonen et al. 2025), current therapies for SCLC fail for many patients (Li et al. 2022; Kim et al. 2025), emphasizing the need for us to better understand basic immunological mechanisms and drivers of tumor heterogeneity. A mechanistic computational model can help identify these factors.

**Fig. 1:**
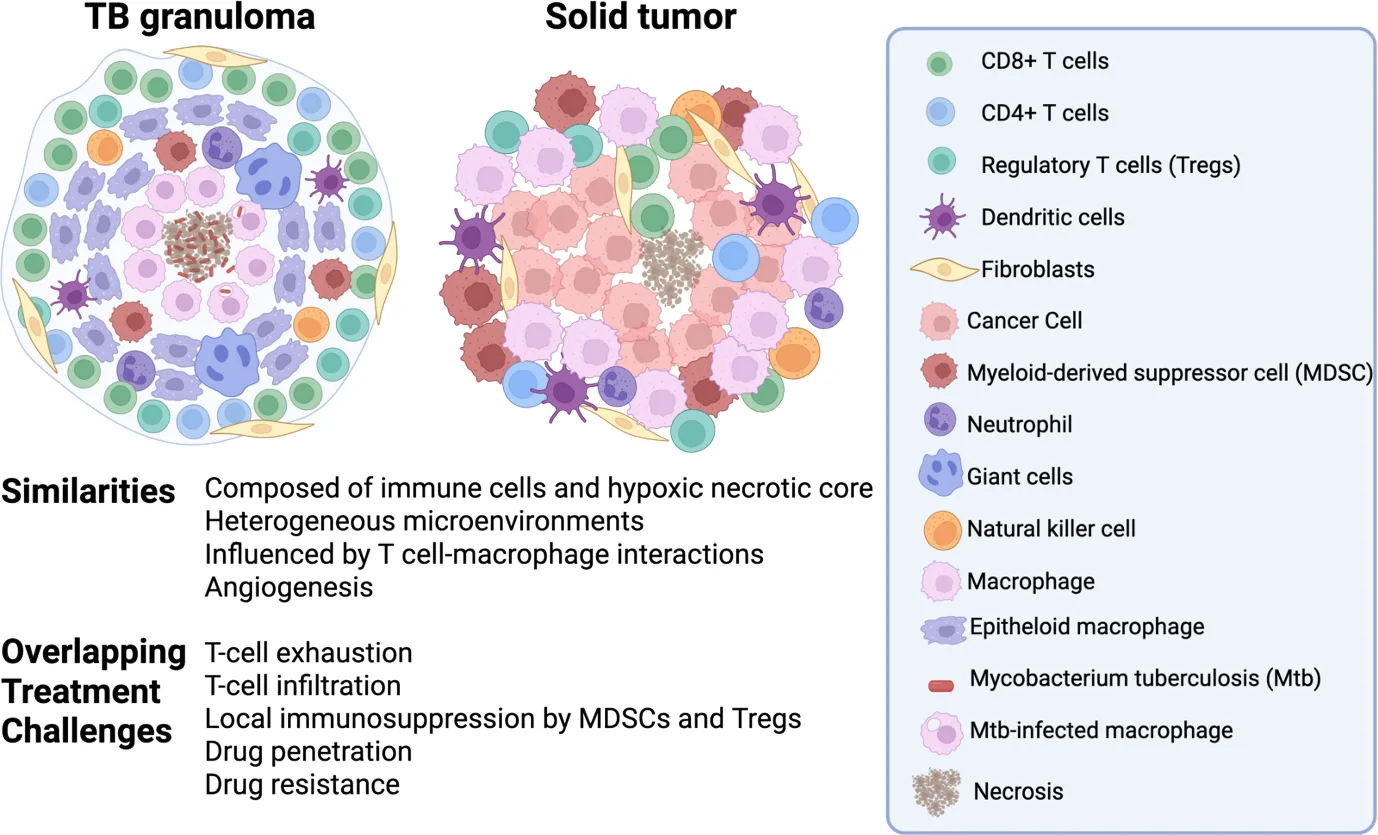
Summary comparison of tuberculosis (TB) granuloma and solid tumor lesion microenvironments. These cartoons depict the typical presentations of SCLC and TB. Granulomas are approximately spherical (Ramakrishnan 2012), while SCLC tumors tend to be lobular once diagnosed (Travis 2012), likely shaped by the stromal architecture. We thus assume that for small, early tumors, a sphere is a reasonable approximation. Both lesions comprise multiple types of immune cells clustering around Mtb infection and neoplasm, respectively. Initial interaction between macrophages and dendritic cells results in the recruitment of an adaptive immune response. Activity of apoptosis-inducing leukocytes (cytotoxic T cells, natural killer cells, pro-inflammatory macrophages) is critical for effective resolution and is hampered by anti-inflammatory leukocytes such as regulatory T cells, anti-inflammatory macrophages, and myeloid-derived suppressor cells (Li et al. 2022; Fang et al. 2025). The core of each lesion is often hypoxic and necrotic (Ramakrishnan 2012; Li et al. 2022). Created in BioRender. Michael, C. (2026) https://BioRender.com/a4s97gv

Lung cancer, especially SCLC, has many features in common with TB. TB granulomas and lung cancer lesions pose remarkably similar challenges to disease elimination via immune response or treatment (Fig. 1). In both, an effective immune response is impaired in the lung microenvironment due to increased immunosuppressive cells (myeloid-derived suppressor cells or regulatory T cells) or T-cell exhaustion (Whiteside 2008; Wang et al. 2019; Ashenafi et al. 2022). Spatially-heterogeneous architectures of granulomas and tumors, including poorly developed vasculature and distorted extracellular matrix, impair drug delivery and T-cell infiltration into both types of lesions (Sarathy et al. 2016; Peranzoni et al. 2018; Millar et al. 2020). The resulting heterogeneous distribution of drugs throughout lesions contributes towards the commonly-observed outcomes of drug resistance and subsequent treatment failure, especially in combination with pre-existing or acquired mutations in granulomas/tumors due to nutrient-poor lesion microenvironments (Ormerod 2005; Wu et al. 2014). These challenges necessitate extended courses of treatments for both diseases with multiple drugs (Bayat Mokhtari et al. 2017; Kerantzas et al. 2017).

TB lung granulomas and lung tumors share anatomic and functional similarities that directly impact disease progression and response to therapy (Fang et al. 2025). In response to infection with Mtb or formation of cancer cells, the host recruits a wide variety of immune cells, including effector and immunosuppressive myeloid lineage cells and T lymphocytes, and other stromal cell types such as activated fibroblasts. This results in the emergence of complex and spatially heterogeneous structures called granulomas (TB) or solid tumors (cancer) (Fig. 1) (Bickett et al. 2020; Dorhoi et al. 2020; Ashenafi et al. 2022; Lasser et al. 2024). Host immune responses commonly do not eliminate Mtb or cancer cells, producing two outcomes: (1) progressive disease in either case; or (2) clinical latency (TB) or dormancy (cancer), in which a small number of bacteria or cancer cells remain in a viable, growth-arrested or slowly-dividing state for extended periods of time (Lin et al. 2018; Fabrizio 2025). The course of TB and lung cancer is heavily dependent on the interactions between T lymphocytes and macrophages in lesion microenvironments (Bickett et al. 2020; Ashenafi et al. 2022).

Despite the many cell-scale similarities between SCLC and TB, the two diseases bear several important differences. At its core, cancer is a disease caused by a dysregulation of a host’s own cells, thus tumor cells are immune-evasive (i.e., poorly targeted by the immune system), and several recent cancer therapies are aimed at engaging the immune system via exogenous means (Qi et al. 2024; Lowery et al. 2025). When left untreated, SCLC frequently results in large lesions with a very high mortality rate (Wang et al. 2022). By contrast, the human adaptive immune system is relatively efficient at identifying the presence of TB, yet Mtb itself has evolved to inhibit macrophages’ ability to digest the bacterium (Ramakrishnan 2012). Consequently, many macrophages respond to Mtb infection, but the immune system struggles to sterilize infection even once it has been engaged unless the system is activated; the time delay for activation usually means the immune system lags behind bacterial growth, reducing efficacy. Common treatments for TB are combinations of bacteriostatic and bactericidal antibiotics (Bonnett et al. 2017; Cicchese et al. 2020a, b). These and other differences between the two lesions contribute to distinct characteristic time-scales of SCLC and TB, despite the many overlapping cell types and immune mechanisms. SCLC tumors expand quickly largely unchallenged by the immune system, while non-sterile granulomas may linger for years as sites of chronic inflammation (Lin et al. 2014, 2018). Finally, differences in characteristic timescales means that there are no temporally direct comparisons for identifying driving mechanisms of outcomes.

Emergent lung lesions such as tumors and granulomas are challenging to study, as gross predictive power can be elusive despite knowing the myriad of constituent mechanisms and key cell types present. An effective method for identifying principle drivers of clinical outcomes are in silico, mechanistic, multivariate, mathematical models. When calibrated and validated to experimental and clinical datasets, multi-scale models are useful and cost-efficient at capturing complexities of a biological system that may be challenging to probe by experimental methods. We have previously developed a multi-scale mechanistic hybrid agent-based model, *GranSim*, that recapitulates heterogeneous outcomes of lung granuloma progressions, entirely emergent from cell-scale immune responses to pulmonary Mtb infection (Segovia-Juarez et al. 2004; Ray et al. 2009; Fallahi-Sichani et al. 2011; Cilfone et al. 2013). In this work, we create a novel, tandem hybrid agent-based model to understand factors that contribute to lung solid tumor formation and heterogeneity. We introduce a new multi-scale model, *TumorSim*, that captures mechanisms driving SCLC lung tumor formation dynamics. We compare and contrast *TumorSim* to the dynamics of our well-characterized *GranSim* model behavior to investigate similarities and differences between mechanisms of TB and SCLC. Our goal is to harness cross-talk between these two disparate disciplines (infection disease and cancer) that will help foster new ideas and research advancement for both.

## Methods

To study emergent lesion structures that arise such as SCLC tumors or TB granulomas, we need to explicitly represent cell-and molecular scale details. Several simplified models of tumors exist, though these models focus on large-scale measurements and embed mechanistic hypotheses within the structure of their governing equations (Mohsin et al. 2024). We choose to model both the temporal and spatial aspects of SCLC lung tumors that have been identified as key drivers of lung disease treatment outcomes (Dangaj et al. 2019; Wu et al. 2019; Zheng et al. 2021; Kohli et al. 2022; Li et al. 2022). Agent-based models (ABMs) represent biological systems as collections of interacting *agents* (e.g., cells) that interact to form emergent structures; direct representation allows for mechanistic probing of how smaller-scale agents affect larger-scale outcomes (e.g., tumors and granulomas). ABMs allow for explicit representation of cell-scale mechanism and modular updates as new information becomes available and have been used for a variety of applications (Cockrell et al. 2017; Ghaffarizadeh et al. 2018; Liu et al. 2021).

Several general ABM frameworks have been developed to generate cell-scale simulations; using these a modeler may follow a straightforward protocol to create a cancer simulation (Poleszczuk et al. 2016). Chaste is a C++ library that provides a platform for rapid and reproducible prototyping of cancer, heart, and soft tissues using center-based, vertex-based, or Cellular-Potts-based architectures (Mirams et al. 2013). Chaste was recently used to study macrophage interactions within the tumor microenvironment (Bull et al. 2023). PhysiCell provides a substantial ecosystem of ABM tools that allow modelers to define rules in human-readable, controlled-vocabulary terms and has been used to simulate a wide variety of biological systems (Ghaffarizadeh et al. 2018). PhysiCell was used to build a high-throughput environment for testing hypotheses in solid tumors (Ozik et al. 2018). CompuCell3D is a comprehensive modeling platform built by extending the Cellular Potts framework and is capable of capturing complex cell morphologies (Swat et al. 2012). The Hybrid Automata Library (HAL) is a mathematical oncology framework that focuses on layering ABMs with ODEs and PDEs (Bravo et al. 2020). Morpheus provides a powerful and flexible model framework (Starruss et al. 2014), and typically represents cell-based ABMs as either center- or vertex-based. There are many other ready-made frameworks with various advantages and disadvantages, many of which were neatly outlined by the HAL developers (Bravo et al. 2020).

Published cancer and granuloma models have investigated multiple aspects of cancer and TB progression (Metzcar et al. 2019; Norton et al. 2019). These models address important questions around immune dynamics (Castiglione et al. 2005; Lollini et al. 2006; Pennisi et al. 2010; Palazon et al. 2011; Pappalardo et al. 2011; Woelke et al. 2011; Kim et al. 2012; von Eichborn et al. 2013; Pourhasanzade et al. 2017), growth of metastases (Enderling et al. 2009a, b; Biava et al. 2011; Enderling et al. 2012; Wynn et al. 2012), drug resistance (Silva et al. 2010; Powathil et al. 2012; Wang et al. 2015), tumor immunotherapy, tumor-enhancing immune/stromal cells, tumor heterogeneity (Norton et al. 2014), angiogenesis, and more (Poleszczuk et al. 2016; Metzcar et al. 2019; Norton et al. 2019). Gong et al. (2017) developed a multiscale, three-dimensional (3D) ABM of interactions between immune and cancer cells, which revealed emergent global behaviors during tumor development and therapy. Menezes et al. and Calopiz et al. created ABMs detailing the pharmacokinetics of antibody drug conjugate treatment of solid tumors, relating drug penetration to overall impact on treatment efficacy for established tumors (Menezes et al. 2022; Calopiz et al. 2024). A recent study presented a detailed model of nutrient transport through avascular tumors and its impact on tumor growth (Almudarra et al. 2024). Similarly, ABMs have been used to study TB. Warrender et al. used an ABM framework to investigate early Mtb infection (Warrender et al. 2006). More recently, 3-D ABMs have been used to simulate in vitro granuloma models, with a focus on T-cell/Macrophage interactions on granuloma outcomes (Petrucciani et al. 2024). A larger-scale ABM, the “bubble model”, was used to study how multiple confluent TB granulomas merge within lungs (Prats et al. 2016).

We have chosen to build on *GranSim*, the C++-based ABM framework that we have created and curated for over 20 years for capturing TB granulomas. We have previously used *GranSim* to study immune and bacterial dynamics during infection (Cardona et al. 2001, 2005; Marino et al. 2004; Segovia-Juarez et al. 2004; Gammack et al. 2005; Ray et al. 2009), antibiotic treatment (Cilfone et al. 2015a, b; Pienaar et al. 2015a, b; Cicchese et al. 2020a, b; Budak et al. 2023, 2024), perform virtual clinical trials to predict optimal treatment strategies (Marino et al. 2007, 2014; Fallahi-Sichani et al. 2012; Cilfone et al. 2015a, b; Pienaar et al. 2015a, b; Cicchese et al. 2020a, b; Budak et al. 2023, 2024), also incorporating drug resistance (Pienaar et al. 2018). As SCLC tumors share many fundamental components, we create *TumorSim* on our foundational *GranSim* computational architecture platform. Here, we create novel agents, rules and features (Hypoxia inducible factor 1-α (HIF1α), oxygen, interferon gamma (IFN-ɣ), cancer cells etc.) and use wide ranges of parameters to explore a range of tumor model outputs. *TumorSim* reproduces temporal dynamics of cytotoxic T cells and cancer cells during tumor progression, as well as spatial distributions of these cells. A comparison and contrast between mechanisms included in *GranSim* and *TumorSim* is shown in Table 1 showing both overlap and distinction between features.

**Table 1:** Comparison and contrast between *GranSim* and *TumorSim* mechanisms

	*GranSim*	*TumorSim*
Initialization	- Resting macrophages and vascular sources randomly distributed on simulation grid - One macrophage infected with one mycobacterium at the center of the simulation grid	- Vascular sources randomly distributed on simulation grid - A circular neoplasm of ten cancer cell diameter at the center of the simulation grid - Few, and M1 macrophages at the center of the simulation grid
Macrophages	- Present as resting (Mr), activated (Ma), infected (Mi), chronically infected (Mci) - Ma kills Mtb effectively - Mi provides a permissive niche for Mtb to grow - Mci cannot kill Mtb and bursts	- Present as M1 and M2 - M1 kills cancer cells - M2 deactivates CTL - IFN-ɣ polarizes M2 toward M1 phenotype - HIF1α polarizes M1 toward M2 phenotype
T cells	- Present as cytotoxic (CTL), IFN-ɣ -secreting (Tgam) and regulatory (Treg) - CTL and Tgam are proinflammatory and induce apoptosis in Mi and Mci - Treg is anti-inflammatory and downregulate CTL and Tgam	- Present as Treg and CTL - CTL is proinflammatory and kills cancer cells, can be PD-1+ or PD-1- - Treg is anti-inflammatory and deactivates CTLs
Vascular blockage	- Vascular sources blocked depending on the amount of necrotization and cell density	- Dynamic vascular permeability based on the level of hypoxia
Chemokine-cytokine secretion	- Chemokines: CCL2, CCL5 and CXCL9 secreted by Mi, Ma and Mci - Cytokines: TNF-α secreted by Tgam and CTL; IFN-ɣ secreted by Tgam; IL-10 secreted by macrophages, Tregs, and nominally by CTLs	- Chemokines: CCL2, CCL5 secreted by cancer cells, and CXCL9 M1 macrophages - Cytokines: IFN-ɣ secreted by M1 and CTL; - HIF1α implicitly secreted by all cells in low-oxygen environments
Recruitment	- Macrophages and T cells recruited from vascular sources based on the levels of CCL2, CCL5, CXCL9, and TNF-α	- Macrophages and T cells recruited from vascular sources based on the levels of CCL2, CCL5, CXCL9
Proliferation	- Bacteria proliferate with different rates based on the bacterial subpopulation - Immune cells do not proliferate	- Cancer cells proliferate - Immune cells may proliferate
Age-related cell death	Immune cells only	No age-related cell death

ABMs have the flexibility to be defined in two or three dimensions, and each choice has advantages and disadvantages. We have previously shown that with appropriate scaling, two-dimensional models accurately capture many aspects of lung lesion development if we assume radial symmetry. Accuracy captures concentration distributions (with scaling between 2-D and 3-D datasets if needed (Renardy et al. 2019)), cell counts, and principle mechanisms that are driving of gross outcomes (Marino et al. 2018; Linderman et al. 2010). We found that model predictions that were captured substantially differently between 2-D and 3-D models were the times it takes for specific cognate-conjugate cell pairs to meet and we showed this in a number of different ABM model formulations (Marino et al. 2018). Other models have used a quantitative systems pharmacology module to determine cancer cell proliferation and spatially homogeneous chemokine concentrations in a 3-D ABM for spatial analysis, but at a high computational cost (Ruiz-Martinez et al. 2022). Dimensional considerations must be made based on the purpose of the model under study. We use 2-D frameworks for both *TumorSim* and *GranSim* because: (1) our goal is to determine principal driving mechanisms for gross outcomes and sensitivity analysis requires hundreds of simulations; (2) 3-D ABM computational cost >2 orders of magnitude larger than 2-D; (3) we do not focus on spatially-dependent specific-cell-interaction events like antigen presentation within lymph nodes; and finally (4) most datasets for both granulomas and tumors are taken from 2-D data (e.g., slices of tissues, staining of tissues, etc.). In our previous work, we have shown that scaling immune ABMs from 2-D to 3-D faithfully captures many aspects of the human immune response that they do not track co-localization of specific cell pairs or detailed cell movement/chemotaxis on the grid (Marino et al. 2018; Renardy et al. 2019).

### *TumorSim*

To simulate emergence of a SCLC tumor, we create *TumorSim*, which captures key cells known to drive basic SCLC immunobiology. We build *TumorSim* as a multiscale, agent-based model that simulates interactions between immune cells, relevant molecules and cancer leading to formation of SCLC tumors on a 2-D simulation grid representing 6 mm x 6 mm of lung tissue. In this section, we describe key details of our development of *TumorSim*; full details of our model implementation can be found in our Overview, Design concepts, and Details-formatted model description document (Grimm et al. 2020) (Appendix A).

*TumorSim* contains two mathematically distinct types of agents. First are discrete agents that move or act based on rules (e.g., cell–cell interactions) at a slow timescale (ΔT=10 minutes). Discrete agents include vascular sources that serve as a source of cellular recruitment and O_2_ levels. All other discrete agents are cellular and include cancer cells that may or may not express the PD-L1 “don’t-kill-me” ligand, cytotoxic CD8+ T cells (CTLs) that may or may not express PD-1, regulatory T cells (Treg), and pro- and anti-inflammatory (M1 and M2) macrophages. These are the minimal set of immune cells known to be heavily involved in SCLC lung lesions (Kohli et al. 2022; Li et al. 2022).

Second are continuous agents that are secreted, consumed, or diffuse at a fast timescale (Δt=4 seconds). Rules governing continuous agents are discretized partial differential equations (PDEs), allowing a slower timescale. Cells secrete or consume molecules, and rule-based cell–cell interactions occur with probabilities described in the following sections. The continuous agents in *TumorSim* include IFN-ɣ representing a pro-inflammatory factor (Dangaj et al. 2019; Han et al. 2023); and CCL2, CCL5, and CXCL9 as chemokines that direct cell migration (Dangaj et al. 2019; Zheng et al. 2021; Kohli et al. 2022). Further, we track O_2_ as a separate continuous agent that diffuses through the microenvironment and is consumed by cellular agents. We use O_2_ to infer a hypoxia marker, HIF1α that directs cells towards immunosuppressive phenotypes (Fischer et al. 2007; Palazon et al. 2017). We describe rules and equations in detail in the following subsections.

#### Initialization

Shortly after the initial malignant transformation, nascent tumor cells may be eliminated by the immune system during an *elimination phase* (Schreiber et al. 2011; Vesely et al. 2011). The elimination phase is likely short and known to involve many components that are outside of the scope of this study, including CD4+ T cells and $\upgamma \updelta$ T cells. For this reason, we initiate our virtual SCLC tumor several days after tumor transformation. As our initial state, we represent a small cluster of cancer cells in lung tissue after immune signals from the elimination phase have allowed for macrophages, Tregs, and CTLs to begin to be recruited (Vesely et al. 2011).

*TumorSim* tumors begin as small circular lesions with a 10-cell diameter placed upon a 6 mm x 6 mm 2D simulation grid. The grid is divided into 20 μm × 20 μm compartments; this size allows one macrophage, T cell, or cancer cell to occupy a grid compartment as in previous cancer ABMs (Menezes et al. 2022; Calopiz et al. 2024). The entire lung grid section is initially populated by tissue densities of D_CTL_ cytotoxic T cells (CTLs), D_Treg_ regulatory T cells (Tregs), and macrophages (D_M1_ for M1 and D_M2_ for M2). Cancer cells are placed in a grid compartment in a circle of diameter *d*_Tumor_ centered in the middle of the tissue grid. We also randomly distribute vascular sources onto the 2D grid with a density D_VS_. This is the density we vascularize the lung with for *GranSim* simulations, as the lung tissue is the same (Pienaar et al. 2015a, b). Simulations initialize with both CTLs and cancer cells as PD-1- and PD-L1-, respectively, as SCLC is characterized by low levels of these markers (Pietanza et al. 2015). Initial conditions are shown in Table 2.

**Table 2:** Initialization parameters

Parameter	Value/Range	Description
*d*_Tumor_	10 cancer cells (200 μm)	Initial tumor diameter
D_CTL_	[0.0005, 0.0008]	Initial density of CTLs
D_Treg_	[0.0005, 0.0008]	Initial density of Treg
D_M1_	[0.00003, 0.00005]	Initial density of M1 macrophages
D_M2_	[0.0007, 0.001]	Initial density of M2 macrophages
D_VS_	0.0144	Initial density of vascular sources

Ranges for initial densities of immune cells have been inherited from *GranSim*, as both models share initial densities that reflect the immune environment of otherwise-healthy lung tissue

#### T Cells, Cancer Cells, and the PD-1/PD-L1 Axis

The levels of cytotoxic and regulatory T cells associated with tumors, as well as their exposure to pro/anti-inflammatory cytokines, are strongly associated with tumor prognosis (Chaudhary et al. 2016; Dangaj et al. 2019; Han et al. 2023). T cells may express the programmed cell death protein 1 (PD-1) as a surface protein, and tumors cells may produce its ligand (PD-L1). This receptor-ligand pair acts as a natural brake to prevent T cells from killing healthy tissue, but PD-L1 expression by cancer cells inhibits the ability of T cells to kill cancer cells (Wu et al. 2019). HIF1α, produced by cells during hypoxia, induces expression of PD-1 and PD-L1 in CTL (Palazon et al. 2017) and cancer cells (Feng et al. 2017), respectively. Conversely, interleukin-12 signaling induces expression of the transcription factor T-bet, which both directly represses PD-1 transcription and promotes IFN-ɣ production (Hodge-Dufour et al. 1998; Afkarian et al. 2002; Yang et al. 2007; Kao et al. 2011; Burke et al. 2019). Similarly, interleukin-2 indirectly regulates the expression of PD-L1 on cancer cells via CTLs, which has been modeled previously in detail (Gong et al. 2017).

In *TumorSim*, we coarse-grain each of the above interactions as events that stochastically occur during each large-scale timestep. We represent CTLs, Tregs, and cancer cells as discrete agents in our simulated tissue grid. Secretion of the continuous agents IFN-ɣ and HIF1α are described in Sects. 2.1.4 and 2.1.4, respectively. CTLs may be in the discrete states (i) activated or inactive, and (ii) PD-1+ or PD-1-; and cancer cells may be PD-L1+ or PD-L1-. Inactive CTLs may become activated with higher IFN-ɣ or when they are adjacent to (i.e. within the Moore neighborhood of) a cancer cell. An activated CTL adjacent to a cancer cell may kill that cancer cell with a probability based on whether the CTL is PD-1± and/or the cancer cell is PD-L1±. Tregs may deactivate adjacent CTLs. CTLs can induce PD-L1 expression in adjacent cancer cells. PD-1+ CTLs can be deactivated by PD-L1+ cancer cells. High levels of IFN-ɣ may result in the removal of PD-1 from the surface of CTLs. Equations 1–4 and Table 3 parameterize these interactions:

**Table 3:** T-cell parameters

Parameter	Value/Range	Description
P_actPD1-CTLKillingPDL+Tumor_	*[0.01, 0.1]*^*†*^	Probability of activated PD-1- CTL killing adjacent PD-L1+ cancer cells
P_actPD1+CTLKillingPDL+Tumor_	0	Probability of activated PD-1+ CTL killing adjacent PD-L1+ cancer cells
P_actPD1-CTLKillingPDL-Tumor_	*[0.1, 1]*^*†*^	Probability of activated PD-1- CTL killing adjacent PD-L1- cancer cells
P_actPD1+CTLKillingPDL-Tumor_	*[0.01, 0.1]*^*†*^	Probability of activated PD-1+ CTL killing adjacent PD-L1- cancer cells
P_TregDeactCTL_	*[0.1, 0.9]*	Probability of Treg deactivating adjacent CTL
P_CTL_tumorPDL1_	*[0.01, 1]*^*†*^	Probability of CTL inducing PD-L1 expression in adjacent cancer cells
P_tumorPDL1deactivatingPD1+CTL_	*[0.01, 1]*^*†*^	Probability of PD-L1+ cancer cell deactivating adjacent active PD-1+ CTL
P_CTLActivationByTumor_	*[0.01, 1]*^*†*^	Probability of CTL activation adjacent to a cancer cell
Vmax_CTLActivation_	*[0.1, 0.9]*	Max IFN-ɣ-dependent CTL activation probability
C50_CTLActivation_	[0.5, 3]*	Half-saturation molecule count for IFN-ɣ-dependent CTL activation
Vmax_PD-PD+CTLConversion_	*[0.01, 1]*^*†*^	Max HIF1α-dependent PD-1- → PD-1+ CTL conversion probability
C50_PD-PD+CTLConversion_	*[0.5, 0.9]*	Half-saturation relative concentration for PD-1- → PD-1+ HIF1α-dependent CTL conversion
Vmax_PD+PD-CTLConversion_	*[0.01, 1]*^*†*^	Max IFN-ɣ -dependent PD-1+ → PD-1- CTL conversion probability
C50_PD+PD-CTLConversion_	[0.5, 3]*	Half-saturation relative concentration for PD-1+ → PD-1- IFN-ɣ -dependent CTL conversion
Vmax_PDL-PDL+TumorConversion_	*[0.01, 1]*	Max HIF1α-dependent PD-L1- → PD-L1+ cancer cell conversion probability
C50_PDL-PDL+TumorConversion_	*[0.5, 0.9]*	Half-saturation relative concentration for PD-L1- → PD-L1+ HIF1α-dependent cancer cell conversion

Ranges in *italics* are broad-range estimates to explore model capability. *We established these exploratory ranges by finding the maximum and minimum IFN-ɣ molecule counts per grid compartment in pilot simulations. †We sample this range using a log-uniform distribution

1 $${p}_{TcytActivation}\left({C}_{IFN\gamma }\right)=\frac{{\mathrm{V}\mathrm{m}\mathrm{a}\mathrm{x}}_{\mathrm{C}\mathrm{T}\mathrm{L}\mathrm{A}\mathrm{c}\mathrm{t}\mathrm{i}\mathrm{v}\mathrm{a}\mathrm{t}\mathrm{i}\mathrm{o}\mathrm{n}}.{C}_{IFN\gamma }}{{C}_{IFN\gamma }+{\mathrm{C}50}_{\mathrm{C}\mathrm{T}\mathrm{L}\mathrm{A}\mathrm{c}\mathrm{t}\mathrm{i}\mathrm{v}\mathrm{a}\mathrm{t}\mathrm{i}\mathrm{o}\mathrm{n}}}$$

2 $${p}_{\mathrm{P}\mathrm{D}-\mathrm{P}\mathrm{D}+\mathrm{C}\mathrm{T}\mathrm{L}\mathrm{C}\mathrm{o}\mathrm{n}\mathrm{v}\mathrm{e}\mathrm{r}\mathrm{s}\mathrm{i}\mathrm{o}\mathrm{n}}\left({C}_{HIF1\alpha }\right)=\frac{{\mathrm{V}\mathrm{m}\mathrm{a}\mathrm{x}}_{\mathrm{P}\mathrm{D}-\mathrm{P}\mathrm{D}+\mathrm{C}\mathrm{T}\mathrm{L}\mathrm{C}\mathrm{o}\mathrm{n}\mathrm{v}\mathrm{e}\mathrm{r}\mathrm{s}\mathrm{i}\mathrm{o}\mathrm{n}}.{C}_{HIF1\alpha }}{{C}_{HIF1\alpha }+{C50}_{\mathrm{P}\mathrm{D}-\mathrm{P}\mathrm{D}+\mathrm{C}\mathrm{T}\mathrm{L}\mathrm{C}\mathrm{o}\mathrm{n}\mathrm{v}\mathrm{e}\mathrm{r}\mathrm{s}\mathrm{i}\mathrm{o}\mathrm{n}}}$$

3 $${p}_{\mathrm{P}\mathrm{D}+\mathrm{P}\mathrm{D}-\mathrm{C}\mathrm{T}\mathrm{L}\mathrm{C}\mathrm{o}\mathrm{n}\mathrm{v}\mathrm{e}\mathrm{r}\mathrm{s}\mathrm{i}\mathrm{o}\mathrm{n}}\left({C}_{IFN\gamma }\right)=\frac{{\mathrm{V}\mathrm{m}\mathrm{a}\mathrm{x}}_{\mathrm{P}\mathrm{D}+\mathrm{P}\mathrm{D}-\mathrm{C}\mathrm{T}\mathrm{L}\mathrm{C}\mathrm{o}\mathrm{n}\mathrm{v}\mathrm{e}\mathrm{r}\mathrm{s}\mathrm{i}\mathrm{o}\mathrm{n}}.{C}_{IFN\gamma }}{{C}_{IFN\gamma }+{C50}_{\mathrm{P}\mathrm{D}+\mathrm{P}\mathrm{D}-\mathrm{C}\mathrm{T}\mathrm{L}\mathrm{C}\mathrm{o}\mathrm{n}\mathrm{v}\mathrm{e}\mathrm{r}\mathrm{s}\mathrm{i}\mathrm{o}\mathrm{n}}}$$

4 $${p}_{\mathrm{P}\mathrm{D}\mathrm{L}-\mathrm{P}\mathrm{D}\mathrm{L}+\mathrm{T}\mathrm{u}\mathrm{m}\mathrm{o}\mathrm{r}\mathrm{C}\mathrm{o}\mathrm{n}\mathrm{v}\mathrm{e}\mathrm{r}\mathrm{s}\mathrm{i}\mathrm{o}\mathrm{n}}\left({C}_{HIF1\alpha }\right)=\frac{{\mathrm{V}\mathrm{m}\mathrm{a}\mathrm{x}}_{\mathrm{P}\mathrm{D}\mathrm{L}-\mathrm{P}\mathrm{D}\mathrm{L}+\mathrm{T}\mathrm{u}\mathrm{m}\mathrm{o}\mathrm{r}\mathrm{C}\mathrm{o}\mathrm{n}\mathrm{v}\mathrm{e}\mathrm{r}\mathrm{s}\mathrm{i}\mathrm{o}\mathrm{n}}.{C}_{HIF1\alpha }}{{C}_{HIF1\alpha }+{\mathrm{C}50}_{\mathrm{P}\mathrm{D}\mathrm{L}-\mathrm{P}\mathrm{D}\mathrm{L}+\mathrm{T}\mathrm{u}\mathrm{m}\mathrm{o}\mathrm{r}\mathrm{C}\mathrm{o}\mathrm{n}\mathrm{v}\mathrm{e}\mathrm{r}\mathrm{s}\mathrm{i}\mathrm{o}\mathrm{n}}}.$$

#### Macrophages

Tumor-associated macrophages are a key component of SCLC and their control (Wang et al. 2019; Zheng et al. 2021; Nabet et al. 2024). They can either be pro-inflammatory and help destroy the tumor or be anti-inflammatory and facilitate tumor growth. They also secrete chemokines important to T-cell recruitment (Sect. 2.1.4). We represent proinflammatory (M1) and anti-inflammatory (M2) macrophages that have been shown to affect tumor progression (Biswas et al. 2008). M1 macrophages are able to kill cancer cells when adjacent to a cancer cell and M2 macrophages are able to deactivate adjacent CTLs. M1 can convert to the M2 phenotype in the presence of high HIF1α levels, as products downstream of HIF1α are associated with M2 polarization (Colegio et al. 2014). M2 can be converted to M1 in the presence of high levels of IFN-ɣ (Nathan et al. 1983). Equations 5–6 and Table 4 describe polarization:

**Table 4:** Macrophage parameters

Parameter	Value/Range	Description
p_M1killingTumor_	*[0.1, 0.9]*	Probability of M1 killing adjacent cancer cell
p_M1activatingCTL_	*[0.1, 0.9]*	Probability of M1 activating adjacent CTL
p_M2deactivatingCTL_	*[0.1, 0.9]*	Probability of M2 deactivating adjacent CTL
Vmax_M1M2conversion_	*[0.1, 0.9]*	Max HIF1α-dependent M1→M2 conversion probability
C50_M1M2conversion_	*[0.1, 0.9]*	Half-saturation parameter for M1→M2 HIF1α-dependent conversion
Vmax_M2M1conversion_	*[0.1, 0.9]*	Max IFN-ɣ-dependent M2→M1 conversion probability
C50_M2M1conversion_	*[0.5, 3]*	Half-saturation molecule count for M2→M1 IFN-ɣ-dependent conversion

Ranges in *italics* are a broad-range estimate to explore model capability. We established these exploratory ranges by finding the maximum and minimum IFN-ɣ molecule counts per grid compartment in pilot simulations

5 $${p}_{M2M1conversion}\left({C}_{IFN\gamma }\right)=\frac{{\mathrm{V}\mathrm{m}\mathrm{a}\mathrm{x}}_{\mathrm{M}2\mathrm{M}1\mathrm{c}\mathrm{o}\mathrm{n}\mathrm{v}\mathrm{e}\mathrm{r}\mathrm{s}\mathrm{i}\mathrm{o}\mathrm{n}}.{C}_{IFN\gamma }}{{C}_{IFN\gamma }+{\mathrm{C}50}_{\mathrm{M}2\mathrm{M}1\mathrm{c}\mathrm{o}\mathrm{n}\mathrm{v}\mathrm{e}\mathrm{r}\mathrm{s}\mathrm{i}\mathrm{o}\mathrm{n}}}$$

6 $${p}_{M1M2conversion}\left({C}_{HIF1\alpha }\right)=\frac{{\mathrm{V}\mathrm{m}\mathrm{a}\mathrm{x}}_{\mathrm{M}1\mathrm{M}2\mathrm{c}\mathrm{o}\mathrm{n}\mathrm{v}\mathrm{e}\mathrm{r}\mathrm{s}\mathrm{i}\mathrm{o}\mathrm{n}}.{C}_{HIF1\alpha }}{{C}_{HIF1\alpha }+{\mathrm{C}50}_{\mathrm{M}1\mathrm{M}2\mathrm{c}\mathrm{o}\mathrm{n}\mathrm{v}\mathrm{e}\mathrm{r}\mathrm{s}\mathrm{i}\mathrm{o}\mathrm{n}}}$$

#### Chemokine-Cytokine Secretion and Chemotaxis

Critical components of immune control of SCLC tumors include cytokines and chemokines that activate immune cells and guide cells toward the developing tumor (Schreiber et al. 2011; Kohli et al. 2022). In *TumorSim*, we represent both IFN-ɣ and several chemokines as continuous variables (units of #molecules/grid compartment) that diffuse through tissue and are eventually cleared through degradation. Proinflammatory cells (M1 macrophages and CTLs) secrete IFN-ɣ at the fast timestep (4 s). IFN-ɣ secretion from CTLs decreases with higher HIF1α, as downstream products decrease IFN-ɣ secretion from CD8+ T cells (the most common form of CTL) (Fischer et al. 2007). IFN-ɣ secretion by M1 macrophages occurs at a constant rate, though we assume that macrophage production of IFN-ɣ is less than that of CTLs (Han et al. 2023).

We also assume that cancer cells constitutively produce the chemokines CCL2 and CCL5, known to initially recruit monocytes and T cells, respectively. We also assume that IFN-ɣ can induce M1 macrophages to produce CXCL9, another chemokine associated with CTL responses (Guttman et al. 2016; Dangaj et al. 2019; Kohli et al. 2022). Tregs also migrate in response to CXCL9 and CCL5 (Chaudhary et al. 2016; Moreno Ayala et al. 2023). We allow virtual M1 and M2 macrophages to follow CCL2 and CCL5 gradients, though we note this simplification serves to coarse-grain this chemoattraction prior to polarization (Deci et al. 2018). There is evidence that SCLC silences CCL2 (Zheng et al. 2021) and CCL5 (Dangaj et al. 2019), leading to an affected cytotoxic T-cell response that may undermine PD-1/PD-L1 blockade treatment, so we vary production rates of CCL2 and CCL5 to study this further. Table 5 and Eqs. 7–8 characterize these interactions:

7 $$ s_{{{\mathrm{IFN}}\gamma_{{{\mathrm{CTL}}}} }} \left( {C_{HIF1\alpha } } \right) = {\mathrm{Vmax}}_{{IFN\gamma_{{T{\mathrm{cyt}}}} }} \cdot \left( {\frac{{{\mathrm{C}}50_{{{\mathrm{IFN}}\gamma_{{{\mathrm{CTL}}}} }} }}{{C_{HIF1\alpha } + {\mathrm{C}}50_{{{\mathrm{IFN}}\gamma_{{{\mathrm{CTL}}}} }} }}} \right). $$

8 $$ s_{{CLXCL9_{M1} }} \left( {C_{IFN\gamma } } \right) = {\mathrm{Vmax}}_{CXCL9} \cdot \left( {\frac{{C_{IFN\gamma } }}{{C_{IFN\gamma } + {\mathrm{C}}50_{{{\mathrm{CXCL}}9}} }}} \right). $$

**Table 5:** IFN-ɣ and chemokine parameters

Parameter	Value/Range	Description
Vmax_IFNɣ_CTL_	*[0.1, 1]* (1/s)	Max IFN-ɣ secretion rate from cytotoxic T cells
C50_IFNɣ_CTL_	*[0.1, 0.9]*	Half-saturation parameter for HIF1α-dependent IFN-ɣ secretion rate
s_IFNɣ_M1_	*[0, 0.01]* (1/s)	IFN-ɣ secretion rate from M1 macrophages
D_IFNɣ_	1.4e−8 (cm^2^/s)	Diffusion coefficient of IFN-ɣ
μ_IFNɣ_	0.0158* (1/s)	Clearance rate of IFN-ɣ
Vmax_CXCL9_	12* (1/s)	Maximum secretion rate of CXCL9 by M1 macrophages
C50_CXCL9_	90	Half-saturation molecule count for IFN-ɣ-dependent CXCL9 secretion rate by M1 macrophages
s_CCL2_	*[0,0.1]* (1/s)	Rate of CCL2 secretion by cancer cells
s_CCL5_	*[0,0.1]* (1/s)	Rate of CCL5 secretion by cancer cells
D_CK_	5.2e−08 (cm^2^/s)	Diffusion coefficient of CCL2, CCL5, and CXCL9
μ_CK_	0.00158* (1/s)	Clearance rate of CCL2, CCL5, and CXCL9

Ranges in *italics* are broad-range estimates to explore model capability. *Value adapted from *GranSim*

#### Proliferation

SCLC cancer cells are characterized by rampant proliferation (Sen et al. 2025). In *TumorSim*, cancer cells proliferate based on a proliferation time parameter, which determines the time interval needed between two consecutive proliferation events (Table 6). Doubling times for several rapidly-dividing cancer cell types such as glioblastoma and HeLa are between 18–33 h in vitro (Newton et al. 1959; Oraiopoulou et al. 2017). However, doubling times for SCLC tumors have been measured to be between 25 and 217 days, albeit many such measurements are made by the time the tumors are at the cm-scale (Harris et al. 2012). We assume that in vivo conditions will prolong doubling time and investigate virtual SCLC cancer cells with doubling times of 36–48 h. Additionally, some immune cells are known to proliferate in response to cancerous tumor microenvironments (Hombach et al. 2007; Yoon et al. 2010; Blessin et al. 2021). We allowed a fraction of immune cells to proliferate based on activation state with cell cycle lengths estimated from literature (as referenced in Table 6). Notably, CTLs are known to have cell cycle durations as short as 2 h that slow substantially after several division rounds (Yoon et al. 2010; Kretschmer et al. 2023); we choose an 8-h cell cycle time to account for this eventual slowing. Table 6 characterizes *TumorSim* proliferation.

**Table 6:** Simulated cell cycle durations and frequency of proliferating phenotype by cell type

Parameter	Value/Range	Description
r_tumor_	[1.5, 2] days	Cancer cell doubling time
p_tumor_	[0.65, 1]	Probability that cancer cells are proliferating Ishibashi et al. (2017)
r_Treg_	5 days	Treg proliferation interval Hombach et al. (2007)
p_Treg_	0.03	Probability that Tregs are proliferating Blessin et al. (2021)
r_CTL_rest_	7.5 days	Inactivated CTL proliferation interval Yoon et al. (2010)
p_CTL_rest_	0.01	Probability that resting CTLs are proliferating Blessin et al. (2021)
r_CTL_act_	8 h	Activated CTL proliferation interval Yoon et al. (2010), Kretschmer et al. (2023)
p_CTL_act_	0.05	Probability that active CTLs are proliferating Blessin et al. (2021)
r_M1_	1.5 days	M1 macrophage proliferation interval *
r_M2_	2 days	M2 macrophage proliferation interval *
p_M_	[0.05, 0.1]	Probability that M1 or M2 macrophages are proliferating *

We estimated cell cycle lengths from the references cited, though true cell cycle durations are dynamically based on many factors that lie beyond the scope of this simulation. Values marked with * are an ansatz, as we could not find specific replication rates in the literature; however, macrophage replication in tumors has been reported (Filiberti et al. 2022)

#### Recruitment

Chemokines associated with inflammation and hypoxia recruit immune cells to the tumor microenvironment (Kohli et al. 2022). In *TumorSim*, each vascular source has a probability to recruit immune cells based on levels of local chemokines. The chemokines influencing these probabilities are based on identical reasoning to the chemotaxis model (Deci et al. 2018; Kohli et al. 2022). We assume that both M1 and M2 macrophages are recruited by CCL2 and CCL5, Tregs are recruited by CCL5, and CTLs are recruited by both CCL5 and CXCL9. Newly-recruited CTLs are activated, as we assume activation within lymph nodes occurs prior to their arrival in the tumor microenvironment (Iwai et al. 2021). Table 7 and Eqs. 9–11 characterize recruitment:

9 $$
\begin{aligned}{p}_{re{c}_{M1}}\left({C}_{CCL2}, {C}_{CCL5}\right)= {p}_{re{c}_{M2}}\left({C}_{CCL2}, {C}_{CCL5}\right)=b\cdot \frac{{\mathrm{R}\mathrm{m}\mathrm{a}\mathrm{x}}_{M}}{2}\left(\frac{{C}_{CCL2}}{{C}_{CCL2}+{C50}_{M}}+\frac{{C}_{CCL5}}{{C}_{CCL5}+{C50}_{M}}\right)\end{aligned}
$$

10 $$
{p}_{re{c}_{Tcyt}}\left({C}_{CCL5}, {C}_{CXCL9}\right)=b\cdot \frac{{\mathrm{R}\mathrm{m}\mathrm{a}\mathrm{x}}_{\mathrm{C}\mathrm{T}\mathrm{L}}}{2}\left(\frac{{C}_{CCL5}}{{C}_{CCL5}+{\mathrm{C}50}_{\mathrm{C}\mathrm{T}\mathrm{L}}}+\frac{{C}_{CXCL9}}{{C}_{CXCL9}+2\cdot {\mathrm{C}50}_{\mathrm{C}\mathrm{T}\mathrm{L}}}\right)
$$

11 $${p}_{re{c}_{Treg}}\left({C}_{CCL5}\right)=b\cdot {\mathrm{R}\mathrm{m}\mathrm{a}\mathrm{x}}_{\mathrm{T}\mathrm{r}\mathrm{e}\mathrm{g}}\frac{{C}_{CCL5}}{{C}_{CCL5}+{\mathrm{C}50}_{\mathrm{T}\mathrm{r}\mathrm{e}\mathrm{g}}}$$

where *b* is the blockage term $Blockage({C}_{HIF1\alpha })$ discussed in the next section. The factor of 1/2 in Eqs. 9–10 ensures that each chemokine contributes at most half of the maximal recruitment rate, so the total recruitment probability saturates at Rmax when both chemokines are at high concentration. For CTL recruitment (Eq. 10), the half-saturation for CXCL9 is doubled relative to CCL5. Recruitment at each vascular source is competitive: at most one immune cell type is recruited per source per timestep.

**Table 7:** Recruitment parameters

Parameter	Value/Range	Description
Rmax_M_	[0.0058, 0.025]	Max recruitment probability of M1 and M2
Rmax_CTL_	[0.012, 0.063]	Max recruitment probability of CTL
Rmax_Treg_	[0.0094, 0.041]	Max recruitment probability of Treg
C50_M_	[2.37, 4.62]	Half-saturation molecule count for M1 and M2 chemokine-dependent recruitment
C50_CTL_	[4.76, 10.27]	Half-saturation molecule count for CTL chemokine-dependent recruitment
C50_Treg_	[0.94, 2.0]	Half-saturation molecule count for Treg chemokine-dependent recruitment

Ranges are broad-range estimates to explore model capability

#### Vascular Permeability Modeling

Hypoxia, which frequently accompanies solid tumor development, results in vascular blockage that inhibits the ability of a vascular source to supply immune cells to the tumor microenvironment. In *TumorSim*, we model vascular permeability by calculating the blockage for each vascular source, which depends on levels of hypoxia at the source’s location (Economopoulou et al. 2009). To assess the level of hypoxia, we calculate O_2_ concentration at steady state within the whole grid using the heat equation and periodic boundary conditions after we initialize an n-by-n grid with the cancer cells, immune cells and vascular sources as

12 $$\left(\frac{{D}_{O2}}{\Delta {x}^{2}}A-\uplambda I\right)u=f$$

where ${D}_{O2}$ is the diffusion coefficient of O2, Δx is the length of one grid compartment, A is an n^2^-by- n^2^ Laplacian matrix for periodic boundary conditions, u is an n^2^-by- 1 vector of O2 concentrations at the simulation grid and f is the n^2^-by-1 / vector for the O2 contribution from sources and sinks (see Table 8 parameter descriptions and values). We define the forcing vector f per grid compartment i as:

13 $$
{f}_{i}\!=\! \left\{\begin{array}{l}-\uplambda \!\cdot\! {O2}_{grid}\\ -\uplambda \cdot {O2}_{grid}-{fO2}_{VS}\cdot Blockage\left({C}_{HIF1\alpha }\right)\\ -\uplambda \cdot {O2}_{grid}+{cO2}_{j}\end{array}\right. \begin{array}{l}\text{if the grid} \ i \ \text{does not have any sources or sinks}\\ \text{if grid} \ i \ \text{has a vascular source}\\ \text{if grid} \ i \ \text{has a cell type that consumes O2}\end{array}
$$

where oxygen consuming cells are Cancer cells, CTLs, Tregs, M1 macrophages, and M2 macrophages, and $Blockage({C}_{HIF1\alpha })$ is defined below (Supplementary Material S1 for additional detail).

**Table 8:** Vascular permeability, O_2_, and HIF1α, and parameters

Parameter	Value/Range	Description
D_O2_	3.08e−5 (cm^2^/s)	Diffusion coefficient of O_2_
Δx	20 (μm)	Length of one grid compartment
λ*	0.3 (1/s)	Absorption rate constant
cO2_Tumor_*	6.7e7 (1/s)	Number of O_2_ molecules consumed by cancer cells per second
cO2_CTL_*	2.4e6 (1/s)	Number of O_2_ molecules consumed by CTL per second
cO2_Treg_*	2.4e6 (1/s)	Number of O_2_ molecules consumed by Treg per second
cO2_M1_*	[3.85e7, 6.7e7] (1/s)	Number of O_2_ molecules consumed by M1 per second
cO2_M2_*	[3.85e7, 6.7e7] (1/s)	Number of O_2_ molecules consumed by M2 per second
fO2_VS_*	1.7e11 (1/s)	Number of O_2_ molecules coming through vascular sources per second
O2_Grid_*	9.2e8 (1/s)	Number of O_2_ molecules diffusing from the inhaled air in the lungs to the lung tissue per microgrid per second
b_O2_	[30, 50]^†^	Half-saturation of O_2_ mg/L per grid compartment for HIF1⍺ conversion
h_O2_	*[1, 5]*	O_2_ to relative HIF1⍺ concentration conversion parameter
b_blockage_	*[0.1, 0.9]*	HIF1⍺ level associated with 50% vascular blockage
h_blockage_	*[0.5, 5.0]*	HIF1⍺ to vascular blockage conversion parameter

We present further derivation for parameters marked by * in Supplementary Material S1. †This parameter is based on preliminary simulations where approximately 20 mg/L was a common baseline concentration outside of the tumor

Hypoxia induces expression of HIF1α (Li et al. 2022). We model relative HIF1α concentration based on steady state O2 concentrations. Relative HIF1α concentration ${C}_{HIF1\alpha }$ is a unitless per-grid-compartment value between 0 and 1 that increases with hypoxia (i.e., lower O2 levels), and computed using the following equation (parameterized in Table 8):

14 $${C}_{HIF1\alpha }(O2)=1-\frac{{O2}^{{\mathrm{h}}_{\mathrm{O}2}}}{{O2}^{{\mathrm{h}}_{\mathrm{O}2}}+{{\mathrm{b}}_{\mathrm{O}2}}^{{\mathrm{h}}_{\mathrm{O}2}}}$$

We model vascular blockage based on relative HIF1α (Eq. 14, Table 8 for parameterization). We assume vascular sources that are exposed to higher HIF1α are increasingly blocked as higher HIF1α is associated with leaky vascularization via VEGF (Zhan et al. 2009). The blockage term is between 0 and 1 and is proportional to HIF1α and quantifies the proportion of molecules allowed to pass through each vascular source, where a value of 0 indicates no passage, 0.5 represents 50% passage, etc. (see ${f}_{i}$). Blockage is calculated as:

15 $$Blockage({C}_{HIF1\alpha })=\frac{{{C}_{HIF1\alpha }}^{{\mathrm{h}}_{\mathrm{b}\mathrm{l}\mathrm{o}\mathrm{c}\mathrm{k}\mathrm{a}\mathrm{g}\mathrm{e}}}}{{{C}_{HIF1\alpha }}^{{\mathrm{h}}_{\mathrm{b}\mathrm{l}\mathrm{o}\mathrm{c}\mathrm{k}\mathrm{a}\mathrm{g}\mathrm{e}}}+{{\mathrm{b}}_{\mathrm{b}\mathrm{l}\mathrm{o}\mathrm{c}\mathrm{k}\mathrm{a}\mathrm{g}\mathrm{e}}}^{{\mathrm{h}}_{\mathrm{b}\mathrm{l}\mathrm{o}\mathrm{c}\mathrm{k}\mathrm{a}\mathrm{g}\mathrm{e}}}}$$

### *GranSim*

The immune cellular compositions of lung tumors and granulomas are similar (Fang et al. 2025). To compare whether the most influential mechanisms underpinning them are also analogous, we present a brief summary of the components of *GranSim*, which captures key cell types that lead to emergent TB granuloma formation and maintenance. These interactions lead to the emergence of a single lung granuloma that is structurally and functionally representative of non-human primate granulomas, the animal TB model closest to human TB disease and also closely resembles human granulomas (Cardona et al. 2001, 2005; Marino et al. 2004; Segovia-Juarez et al. 2004; Gammack et al. 2005; Ray et al. 2009).

*GranSim* is a multiscale ABM simulating the interactions between immune cells and bacteria (Mtb) on a 2-D simulation grid representing a 6 mm x 6 mm lung tissue. *GranSim* uses discrete and continuous agents using slow and fast timescales (ΔT=10 minutes and Δt=6 seconds, respectively). Discrete *GranSim* agents include cytotoxic, IFN-ɣ secreting and regulatory T cells; macrophages, Mtb, and vascular sources that serve as loci for cellular recruitment. The grid is divided into 20 μm × 20 μm compartments, we admit a single compartment to contain one macrophage, two T cells, or one macrophage and one T cell. Continuous agents include chemokines (CCL2, CCL5 and CXCL9) and cytokines (TNF-α and IFN-ɣ, IL-10). Continuous agents are implemented as PDEs, and are updated using an FFT-based PDE solver as in our previous work (Cilfone et al. 2014).

Below is an overview of the major components of *GranSim*. For details and parameter values, see our online documentation at http://malthus.micro.med.umich.edu/GranSim/docs/gransimrules-v2.pdf.

#### Initialization

TB can be caused by inhaling a single bacterium (Martin et al. 2017). As such, we begin with a single macrophage infected with Mtb at the center of the simulation grid. Resting macrophages and vascular sources are randomly distributed onto the 2-D simulation grid, representing otherwise-healthy tissue. We have both slow-timescale (cellular) and fast-timescale (molecular) mechanisms.

#### Macrophages

Macrophages provide both the main replicative niche for Mtb and once activated, a primary source of bactericidal activity (Ramakrishnan 2012). Macrophages in *GranSim* assume one of four states depending on their activation and infection statuses: resting, activated, infected and chronically infected. Macrophages are in the ‘resting state’ when they are first recruited into the simulation grid. They switch into an ‘activated state’ when they are activated by STAT1 and NFKB-dependent mechanisms and can kill Mtb effectively. They get infected with a defined probability when they are adjacent to Mtb and switch into an ‘infected state’. Infected macrophages provide a permissive niche for Mtb that grow within macrophages. Once the number of Mtb within a macrophage reaches a predefined threshold, then the macrophage switches from the ‘infected state’ into the ‘chronically infected state’, where it cannot clear the Mtb or move due to highly infected status. Once the number of intracellular Mtb reaches the carrying capacity, the chronically infected macrophage bursts and dies.

#### T Cells

Mtb-specific T cells are critical for effective control of Mtb, both through inducing apoptosis of infected macrophages, activation of resting macrophages (allowing them to effectively kill Mtb) (Flynn et al. 2022; Winchell et al. 2023). Tregs cells are immunosuppressive, and their activity is associated with poor granuloma outcomes (Flynn et al. 2022). *GranSim* represents three types of T cells: cytotoxic (CTL), IFN-ɣ-secreting (Tgam), and regulatory (Treg). Cytotoxic T and IFN-ɣ-secreting T cells secrete TNF-α, but only IFN-ɣ-secreting T cells secrete IFN-ɣ. Cytotoxic T cells induce perforin/granulysin-mediated apoptosis, whereas IFN-ɣ -secreting T cells induce Fas/FasL mediated apoptosis in infected and chronically infected macrophages. Both mechanisms of apoptosis result in the death of the macrophage along with a portion of their intracellular Mtb. When adjacent, regulatory T cells can downregulate cytotoxic and IFN-ɣ -secreting T cells, which leads to the loss of their cytokine-secreting and apoptosis-inducing abilities.

#### Vascular Blockage Modeling

As cells in TB granulomas die, dead cellular debris accumulates into a necrotic core (caseum) that serves as a niche for bacterial survival but they cannot replicate; vascular sources are abnormal in caseum, leading to a hypoxic microenvironment (Datta et al. 2015). We model vascular blockage depending on the amount of necrotic tissue (i.e., caseum) and cell density at the vascular source’s location. The blockage increases with higher necrotization and higher density. We model the level of necrotization as the ratio of the number of dead cells on that location to a maximum of dead cells allowed in each grid. Once the number of dead cells reaches the maximum value allowed, then we consider the grid as caseated, which means that the vascular source at that location is completely blocked, and cells can no longer move to that location.

#### Mtb and Bacterial Proliferation

Mtb replicate based on their metabolic state, which differs based on their main niches: within-macrophages (intracellular Mtb), in the interstitial space between live cells (extracellular-replicating), and within caseum (extracellular-nonreplicating) (Ramakrishnan 2012). Each Mtb agent in *GranSim* is assigned a metabolic mode based on their location: intracellular Mtb within macrophages, extracellular Mtb in the cellular region of granulomas and nonreplicating Mtb trapped in the caseum. Intra- and extracellular Mtb proliferate in *GranSim*, but immune cells and nonreplicating Mtb do not proliferate. The total rate of proliferation of intracellular Mtb depends on the adaptive immune response and decreases when adaptive immunity is effective. The rate of proliferation of extracellular Mtb depends on the caseation level and the number of Mtb within a grid compartment, with replication rate decreasing with increased caseation and Mtb burden.

#### Chemokine-Cytokine Secretion

Similar to tumors, chemokines and cytokines are secreted within granulomas to activate, suppress, or chemoattract cells (Ramakrishnan 2012). In *GranSim*, activated, infected and chronically infected macrophages secrete the chemokines CCL2, CCL5 and CXCL9. Unless they are downregulated by Tregs, CTLs and Tgams are able to secrete cytokines—both CTLs and Tgams secrete TNF-α, while only Tgams cells secrete IFN-ɣ. Macrophages respond to the chemoattraction of CCL2 and CCL5, CTLs to CCL5 and CXCL9, Tregs along CCL5, and Tgam along all CCL2, CCL5, CXCL9.

#### Recruitment

Cytokines also inform the rate of recruitment of immune cells to the site of Mtb infection (Flynn et al. 2022). We recruit macrophages and T cells from vascular sources. The recruitment rates at each vascular source are dependent upon the concentrations of CCL2, CCL5, CXCL9, and TNF-α in the specified grid compartment similar to the description for *TumorSim*.

#### Age-Related Cell Death

As granulomas can persist for years (Lin et al. 2018), we allow simulated cells to age and eventually die. During initialization and recruitment, the age of immune cells is randomly sampled between zero and their maximum age. Immune cells die when they reach their corresponding maximum ages.

### Granuloma and Tumor Simulations

Though both *GranSim* and *TumorSim* share many immune components, the underlying biology suggests very different simulation endpoints. Unlike the immune-controlled growth of granulomas, aggressively-growing SCLC tumors continue to expand, often exceeding 3 cm in diameter (Wang et al. 2022). We expect that a virtual SCLC tumor without any immune resistance may exceed a 5 mm x 5 mm region within 15–30 days and overflow our simulated domain; for this reason, we simulate 2 weeks of tumor growth early after the tumor is established. By contrast, we simulate TB granulomas for 300 days to capture both early formation and late-stage maintenance.

To accurately understand how model outcomes depend on parameter values, we ensure that the simulations arising from our parameter ranges well-represent the underlying biology. To ensure a thorough exploration of parameter space, we employ the Latin hypercube sampling (LHS) method (Marino et al. 2008) that creates a stratified random sampling of a given parameter space (Marino et al. 2008; Renardy et al. 2021). For *TumorSim*, we generate 500 parameter sets using the parameter ranges described in Tables 2–8. For *GranSim*, we sample 500 parameter sets using parameter ranges calibrated in our previous work (Budak et al. 2023, 2024). To address stochasticity inherent to the ABM (i.e., aleatory uncertainty) in both *TumorSim* and *GranSim*, we perform 3 replicate simulations for each parameter sample for a total of 1500 virtual SCLC tumors and 1500 virtual granulomas.

### Classifying Granuloma and Tumor Outcomes

Several common terms have been used to describe tumor status. Uncontrolled proliferation of cancer cells within tumors is frequently described as tumor progression (Schreiber et al. 2011; Li et al. 2022; Kim et al. 2025). Lack of progression is typically characterized by progression-free survival of the patient. Tumor regression is clinically defined as the disappearance of a tumor (Horino et al. 2006). Tumors that neither progress nor regress are called dormant, whether that is due to a balance of pro- and anti-tumor effects, or a consequence of quiescent cells (Li et al. 2015). The vast majority of SCLC tumors progress, with few cases of regression ever reported (Everson 1964; Horino et al. 2006).

In *TumorSim*, we use simulated tumor volume to categorize simulated tumors into three analogous qualitative categories: progressing, dormant, or regressing. We compute tumor volume by counting the number of tumor-associated cells within the simulation. We then infer tumor radius by approximating the tumor area as a circle and estimating tumor volume by assuming a spherical tumor. We describe simulated tumors as progressing if volume increases near-exponentially, dormant if tumor volume plateaus, and regressing if the volume decreases or if all cancer cells die. Supplementary Material S2 shows examples of raw simulation output and tumor outcomes over time, including tumor volume and the sizes of several cell subpopulations.

Similarly, common terms exist to describe TB granulomas. However, granuloma volume is driven by leukocyte count and the bulk of caseous necrosis, fibrosis, and calcification; macroscopic granulomas may be entirely sterile (Lin et al. 2014). Rather, granuloma status is described based on their ability to control Mtb, including sterile, poorly-controlling Mtb, or well-controlling Mtb (although caseum is associated with poor control) (Lin et al. 2014). Poorly-controlling granulomas are associated with the Mtb dissemination, causing disease severity and/or the foundation of new granulomas (Ramakrishnan 2012; Lin et al. 2014, 2018).

In *GranSim*, we use total Mtb count to classify simulated granulomas into one of three states: sterilizing, controlling, and disseminating. Sterilizing granulomas are those that completely clear Mtb within 300 days. Controlling granulomas are those that maintain stable, low levels of Mtb after the adaptive immune system is primed. Disseminating granulomas are those that fail to control Mtb replication, characterized by a continued increase of Mtb throughout the simulation. As with *TumorSim*, in Supplementary Material S2 we show examples of *GranSim* granuloma Mtb count, volumes, and sizes of cell subpopulations over time.

### Uncertainty and Sensitivity Analyses

Complex models like hybrid ABMs are often nonlinear and have hundreds of inputs. Uncertainty and sensitivity analysis allow us to explore parameters that significantly impact outputs of interest. To uncover which dynamics drive heterogeneity in SCLC tumor and TB granuloma outcomes, we employ the partial rank correlation coefficient (PRCC) method, a robust sensitivity analysis method that quantifies nonlinear monotonic relationships (Marino et al. 2008). Even small PRCC values may indicate significant correlations (Marino et al. 2008), and so we compute *p-*values associated with each parameter adjusted for multiple comparisons via the Benjamini–Hochberg correction (Gou 2023). We use the PRCC method to analyze the 1500 virtual SCLC tumors and 1500 virtual granulomas generated previously. To ensure that the new *TumorSim* model satisfies the monotonicity requirement of PRCC, we simulated scatterplots showing how *TumorSim* outcomes vary per individual parameters (see Supplementary Material S3).

## Results

Our goal is to compare and contrast the dynamics of SCLC tumors and TB granulomas using our models *TumorSim* and *GranSim*. One of the key differences between lung cancer and TB granulomas is the duration of disease: SCLC aggressively proliferates and can grow to cm-scale even prior to diagnosis (Wang et al. 2022), whereas many TB granulomas remain  < 1 cm even after months (Lin et al. 2014, 2018). To examine drivers of rapid tumor expansion, we have simulated SCLC tumors for 14 days after the initiation of the escape phase. By contrast, we simulate TB granulomas for 300 days to capture a spectrum of long-term controlling and non-controlling granulomas.

### *TumorSim* and *GranSim* Capture Spatiotemporal Features of SCLC Tumors and TB Granulomas

**Fig. 2:**
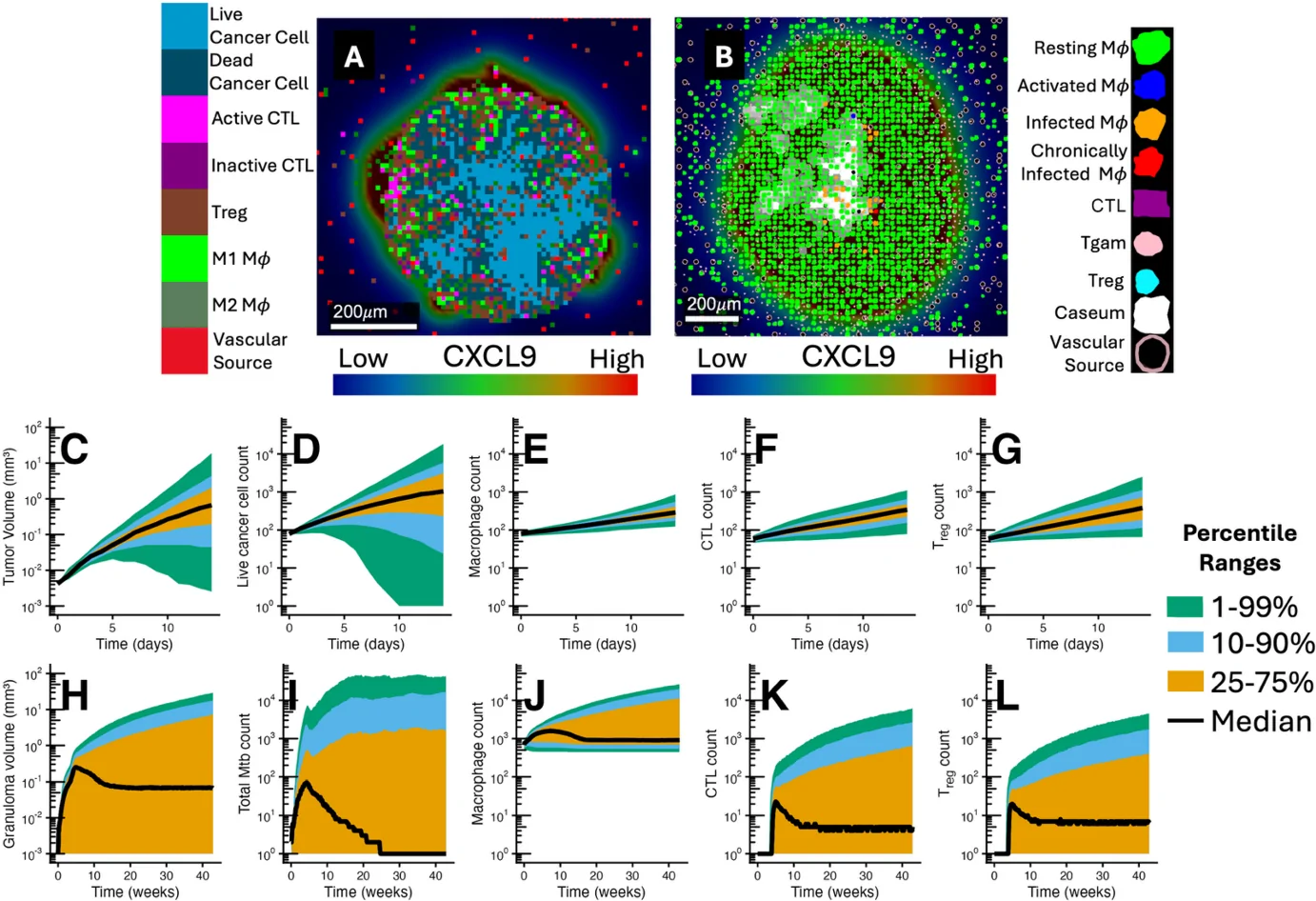
Spatial and Temporal *GranSim* and *TumorSim* model outputs for comparison and contrast. Both **A** *TumorSim* and **B** *GranSim* simulate spatial interactions of multiple cell types. Both scale bars are 200 microns. **A** A representative *TumorSim* SCLC tumor simulation of a lymphocyte-infiltrated tumor after 14 days of simulation. A mass of cancer cells (blue) is infiltrated by a collection of CTLs and macrophages, while some CTLs are being deactivated by Tregs We show relative levels of CXCL9 as a background heatmap. **B** A representative simulation of a non-sterile granuloma at day 300. Caseum has formed in the center of the infection site and is surrounded by macrophages as well as lymphocytes. We show relative levels of CXCL9 as a background heatmap. **A**–**B** We chose both simulations to demonstrate interaction of multiple cell types. **C**–**L** Each panel shows the median and percentile ranges for multiple temporal outcomes over time for both *TumorSim* **C**–**G** and *GranSim* **H**–**L**. Individual simulation trajectories used to generate these statistics are in Supplementary Fig. S4. Outcomes include lesion volume, Mtb/Cancer cell counts, and leukocyte counts *GranSim* simulations are run for 300 days to capture both early- and late-state dynamics. To plot outcomes on a log_10_ scale, we have added 1 to all cell counts and 10^−3^mm^3^ to lesion volumes. We simulate *TumorSim* virtual tumors for two weeks due to aggressive growth; we simulate *GranSim* virtual granulomas for 300 days to observe long-term Mtb infection outcomes (color figure online)

Here, we validate *TumorSim* by comparing gross simulation with reasonable expectations set by published data on lung lesions. In (Fig. 2), we also present side-by-side comparisons between *TumorSim* and *GranSim* outputs to compare and contrast. In Fig. 2A, we present a small lymphocyte-infiltrated SCLC tumor with an effective immune response as observed in human tumors (Chen et al. 2022). Supplementary Fig. S2 separates the agents of this tumor into separate panels. Activated CTLs are located near M1 macrophages and mostly at the tumor boundary as seen in vivo (Yang et al. 2025). We see deactivated CTLs near Tregs within the tumor. Most larger tumors had substantially fewer infiltrating leukocytes, growing essentially unchallenged by immune cells; such tumors are described elsewhere as "cold" and associated with poor outcomes (Galon et al. 2019; Zheng et al. 2021). While the majority of tumors continue to progress (Methods), a small number tend towards dormancy and regression (Fig. 2C). While events of spontaneous SCLC regression are exceedingly rare clinically (Horino et al. 2006), the small size of these simulated tumors (< ~ 200 cells) are likely more comparable to the elimination stage of tumor development that cannot be observed in vivo (Schreiber et al. 2011; Vesely et al. 2011). As tumors grow, so do total numbers of overall tumor-associated T cells and macrophages (Fig. 2C–G). By comparison, a majority of simulated TB granulomas are either controlling or sterilizing. Figure 2B shows a representative latent virtual granuloma, where the immune system controls but does not completely clear Mtb infection. Supplementary Fig. S3 separates the agents of this granuloma into separate panels. Necrotic caseum accumulates in the center of the Mtb-infected lesion. Overall, we observe an early spike in Mtb count due to the delay in the adaptive immune response, whereafter granuloma volume, Mtb count, macrophage, and T cell count stabilizes for most granulomas (Fig. 2H–L); this reflects the tendency for most granulomas to permit a persistent, latent Mtb infection state (Lin et al. 2018). *GranSim* simulations also capture active TB disease dynamics, as over 40% of our simulated granulomas disseminate, with increasing Mtb counts throughout the simulation (Fig. 2I). In Supplementary Figs. S5–S6, we present storyboard-like progressions of representative simulations for progressing tumors, dormant tumors, and regressing tumors; and non-controlling, controlling, and sterilizing granulomas, respectively. Supplementary Video S1 shows the development of the tumor in Fig. 2A, and Supplementary Videos S2 and S3 show the development of progressing and regressing tumors, respectively.

### *TumorSim* Captures Distinct Roles of Chemokines During Tumor Development

**Fig. 3:**
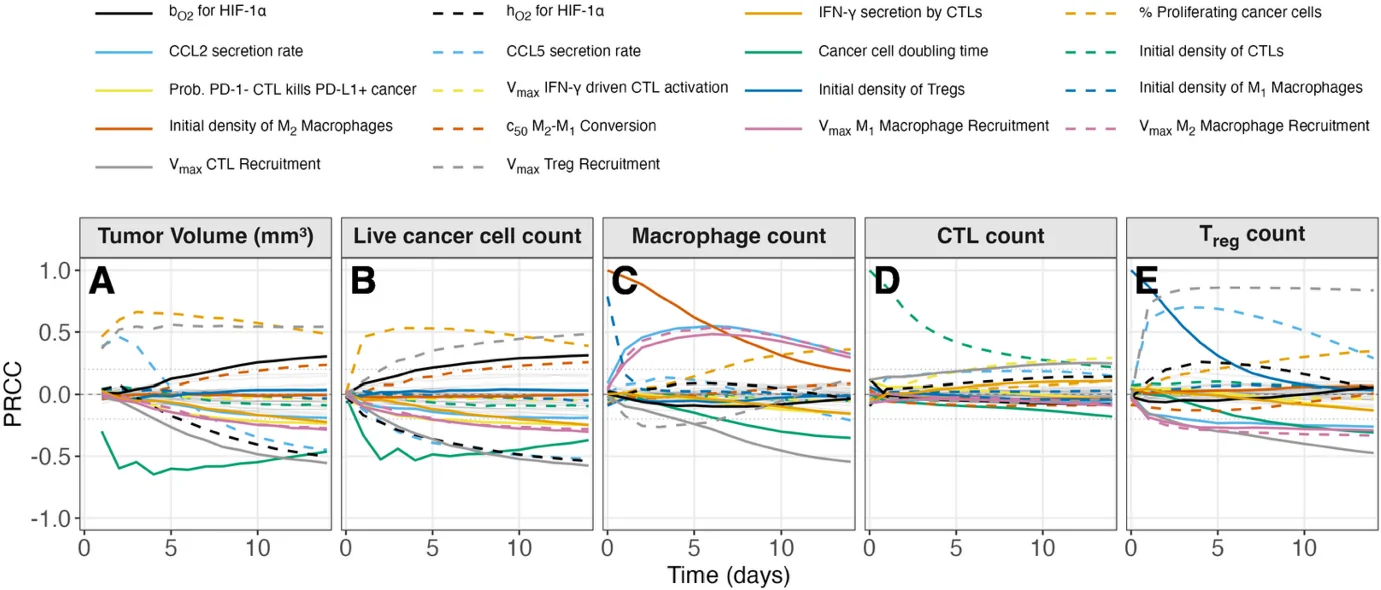
Parameters influencing *TumorSim* outcomes. To assess mechanisms that influence dynamics and outcomes of *TumorSim*, we present PRCC values over time for every varied *TumorSim* parameter (Methods). We have highlighted PRCC trajectories for those parameters that had significant correlation and |PRCC|> 0.2 for at least one outcome for one or more timepoints (*p* < 0.05 after Benjamini–Hochberg multiple comparison correction (Gou 2023)), while all other PRCC values are shown in gray. PRCC values presented capture the impact of parameters on the model outcomes: **A** tumor volume, **B** live cancer cell count, **C** number of macrophages, **D** CTL count, and **E** Treg count. Some C_50_ values were omitted from presentation for clarity when the associated V_max_/R_max_ parameters appeared as significant showing similar mechanistic impact (color figure online)

With the ability to mechanistically simulate 2 large sets of heterogeneous virtual granulomas and tumors, respectively, we analyze each model to determine which parameters (and thereby mechanisms) underpin the heterogeneity of outcomes observed during formation of the lesions. We measure partial rank correlation coefficients (PRCC; see Methods) to capture how parameters that we vary in Tables 2–8 impacted five different tumor-scale outcomes (Fig. 3). Throughout simulations, higher levels of Treg recruitment and faster cancer cell proliferation are associated with larger tumors. Beyond this, we notice a unique separation of early- and late-phase behavior of tumor growth that is driven by distinct mechanisms, occurring near the 1-week timepoint (Fig. 3). Early during tumor growth, macrophage and Treg counts correlate with CCL2 and CCL5 secretion rates in addition to their initial counts. During this time, CCL5 positively correlates with tumor volume—likely due to the recruitment of Tregs. This correlation becomes negative later during tumor growth, around the time that CTL killing of PD-L1+ becomes significantly negatively correlated with tumor volume. This later-phase shift also coincides with the time that CTL levels correlate with CTL activation probability—likely due to the importance of IFN-ɣ production in T-cell recruitment via CXCL9 production by M1 macrophages. While it is well-studied that both CCL5 and CXCL9 perform significant anti-tumor roles (Dangaj et al. 2019; Jin et al. 2024), our simulations suggest that CCL5 may serve a pro-tumor role early during tumor development by recruiting Tregs in the absence of CXCL9. It is during this later stage that PD-1- CTL killing of PD-L1+ cancer cells negatively correlates with tumor volume.

We also observe that macrophage populations negatively correlate with Treg recruitment, likely due to our assuming slower proliferation rates of M2 macrophages. We notice that as simulations progress, all cell populations correlate with faster cancer cell proliferation, likely due to higher net exposure of vascular sources to tumor-associated chemokines. Finally, we observe that as tumors develop, hypoxia-related parameters become more significant, with higher levels of HIF1α correlating with larger tumors, more cancer cells, larger Treg populations, and macrophage counts, likely due to increased CTL PD-1 expression. Lastly, we note that no initial conditions of macrophages or T cells showed as significant to tumor size, suggesting that long-term outcomes are dependent mostly on recruiting effects, which is consistent with development of adaptive immunity.

### Granuloma Outcomes are Determined by a Balance of Pro- and Anti-inflammatory Regulators

**Fig. 4:**
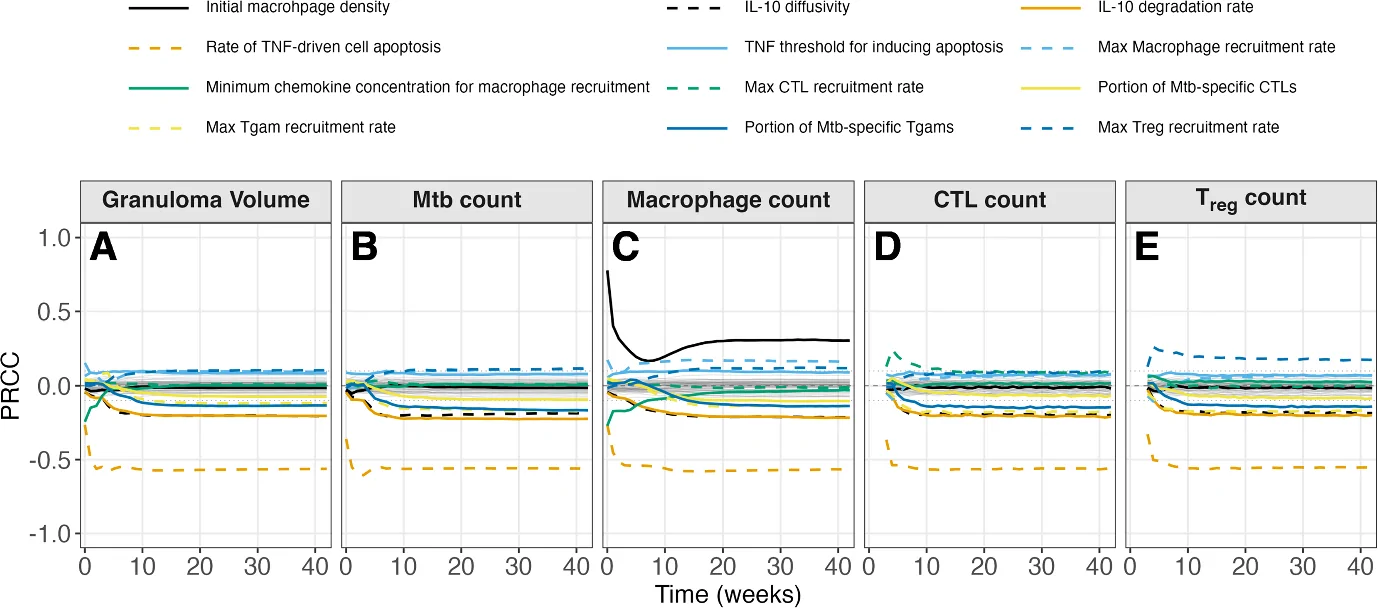
Parameters influencing *GranSim* outcomes. We present PRCC values over time for every varied *GranSim* parameter for several outcomes. We have highlighted PRCC trajectories for those parameters that had significant correlation for at least one outcome for at one or more timepoints (*p* < 0.05 with Benjamini–Hochberg multiple comparison correction), while all other PRCC values are shown in gray. PRCC values presented capture the impact of parameters on the model outcomes: **A** granuloma volume, **B** total live Mtb count, **C** number of macrophages, **D** CTL count, and **E** Treg count (color figure online)

From observing spatial and temporal dynamics it is clear there are similarities and differences between behaviors of SCLC and TB systems. To compare, we perform a parallel sensitivity analysis identifying what features control TB lesion outcomes in contrast to SCLC. As above, we apply the PRCC method to assess the impact of each mechanism on five *GranSim* outcomes analogous to *TumorSim* outcomes (Fig. 4). We observe overlapping mechanisms driving bacterial burden and granuloma size, meaning granulomas with higher bacterial burden tend to have larger sizes. Concentration of Interleukin-10 (IL-10), a key anti-inflammatory cytokine, shows significant correlation with larger granulomas and higher levels of Mtb, as observed previously (Cicchese JM et al. 2018). Similarly, lower rates of TNF-α-induced apoptosis, lower recruitment of Tgam, lower cognate Tgam ratio and higher recruitment of macrophages all lead to higher Mtb burden and larger granulomas during the course of infection, consistent with previous experimental and computational studies (Pitt et al. 2012; Cilfone et al. 2013; Cilfone et al. 2015a, b; Lin et al. 2016; Wong et al. 2020). In addition, granuloma size is also sensitive to the thresholds of chemokines needed for macrophage recruitment in the beginning of the infection, such that higher thresholds lead to smaller granulomas due to decreased macrophage recruitment early on. Notably, by around 22 weeks, the drivers of these dynamics settle and we observe no change in drivers of granuloma volume between weeks 22 and 43, despite poorly-controlling granulomas continuing to increase in volume (Fig. 2H).

## Discussion

Understanding how cell-scale behavior influences macroscopic outcomes is key to understanding the pathology of SCLC and which potential treatments are most promising. Though many mechanisms about SCLC are known, effective treatments remain elusive. In this work we introduce a novel molecule-to-tissue scale model of a lung lesion, *TumorSim*, an agent-based model of SCLC tumors rooted in key cellular and cytokine players to probe which mechanisms principally drive variability in SCLC tumor outcomes.

We base *TumorSim* on the computational framework of *GranSim*, our hybrid ABM representing pulmonary TB granuloma formation (Segovia-Juarez et al. 2004; Ray et al. 2009; Fallahi-Sichani et al. 2011; Cilfone et al. 2013). Lung tumors and TB granulomas share multiple similarities, both in terms of clinical diagnosis (Low et al. 2006; Werutsky et al. 2019) and known drivers of the granuloma/tumor immune microenvironment (Fang et al. 2025). Not surprisingly, data suggest that lung lesions are similar in many immune characteristics and even are mistaken for each other on lung examination (Fang et al. 2025). Implementation of *TumorSim* utilizes similarities and differences describing immune-interaction mechanisms detailed in *GranSim* (Table 1), including our grid-based, lung-tissue simulation environment, molecular- and cell-scale programming of macrophages by T cells in various (anti-)inflammatory states, and an updated oxygen diffusion algorithm. *TumorSim*-specific components include PD-1 and PD-L1 interactions between T cells and cancer cells, descriptions of distinct macrophage states and hypoxia-driven vasculature blockage. We validated *TumorSim* by recapitulating multiple known drivers of tumor outcomes, including the relative interdependence of tumor outcomes on cytotoxic T-cell-to-macrophage interactions via CCL5 and CXCL9 (Dangaj et al. 2019; Jin et al. 2024), as well as the impact of cytotoxic T-cell activity on reduced tumor volume.

Examining *TumorSim* dynamics suggests that tumor dynamics early during tumor escape may be distinct from dynamics driving more mature tumors. Simulations predict that increased CCL5 serves a pro-tumor role during early tumor development by recruiting regulatory T cells before macrophages can mediate recruitment of cytotoxic T cells. During this early phase, Tregs protect the tumor before hypoxia-driven mechanisms become relevant. These could serve as targets for immune boosting treatments of tumors.

We observed several similarities and differences between SCLC tumors and TB granulomas. First, absolute numbers of all cell types—both pro-lesion and anti-lesion—appeared to scale with lesion size. Potent negative regulators (e.g., PD-1/PD-L1 in SCLC; IL-10 in TB; Tregs in both) had consistent impacts on lesion volume and cancer cell/Mtb count. By contrast, TB granulomas are much more effectively limited by the replicative niche of macrophages even when infection is not cleared; whereas non-dormant SCLC lack such constraints and can replicate indefinitely. This is reflected in SCLC proliferation rate correlating with tumor volume, while Mtb replication rate has little correlation with overall granuloma volume or Mtb count.

One goal of this study was to compare and contrast what we learn about both lesion types and their driving mechanisms for intervention purposes. Both *TumorSim* and *GranSim* show improved outcomes with increased cytotoxic activity: tumor volume negatively correlates with CTL and M1 macrophage recruitment (Fig. 3), while granuloma Mtb count negatively correlates with Tgam, CTL, and bactericidal macrophage activity (Fig. 4). Outcomes worsen with the action of regulatory T cells, as Tregs correlate with both increased tumor and granuloma volume. This suggests that targeted inhibition of Treg recruitment or activity could improve both SCLC and TB outcomes. We acknowledge that there is important nuance when considering immune and pharmacological interactions between SCLC and TB treatment. Previous (or present) Mtb infection substantially increases the chance of development of lung cancer (Fang et al. 2025), yet immune checkpoint inhibitor treatment of lung cancer has been associated with reactivation of latent Mtb infection (Anastasopoulou et al. 2019). Still, given the prevalence of TB relapse post-antibiotic treatment (Goletti et al. 2018), it is plausible that many relapse-free TB patients could harbor granulomas after treatment. These granulomas may continue to reshape the lung immune environment via upregulation of local or systemically primed immunosuppressive lymphocytes, possibly resulting in increased Treg involvement during early tumor development.

Interestingly, we noted that in our analysis of *TumorSim*, there was no significant correlation between initial levels of macrophages or T cells and tumor outcomes, suggesting that long-term outcomes are dependent mostly on recruiting effects, which is consistent with development of adaptive immunity. This may not be a true representation as 1) we are only simulating for 14 days and 2) tissue resident memory cells are known to be present in lungs and their role in the elimination phase of SCLC tumor development may contribute to variation in outcomes of people with SCLC or developing lung lesions (Vesely et al. 2011). Advancements in single-cell technology in exploring these early immune interactions may soon allow for more detailed simulations closer to the moment of malignant transformation (Chen et al. 2021).

There are also significant differences between SCLC and TB. SCLC is associated with poor outcomes when more cancer cells proliferate (Ishibashi et al. 2017), which is recapitulated in *TumorSim*. By contrast, Mtb has evolved to replicate more slowly than other bacterial lineages (Menendez et al. 2002; Krupinsky et al. 2025). As expected, this difference is apparent in our PRCC results, given that bacterial replication rate is not a substantial driver of long-term granuloma volume (Fig. 4). Further, the parameters driving *TumorSim* reflect the primary challenge in SCLC clearance is recruitment and engagement of immune cells: most significant PRCC parameters of *TumorSim* focus on recruitment of leukocytes, rather than in-situ activation of those cells. By contrast, many PRCC-significant *GranSim* parameters involve local activation of leukocytes, including cellular sensitivity to TNF-driven apoptosis and the effectiveness of IL-10 signaling. Finally, differences in characteristic timescales means that there are no temporally direct comparisons of driving mechanisms.

*TumorSim* is a first-generation step toward building a comprehensive model of lung cancer lesions. Though there is a rich and complex body of literature exploring many cell types and cytokines associated with SCLC, it is necessary to simplify the biology for computational tractability. We have represented tumors in 2-D and assume radial symmetry to generate our predictions. While our previous work has shown that many immune mechanisms present in granulomas are well-represented by a 2-D model (Marino et al. 2018; Renardy et al. 2019), this representation may limit some investigations including 3-D tumor shape as well as how immune cells move, find each other, and follow chemokine gradients in actual 3D tissues. Careful consideration would be required to use *TumorSim* to study metastatic processes such as interaction with basement membranes. Studying *TumorSim* in 3-D is an important future direction. Some biologically-distinct cell types have very similar cell–cell interactions at *TumorSim*’s level of biological resolution, e.g., myeloid-derived suppressor cells (MDSCs) and M2 macrophages both are recruited anti-inflammatory cells (Li et al. 2022). The actions of neutrophils and natural killer cells have been included in the action of M1 macrophages and CTLs, respectively. Some cell types are present only in small numbers (e.g., B cells) (Whiteside 2008). Further, some cells are more structural by nature (e.g., fibroblasts (Whiteside 2008)), and are implicitly represented as part of the *TumorSim* grid as background lung tissue. Similarly, several continuous agents have been omitted for similar reasons, e.g., chemokines associated with omitted cell types. Finally, necrosis is a typical feature of SCLC, but is not always found in small samples comparable to those we simulate (Travis 2012). Future versions will explicitly represent these factors and others that are emerging as relevant in SCLC.

The current version of *TumorSim* is exploratory in nature. Many parameter ranges presented in Tables 2–8 have not been calibrated to experimental datasets. In this work, our simulations utilize LHS sampling to generate a stratified sample of parameter ranges allowing for errors in estimating. The sensitivity analyses show the broad impacts of each of the mechanisms on tumor outcomes. In the future, we will integrate more datasets using calibration methods such as CaliPro and Approximate Bayesian Computing with Sequential Monte Carlo sampling to refine these ranges (Joslyn et al. 2021; Nanda et al. 2023). Calibration datasets are surprisingly sparse, as many rates of cellular functions are modified within tumor microenvironments. While the cancer literature is vast, we have found that many cell-scale measurements that could be used to calibrate models like *TumorSim* have been measured in one kind of cancer but not others and synthesizing a patchwork collection of datasets must be performed carefully. This is left to do with our new collaborations with experimentalists on lung cancers.

The modular structure of our hybrid ABM *TumorSim* will allow us to integrate new biological mechanisms to create more detailed simulations. Further development will allow this platform to be the basis for hypothesis testing and exploring effects of treatment with chemotherapies and host-directed therapies. Detailed simulation of PD-1 blockade will allow us to investigate spatiotemporal effects and cell–cell interactions that underpin heterogeneous treatment responses that clinically define hot versus cold tumors; we may also investigate how this correlates with immune-infiltration based notions of tumor ‘heat’ (Galon et al. 2019). *TumorSim* may provide valuable insights for treatment of SCLC or other immunologically-similar carcinomas (Sen et al. 2025) which are the subject of our current work.

## Supplementary Information

Below is the link to the electronic supplementary material.

Supplementary file1 (PDF 600 kb)

Supplementary file2 (PDF 8056 kb)

Supplementary file3 (MOV 62111 kb)

Supplementary file4 (MOV 64998 kb)

Supplementary file5 (MOV 57443 kb)

## Data Availability

All data generated or analyzed during this study are included in this published article and are available upon request to the corresponding authors. Model details for generating simulations in this paper are available in Appendix A and in our on-line supplements. For *TumorSim*, additional information is located at http://malthus.micro.med.umich.edu/TumorSim/ and as an Online Resource. For *GranSim*, additional details and parameter values are available at http://malthus.micro.med.umich.edu/GranSim/docs/gransimrules-v2.pdf.
