# Supplementary material for: A Comparison of Mechanisms Driving Lesion Outcomes During Lung Tumor and Tuberculosis Granuloma Formation
Source: Bull Math Biol. 2026 Oct 6;88(11):197. doi: 10.1007/s11538-026-01763-8 (PMC13642502; doi:10.1007/s11538-026-01763-8)
Supplement: Supplementary file 1 [file 11538_2026_1763_MOESM1_ESM.pdf]

Appendix A. ODD-Formatted model description of *TumorSim*

This appendix describes the computational implementation of *TumorSim* using the ODD protocol (Overview, Design concepts, and Details). As presented by Grimm et al. (doi: 10.18564/jasss.4259), ODDs are a structured description of Agent-Based Model (ABM) formulation and development. We apply those principles to explain the details of *TumorSim*.

Using the ODD method we will carefully describe the implementation of each the five pillars that form all agent-based models:

1. The simulation grid, which defines the spatial domain where the tumor will develop;
2. Agents, which are the computational entities representing cells, cytokines, etc.;
3. Rules, which define how agents interact with one another;
4. Time scales, which define the frequency of agents interacting within the grid; and
5. Parameters, which are numerical characterization of the points above.

Our descriptions contain relevant pseudocode in sufficient detail that the model could be reimplemented. Biological motivation, parameter sources, and citations are given in the main text; this appendix focuses on what the program does. Notation defined in §2.1 is used throughout; numerical values appear once in §2.2 (scales) and §6 (parameters) and are referenced symbolically elsewhere.

1. Purpose and Patterns

Our ABM that we call *TumorSim* is a two-dimensional, grid-based (i.e., on-lattice), hybrid agent-based model that describes the formation of a lung tumor. Discrete cellular agents act on a slow cellular timestep; continuous agents such as soluble molecular fields are advanced on a faster diffusion timestep nested inside each cellular step; oxygen is recomputed periodically as a steady-state field, from which a relative HIF1α field is inferred.

1.1 Purpose

TumorSim simulates early small cell lung cancer (SCLC) lesion development on a local section of lung tissue. It investigates how local interactions among cancer cells, cytotoxic and regulatory T cells, M1 and M2 macrophages, soluble mediators, oxygen availability, and hypoxia-associated HIF1α signaling produce either tumor control or progressive growth. The model represents a small established tumor after malignant transformation and after immune recruitment has begun; the initial elimination phase of immunoediting is outside the scope of the current formulation of *TumorSim*.

In this study, *TumorSim* is used to:

1. Represent expansion of a small SCLC lesion on a lung-tissue grid over an early-lesion timescale;
2. Explore how anti-tumor and immunosuppressive mechanisms jointly shape lesion progression;
3. Characterize how chemokine signaling (CCL2, CCL5, CXCL9) influences immune recruitment and localization;
4. Probe immune-escape mechanisms relevant to SCLC, including Treg-mediated suppression, M2 macrophage activity, hypoxia-associated HIF1α effects, and PD-1/PD-L1-mediated suppression;
5. Provide a computational counterpart to the *GranSim* tuberculosis-granuloma model, enabling like-for-like comparison of lesion-forming processes across cancer and infection.

1.2 Patterns

The following qualitative patterns serve as evaluation criteria for whether the model behaves plausibly for its purpose. They are qualitative because several parameters in the present configuration are exploratory, and strict quantitative calibration is not the present aim.

1. **Lesion expansion from a small focus.** A small initial cancer-cell cluster can expand over the 14-day simulation when anti-tumor immune control is insufficient.
2. **Outcome depends on cytotoxic/suppressive balance.** Effective CTL and M1 macrophage activity reduces tumor burden; Treg activity, M2 polarization, hypoxia-associated suppression, and PD-1/PD-L1 signaling promote immune escape.

3. **Chemokine signaling shapes immune localization.** Altering chemokine production rates alters immune-cell abundance and spatial distribution within or near the lesion.
4. **Hypoxia emerges from local oxygen demand.** Increasing cell density produces local oxygen depletion and elevated HIF1α, which feeds back onto suppression and vascular function.
5. **PD-1/PD-L1 dynamics modulate cytotoxicity.** PD-1 and PD-L1 expression emerge dynamically from local conditions and reduce effective CTL killing when both are present.

1.3 Code availability

The *TumorSim* source code is not available for distribution due to intellectual-property constraints. This appendix is the primary reference for the algorithmic specification, and is intended to provide a description detailed enough to reconstruct the algorithm independently. Parameter values are reported in §6 with Non-symbolic names matching the implementation; this enables independent reimplementation and provides the configuration record for the simulations reported in the main text.

2. Entities, State Variables, and Scales

The following subsections define the entities and state variables associated with *TumorSim*, the computational environment in which they exist, and defines our notion of "scales". This section essentially "declares" these model components, and how they are connected follows in subsequent sections.

2.1 Notation and Conventions

The following notation is used throughout this appendix; defining it here lets later sections remain concise.

Timescales and intervals

Symbol	Value	Meaning
Δt_s	10 min	Cellular timestep (slow scale)
Δt_m	4 s	Molecular diffusion substep (fast scale)
N_{diff}	150	Diffusion substeps per cellular step (= $\Delta t_s / \Delta t_m$)
T_{O_2}	144 Δt_s = 24 h	O_2 and HIF1α recompute interval
T_{shuffle}	6 Δt_s = 1 h	Agent-list shuffle interval

Spatial scale

Symbol	Value	Meaning
Δx	20 µm	Grid-compartment edge length
L	6 mm	Domain edge length
N_{grid}	300 × 300	Number of grid compartments
$N_8(x)$	-	Moore neighborhood of grid position x: x and its eight surrounding compartments

Functional forms. Many *TumorSim* rules use saturating concentration responses. To avoid restating these forms repeatedly, we adopt:

- Activating Hill-1 (Michaelis–Menten): $H_+(C; V, K) = \dfrac{VC}{C+K}$
- Inhibitory Hill-1: $H_-(C; V, K) = \dfrac{VK}{C+K} = V - H_+(C; V, K)$
- Hill-h with explicit exponent: $H_+^h(C; V, K, h) = \dfrac{VC^h}{C^h + K^h}$

  In each, C is the concentration, V is the supremum value of H, the half-saturation value K is defined such that $H(K; V, K) = V/2$. In some cases, we adjust the steepness of the hill curve by increasing h.

When two chemokines contribute additively to a single recruitment or sensing term, the corresponding rule is written as a sum of H_+ terms.

Stochastic event resolution. Every probabilistic event in *TumorSim* is resolved as an independent Bernoulli draw against the relevant probability at the moment the event is evaluated. When an agent evaluates multiple potential events in one step (e.g., a CTL evaluating activation, PD-1 conversion, killing, and proliferation), each is drawn independently in the order specified by the submodel.

2.2 Spatial and Temporal Scales

The domain is a square grid of N_{grid} = 300 × 300 compartments, each of edge length Δx = 20 μm, representing an $L \times L$ = 6 mm $\times$ 6 mm region of lung tissue. Periodic boundary conditions apply to neighborhood movement and to continuous-field calculations. The simulation duration in the present study is 14 days, an early-lesion timescale chosen because progressive virtual SCLC tumors can exceed the domain after roughly two weeks.

A grid compartment can hold at most one cellular or vascular source agent. If a motile cell attempts to move into an occupied compartment, the move fails and the cell remains at its current location. Vascular sources are immovable.

The two-timescale separation (Δt_s vs Δt_m, with N_{diff} substeps nested inside each cellular step) places soluble-mediator dynamics on the fast scale where diffusion is well-resolved, while keeping cellular decisions, recruitment, and growth on the slower 10-minute scale. Oxygen is treated differently: rather than being advanced through repeated diffusion steps, the O_2 field is recomputed as a steady state every T_{O_2} = 24 h (§7.2).

2.3 Discrete Agents

TumorSim contains five classes of discrete agents entity:

1. cancer cells;
2. cytotoxic T lymphocytes (CTLs);
3. regulatory T cells (Tregs);
4. macrophages (M1 or M2 phenotype);
5. vascular sources.

Entity	Biological interpretation	Motile?	Cell-type-specific state	Main processes
Cancer cell	SCLC tumor cell	No active migration	PD-L1 status; alive/dead/degraded; death time; degradation timer	Proliferation; PD-L1 induction; CTL activation by contact; CTL deactivation through PD-L1; death by CTL or M1; degradation and removal
CTL	Cytotoxic T lymphocyte	Yes	Activation state; PD-1 status	Chemotaxis (CCL5, CXCL9); activation; PD-1 conversion; cancer-cell killing; IFN-γ secretion; proliferation
Treg	Regulatory T cell	Yes	—	Chemotaxis (CCL5); CTL deactivation; proliferation
Macrophage	M1 or M2 macrophage	Yes (rate-limited)	M1/M2 phenotype	Chemotaxis (CCL2, CCL5); M1 cancer-cell killing; M1 CTL activation; M2 CTL deactivation; M1↔M2 switching; cytokine/chemokine secretion; proliferation
Vascular source	Local vascular access	No	Source state; local blockage	Oxygen contribution; immune recruitment; HIF1α-dependent blockage update

Common state variables

All discrete agents share the common state variables `id`, `pos` (i.e., position within the grid), `birthTime`, `doesProliferate` (and, if true, `doublingTime`), `state`, `nextState`, and `inTumor`, with the use of `state` / `nextState` governed by the hybrid update rule below.

Hybrid state update. *TumorSim* uses a hybrid update scheme that is referenced repeatedly:

- *Self-transitions* (e.g., PD-1±/PD-L1± conversion, M1↔M2 polarization, proliferation eligibility) are computed into a `nextState` field during the next-state pass and committed after the pass completes.
- *Cross-agent interactions* (CTL activation/deactivation by a neighbor; CTL deactivation by a PD-L1+ cancer cell or M2 macrophage) write immediately to both `state` and `nextState` of the target agent during the acting agent's pass. They therefore take effect *within* the same timestep and influence subsequent next-state computations.
  Combined with the fixed agent-type processing order (cancer → CTL → Treg → macrophage → vascular source) and periodic within-type shuffling, this scheme determines exactly when each interaction becomes visible to other agents.

In-tumor classification. Each discrete agent is classified as in-tumor or not in-tumor. Cancer cells are always considered in-tumor. An immune cell is classified as in-tumor if a Moore-direction search of radius `maxMooreRadiusInTumor` (3 compartments; §6.1) detects surrounding cancer-cell structure. The classification is used for summary statistics and the in-tumor placement branch of the growth/shrinkage algorithm (§7.5).

2.4 Continuous Agents

In addition to discrete agents, *TumorSim* contains several continuous agents representing chemokines. Continuous agents are defined on the same grid as the discrete agents, with one scalar value per grid compartment.

Field	Units / range	Produced by	Loss	Role
IFN-γ	molecules / compartment	CTLs, M1 macrophages	Diffusion, degradation	Activates CTLs; M2→M1 polarization; PD-1^+→PD-1^- conversion; CXCL9 secretion by M1
CCL2	molecules / compartment	Cancer cells	Diffusion, degradation	Macrophage recruitment and chemotaxis
CCL5	molecules / compartment	Cancer cells	Diffusion, degradation	CTL, Treg, macrophage recruitment and chemotaxis
CXCL9	molecules / compartment	M1 macrophages (IFN-γ-induced)	Diffusion, degradation	CTL recruitment and chemotaxis
O_2	molecules / compartment	Vascular sources; lung-tissue background	Cellular consumption	Drives hypoxia (HIF1α)
HIF1α	unitless, 0–1	Derived from O_2	Recomputed from O_2	PD-1/PD-L1 induction; M1→M2 polarization; CTL IFN-γ inhibition; vascular blockage

The four diffusing fields are advanced on the fast Δt_m timestep; O_2 is recomputed as a steady-state field every T_{O_2}; HIF1α is derived pointwise from O_2 on the same schedule.

2.5 Cell-State Variables

Tables in this subsection list only the state variables specific to each cell type, in addition to the common variables given in §2.3. Biological motivation is in main text §2.1.

Cancer cell.

Variable	Description
PD-L1 status	PD-$L1^-$ initially; can be induced PD-$L1^+$ via HIF1α or CTL contact
Alive/dead/degraded state	Tracks viability; dead cells persist for a degradation interval (§6.5) before removal
Death time	Timestep at which the cell was killed

Cancer-cell positions change only indirectly during growth/shrinkage rearrangements (§7.5). Only cancer cells die and degrade in the present configuration; CTLs, Tregs, and macrophages do not die over the duration of the simulation.

CTL.

Variable	Description
Activation state	Active or inactive
PD-1 status	PD-1$^-$ initially; HIF1α can induce PD-1$^+$; IFN-γ can revert CTLs to PD-1$^-$

Treg. No cell-type-specific state beyond the common variables.

Macrophage.

Variable	Description
Phenotype	M1 or M2; switches under local IFN-γ and HIF1α

Vascular source.

Variable	Description
Source state	Discrete status used for recruitment and updates
Blockage	HIF1α-derived; modulates passage of O_2 and recruited cells

3. Process Overview and Scheduling

TumorSim employs a partial-asynchronous schedule. Partial asynchronous here means that we process the actions and interactions of each cell in the simulation by iterating over lists of cells organized by type in the following order (1. Cancer Cells, 2. CTLs, 3. Tregs, 4. Macrophages, 5. Vascular Sources). That is, we first iterate over all of the cancer cells in a shuffled order, then over the CTLs in a shuffled order, and so on.

The partial-asynchronous schedule has three nested cadences: an inner molecular loop running N_{diff} = 150 substeps per cellular step, a periodic steady-state O_2 recomputation every T_{O_2} = 24 h, and the slow cellular cadence Δt_s = 10 min on which movement, recruitment, state transitions, and growth/shrinkage occur.

3.1 One Cellular Timestep (visual overview)

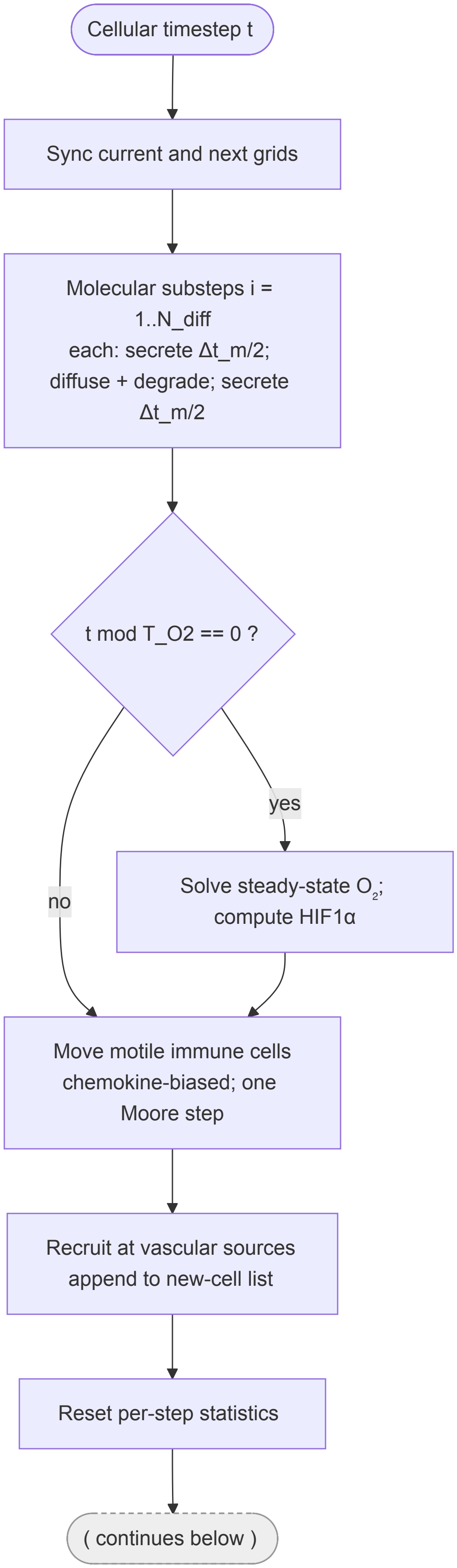
Cellular timestep t
Sync current and next grids
Molecular substeps i = 1..N_diff each: secrete Δt_m/2; diffuse + degrade; secrete Δt_m/2
t mod T_O2 == 0 ?
yes
no
Solve steady-state O_2; compute HIF1α
Move motile immune cells chemokine-biased; one Moore step
Recruit at vascular sources append to new-cell list
Reset per-step statistics
(continues below)

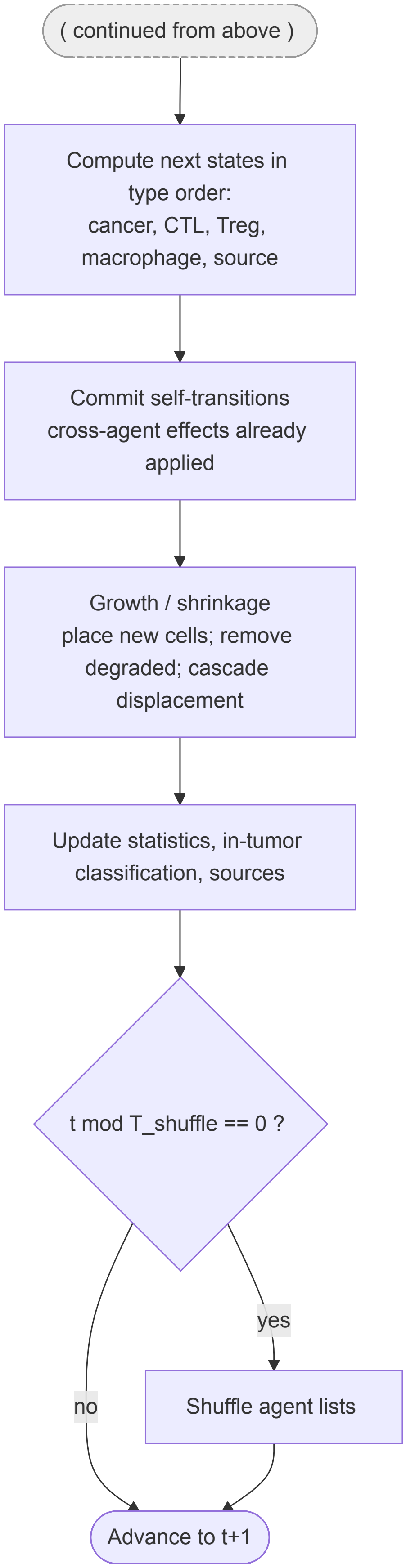

3.2 Pseudocode for one cellular timestep

```
for each cellular timestep t:
   sync(currentGrid, nextGrid)
   // --- Molecular fields on fast timescale ---
```

```
    for i = 1..N_diff:
        secreteAll(Δt_m / 2)                    // IFN-γ, CCL2, CCL5, CXCL9
        diffuseAndDegrade(Δt_m)                 // ADE scheme; periodic BCs
        secreteAll(Δt_m / 2)
    // --- Periodic oxygen and HIF1α ---
    if t mod T_O2 == 0:
        u ← solve( (D_O2/Δx²) A − λ I ) u = f   // iterative sparse solver
        HIF1α ← 1 − H₊ʰ(O₂; 1, b_O2, h_O2)
    // --- Cellular movement (motile immune cells only) ---
    for each motile immune cell c (in current list order):
        if c is macrophage and (t − c.lastMove) < movementInterval(c.phenotype):
            continue
        evaluate chemokine weights on N₈(c.pos)
        sample destination from categorical weights (or uniform if no signal)
        if destination is unoccupied: move; else: stay
    // --- Recruitment ---
    for each vascular source s:
        for each recruitable cell type τ:
            p_rec ← H₊-summed recruitment rule for τ at s.pos (§7.4)
            with prob p_rec: append new cell of type τ to newCellList
    resetPerStepStats()
    // --- Next-state computation in fixed type order ---
    for type in (cancer, CTL, Treg, macrophage, source):
        for each agent a in currentList\[type\]:     // within-type order may be shuffled
            computeNextState(a)                      // cross-agent effects on neighbors are
                                                     // applied immediately to state and nextState
    // --- Commit self-transitions ---
    for each agent a: a.state ← a.nextState
    // --- Tumor growth / shrinkage ---
    // See **Phases of the growth/shrinkage step.** in §7.5 below for details.
    growTumor:
        for each daughter cell
           add it to the grid, via  a cell cascade from the parent's cell position to the nearest tumor
boundary position
    shrinkTumor:
        for each fully degraded cancer cell
            remove the degraded cell and move tumor cells from the farthest tumor boundary position to the
position of the fully degraded cell
    updateAgentStats()
    updateInTumorStatus()
    updateVascularSources()                            // source-state and blockage from HIF1α
    updateGlobalStats()
    if t mod T_shuffle == 0:
        for each list ∈ {cancer, CTL, Treg, macrophage, source}: shuffle(list)
```

3.3 Annotations on selected steps

Strang splitting of the molecular substep. Secretion is applied for $\Delta t_m/2$ on each side of the diffuse-and-degrade step, so that secretion source terms are second-order accurate with respect to the diffusion timescale. The diffusion routine uses an Alternating Direction Explicit (ADE) scheme with separate intermediate arrays for the two sweep directions, followed by averaging and exponential-decay degradation within each substep (i.e., field values are multiplied by exp(−k_deg · Δt_m) rather than using a linear Euler approximation).

For details on our numerical methods, please see *Cilfone et al. 2014*, "Strategies for Efficient Numerical Implementation of Hybrid Multi-scale Agent-Based Models to Describe Biological Systems", *Cellular and Molecular Bioengineering* (doi: 10.1007/s12195-014-0363-6).

Fixed agent-type order. Cancer cells are processed first because their PD-L1 induction and CTL-deactivation effects on neighboring CTLs must be visible before CTLs evaluate killing. CTLs are processed before Tregs and macrophages so that downstream Treg- and M2-mediated CTL deactivation acts on the latest CTL state.

Hybrid update rule. As stated in §2.3, self-transitions are committed after the full pass; cross-agent activation/deactivation effects propagate immediately. This was a deliberate choice: deferring all cross-agent effects until the end of the pass would prevent within-step propagation of activation cascades (e.g., M1 activating a neighbor CTL that then kills a neighbor cancer cell in the same step), which we view as biologically appropriate at the 10-minute timescale.

Movement rate-limiting. CTLs and Tregs attempt movement every Δt_s. M1 macrophages move once per 23 Δt_s ($\approx$ 3.8 h), M2 macrophages once per 6 Δt_s ($\approx$ 1 h), reflecting slower in-tissue dwell behavior.

Shuffling. Agent-list shuffling every T_{shuffle} = 1 h randomizes within-type processing order without altering positions, states, or grid occupancy. The type-level order remains fixed.

3.4 Reproducibility note

The schedule above is part of the model definition. Reimplementations must preserve: N_{diff} substeps per cellular step; the placement of movement before recruitment and next-state computation; the fixed type order during next-state computation; the hybrid self-versus-cross-agent update; the delayed placement and removal of cells during growth/shrinkage; and periodic list shuffling at T_{shuffle}.

4. Design Concepts

TumorSim is a mechanistic, rule-based, grid-based ABM. Tumor-level behavior is not prescribed; lesion growth, infiltration, hypoxia, and immune escape emerge from local interactions among agents and continuous fields.
The subsections that follow describe *TumorSim* as they relate to common design principles employed during ABM studies, and are suggested by the ODD protocol.

4.1 Basic principles

The model rests on six principles: (i) cancer-cell proliferation drives expansion; (ii) cytotoxic immune activity (active CTLs, M1 macrophages) can oppose growth; (iii) immunosuppression (Treg- and M2-mediated CTL deactivation, PD-1/PD-L1, HIF1α effects, vascular blockage) can impair cytotoxic control; (iv) chemokines (CCL2, CCL5, CXCL9) regulate immune recruitment and movement; (v) oxygen limitation produces HIF1α-mediated feedback onto suppression and vasculature; (vi) spatial structure matters — local occupancy, Moore-neighborhood interactions, chemotaxis, diffusion, and heterogeneous oxygen distributions are explicit. The model deliberately omits the elimination phase of immunoediting, antigen presentation, lymph-node priming, $CD4^+$ and γδ T cells, NK cells, dendritic cells, fibroblasts, detailed vasculature, and 3D architecture; these are outside the scope.

4.2 Emergence

Tumor growth or control, immune infiltration patterns, hypoxic-region formation, HIF1α-driven suppression, and vascular impairment all emerge from local rules rather than being imposed. Emergent outputs include cancer-cell number, tumor area, immune-subset counts, $PD\text{-}1^+$ and $PD\text{-}L1^+$ fractions, M1/M2 balance, molecular field distributions, oxygen and HIF1α maps, and overall growth trajectory. As an illustrative chain: local oxygen consumption produces hypoxia → elevated HIF1α → increased PD-1/PD-L1 expression and M1→M2 conversion → reduced cytotoxic effectiveness → larger lesion → further hypoxia.

4.3 Adaptation

Agents do not optimize a fitness function. They change state and behavior in response to local conditions via probabilistic rules: CTL activation responds to IFN-γ and cancer-cell contact; PD-1 status responds to HIF1α and IFN-γ; PD-L1 induction responds to HIF1α and CTL contact; CTL killing efficacy depends on PD-1/PD-L1 states; macrophage polarization responds to HIF1α (M1→M2) and IFN-γ (M2→M1); chemotaxis biases movement by local chemokine fields; vascular blockage tracks local HIF1α. Adaptation in the ODD sense — condition-dependent state — is present; strategic decision-making is not.

4.4 Objectives and Learning

TumorSim has neither objectives nor learning. Agents do not maximize utility, plan, or modify rules based on experience. State variables retain only the information explicitly listed in §2: birth time, proliferation timing, current phenotype/activation, and (for

cancer cells) death time. Long-term immune memory and antigen-specific clonal selection are out of scope for the 14-day early-lesion focus.

4.5 Prediction

Agent decisions use current local information only: local field concentrations, local HIF1α, Moore-neighborhood occupants, the agent's current state, proliferation timing, and grid occupancy. No agent anticipates future conditions.

4.6 Sensing

Sensing is local. *Neighborhood sensing* operates over N_8 and underlies all direct cell–cell interactions (CTL and M1 killing; Treg- and M2-mediated deactivation; M1-mediated and contact-mediated CTL activation; CTL induction of PD-L1; PD-L1$^+$ deactivation of PD-1$^+$ CTLs). *Field sensing* reads continuous-field values at the agent's compartment: IFN-γ (CTL activation; PD-1$^+$→PD-1$^-$; M2→M1; CXCL9 induction in M1), HIF1α (CTL PD-1 induction; cancer-cell PD-L1 induction; M1→M2; inhibition of CTL IFN-γ; vascular blockage), and the chemokines (CCL2 for macrophages; CCL5 for CTLs, Tregs, and macrophages; CXCL9 for CTLs). *Chemotactic sensing* reads chemokine values in N_8 when the field at the agent's position lies within the configured chemotactic sensitivity range C_min < C < C_max (§6.1).

4.7 Interaction

Four interaction modes operate. *Direct contact*, where the cross-agent interactions listed under sensing, is applied immediately under the hybrid update rule (§2.3). *Field-mediated* interaction occurs through secretion, diffusion, and degradation of soluble fields. *Recruitment-mediated* interaction: cancer-cell and M1 secretion shape chemokine fields near vascular sources and thereby influence which immune subsets are recruited. *Space-mediated* interaction arises from the one-cell-per-compartment rule, which creates competition for grid space during movement, proliferation, recruitment, and growth/shrinkage.

4.8 Stochasticity

Initial placement of immune cells and vascular sources is stochastic subject to occupancy. Proliferative-fraction assignment is stochastic. Movement destinations are sampled from categorical chemokine weights or uniformly when no signal is present. Recruitment is Bernoulli with chemokine-dependent probability. All cell–cell killing, deactivation, activation, PD-1/PD-L1 and M1/M2 conversions, and proliferation events are Bernoulli draws (§2.1). Cell-list ordering is shuffled at T_{shuffle}. Removed-agent processing order during shrinkage is randomized.

4.9 Collectives

TumorSim does not define a formal tumor agent. The tumor is an emergent spatial collective: cancer cells are always in-tumor; immune cells are classified by the in-tumor rule (§2.3); the tumor boundary is determined operationally each timestep as those occupied compartments with at least one unoccupied neighbor.

4.10 Observation

Observation of the ABM state occurs after state updating, growth/shrinkage, in-tumor classification, and source updates within each cellular step, so recorded statistics reflect the completed system state at that timestep. Recorded outputs include cell counts (total, by type, by activation/phenotype state), tumor size, area, and growth trajectory; molecular and environmental fields (IFN-γ, CCL2, CCL5, CXCL9, O_2, HIF1α, blockage); spatial agent positions; and derived metrics (CTL:Treg ratio, M1:M2 ratio, active/PD-1$^+$/PD-L1$^+$ fractions, infiltration measures, hypoxic fraction).

Volume inference from 2D area. *TumorSim* is two-dimensional but tumor volume is reported for comparison with 3D clinical observations. The procedure is: count tumor-associated cells; convert to 2D area via Δx^2; approximate the tumor as a disc; compute equivalent radius; assume a spherical tumor with that radius. This scaling is acknowledged as a simplification (main text §2). The 2D categorical trajectory classification (progressing, dormant, regressing) is based on the time series of inferred volume.

5. Initialization

TumorSim is initialized to represent an early SCLC lesion several days after malignant transformation and after initial immune recruitment has begun, rather than the transformation event or the full elimination phase.

5.1 Spatial Domain

The spatial domain of the simulation is the N_{grid} grid defined in §2.2. Each compartment is initially empty except where filled by the steps below.
As an overview, the agents are placed in the following order:

- Vascular sources (§5.2)
- Immune cells (§5.3)
  - CTL cells
  - Treg cells
  - M1 Macrophages
  - M2 Macrophages
- Cancer cells (§5.4)
  All cytokines begin at concentration 0, but come to quasi-steady-state shortly after the simulation starts due to cellular secretion.

5.2 Initial vascular sources

Vascular sources are placed stochastically at the configured density `Core.Source.sourceDensity` (§6.9), subject to a border exclusion `Core.Source.sourceBorderSize`. They are initialized as active, immobile, with blockage computed from local HIF1α after the initial O_2 steady-state solve.

5.3 Initial immune-cell populations

CTL, Treg, M1 macrophage, and M2 macrophage populations, in this order, are stochastically distributed on the grid at their respective initial densities (§6.6–6.8), subject to one-cell-per-compartment occupancy. Placement re-tries an occupied compartment until a free one is found. The program will terminate if no free compartment is available, as this means that the user has specified too many agents for the specified grid size. CTLs are initialized PD-1$^-$; PD-1$^+$ CTLs emerge dynamically via HIF1α. Activation state, phenotype, proliferative status, and doubling-time interval are assigned from the parameters in §6.8–6.10. In-tumor status is computed from the §2.1 rule after placement.

5.4 Placement of cancer cells onto the grid creating the initial tumor

A circular cluster of cancer cells of diameter 10 cells ($\approx$200 μm) is placed at the grid center. Each cancer cell is initialized as alive, PD-L1$^-$, proliferative status assigned from the cancer-cell proliferating fraction, and a doubling-time interval set to the cancer-cell parameter. PD-L1$^+$ cells emerge dynamically during simulation through HIF1α-driven conversion or CTL-induced expression (§7.6). The birthTime is random, so the cancer cells don't all proliferate on the same time step. The `birthTime` of each cancer cell is initialized to a random number in the interval (-doublTimeTimeInterval, 0] so that it will proliferate in time interval (0, doublingTimeInterval].

5.5 Initial molecular and environmental fields

IFN-γ, CCL2, CCL5, and CXCL9 fields are initialized to zero across the grid. The O_2 field is computed by the steady-state solve (§7.2) using the initial agent configuration; HIF1α is then derived pointwise from O_2. Subsequent updates follow the schedule in §3.

5.6 Stochastic seeding

Each simulation, including replicate simulations using the same parameter values, uses an independent random seed. Across replicates with the same parameter set, initial cell positions and proliferative-status assignments differ; this produces aleatory variability across replicates that is reported in main text Figure 2.

6. Input Data

All *TumorSim* inputs are static configuration parameters specified in an XML file at simulation start. Biological rationale and literature citations for these parameters are given in the main text (Tables 2–8); this section lists Non-symbolic names, representative values, and units for the configuration used to generate the simulations reported in the main text. Values shown are exemplars from the calibrated/exploratory ranges; for the full ranges varied across the 500 LHS samples described in main text §2.3, see main text Tables 2–8.
TumorSim does not consume time-varying external inputs (e.g., empirical signals or driving forcings).

In the following tables, single values have been reported - these are our stable "baseline" values for a reasonable stable simulation. The ranges used in the main text have been reported in the main text as Tables 2-8.

6.1 Scheduler and core parameters

These "Core parameters" govern the main underlying architecture of *TumorSim*.

Non-symbolic name	Value	Meaning
`Core.timeStepSteadyStateO2`	144 Δt_s	T_{O_2} (see §2.1)
`Core.diffusivityChemokines`	5.2×10^{-8} cm^2/s	Shared diffusion coefficient for CCL2, CCL5, CXCL9
`Core.ChemokinekDeg`	1.58×10^{-3} s^{-1}	Chemokine degradation rate
`Core.minChemotaxis`	0.458 molecules	C_min for chemotactic sensitivity
`Core.maxChemotaxis`	435.77 molecules	C_max for chemotactic sensitivity
`Core.maxMooreRadiusInTumor`	3 compartments	Radius for in-tumor classification

Note that the diffusion-substep parameter `Core.timeStepSteadyStateO2` must evenly divide N_{diff}, so that an even integer number of substeps are performed per diffusion step. This number must be even due to the implementation of Strang splitting (§7.1).

6.2 Oxygen

The following parameters describe oxygen (`O2`) in the simulation. Oxygen is solved as a steady state over the full grid (§7.2) from a forcing vector that combines vascular input, background lung-tissue contribution, and cellular consumption.

Non-symbolic name	Value	Units	Role
`O2.diffusivityO2`	3.08×10^{-5}	cm^2/s	Diffusivity of oxygen
`O2.sourceO2Effect`	-1.7×10^{11}	molecules/s	Vascular source contribution (sign per linear-system convention)
`O2.m1O2Effect`	3.85×10^{7}	molecules/s	M1 consumption
`O2.m2O2Effect`	6.7×10^{7}	molecules/s	M2 consumption
`O2.CTLO2Effect`	2.4×10^{6}	molecules/s	CTL consumption
`O2.tregO2Effect`	2.4×10^{6}	molecules/s	Treg consumption
`O2.cancerCellO2Effect`	6.7×10^{7}	molecules/s	Cancer-cell consumption
`O2.o2BoundaryValue`	9.2×10^{8}	molecules/s	Background per-compartment input
`O2.lambda`	0.3	—	λ in the steady-state equation

6.3 HIF1α and vascular blockage

The following parameters describe HIF1α distribution throughout the grid. HIF1α is unitless and ranges from 0 (well-oxygenated) to 1 (completely hypoxic).

Non-symbolic name	Value	Units	Role in H_+^h
`HIF1a.bO2`	34.36	mg/L	K in HIF1α-from-O_2 conversion

Non-symbolic name	Value	Units	Role in H_+^h
`HIF1a.hO2`	3.8	—	h in HIF1α-from-O_2 conversion
`HIF1a.bSourceBlockage`	0.267	—	K in blockage-from-HIF1α conversion
`HIF1a.hSourceBlockage`	4.8	—	h in blockage-from-HIF1α conversion

6.4 IFN-γ

The following parameters describe secretion and diffusion of IFN-γ, which is a continuous agent diffusing throughout the grid.

Non-symbolic name	Value	Units	Role
`IFNg.vmaxCTL`	0.328	molecules/s	V in H_- for CTL IFN-γ secretion
`IFNg.c50CTL`	0.573	HIF1α units	K in H_- for CTL IFN-γ secretion
`IFNg.m1MacSecretionRate`	2.63×10^{-3}	molecules/s	Constant M1 IFN-γ rate
`IFNg.diffusivityIFNg`	1.4×10^{-8}	cm^2/s	D for IFN-γ
`IFNg.kDeg`	1.58×10^{-2}	s^{-1}	k_deg for IFN-γ

6.5 Cancer cells

The following parameters describe cancer cell behavior in *TumorSim*.

Non-symbolic name	Value	Meaning
`Core.CancerCell.percentPDL1Plus`	0	Initial PD-L1⁺ fraction
`Core.CancerCell.probPDL1PlusDeactActPD1PlusCTL`	0.0134	Probability PD-L1⁺ cell deactivates adjacent active PD-1⁺ CTL
`Core.CancerCell.probProliferating`	0.6847	Proliferating-fraction probability
`Core.CancerCell.vmaxPDL1MinusToPDL1PlusConversion`	0.4997	V in H_+ for HIF1α-driven PD-L1 induction
`Core.CancerCell.c50PDL1MinusToPDL1PlusConversion`	0.9759	K in H_+ for HIF1α-driven PD-L1 induction
`Core.CancerCell.dCCL2`	0.084 molecules/s	Cancer-cell CCL2 secretion rate
`Core.CancerCell.dCCL5`	0.046 molecules/s	Cancer-cell CCL5 secretion rate
`Core.CancerCell.dCXCL9`	0	Cancer-cell CXCL9 secretion (off in present configuration)
`Core.CancerCell.CancerCellType1.cellDegradedInterval`	360 Δt_s (60 h)	Dead-to-degraded persistence
`Core.CancerCell.CancerCellType1.doublingTimeInterval`	288 Δt_s (48 h)	Cancer-cell doubling time

6.6 CTL

The following parameters describe CTL behavior in *TumorSim*.

Non-symbolic name	Value	Meaning
`Core.CTL.activeInitDensity`	0.00070	Active CTL initial density
`Core.CTL.inactiveInitDensity`	0	Inactive CTL initial density
`Core.CTL.percentPD1Plus`	0	Initial PD-1⁺ fraction
`Core.CTL.activeDoublingTimeInterval`	48 Δt_s (8 h)	Active CTL doubling time
`Core.CTL.inactiveDoublingTimeInterval`	1080 Δt_s (7.5 d)	Inactive CTL doubling time

Non-symbolic name	Value	Meaning
`Core.CTL.probActiveProliferating`	0.05	Active CTL proliferating fraction
`Core.CTL.probInactiveProliferating`	0.01	Inactive CTL proliferating fraction
`Core.CTL.probActPD1MinusKillPDL1Plus`	0.010	Kill prob: active PD-1⁻ on PD-L1⁺ target
`Core.CTL.probActPD1PlusKillPDL1Plus`	0	Kill prob: active PD-1⁺ on PD-L1⁺ target
`Core.CTL.probActPD1MinusKillPDL1Minus`	0.422	Kill prob: active PD-1⁻ on PD-L1⁻ target
`Core.CTL.probActPD1PlusKillPDL1Minus`	0.0607	Kill prob: active PD-1⁺ on PD-L1⁻ target
`Core.CTL.probInducePDL1`	0.3745	CTL induces PD-L1 on adjacent cancer cell
`Core.CTL.probActivationByCancerCell`	0.4134	Contact-mediated activation prob
`Core.CTL.vmaxIFNgActivation`	0.1273	V in H_+ for IFN-γ-driven activation
`Core.CTL.c50IFNgActivation`	2.8931	K in H_+ for IFN-γ-driven activation
`Core.CTL.vmaxPD1MinusToPD1PlusConversion`	0.5424	V for HIF1α-driven PD-1 induction
`Core.CTL.c50PD1MinusToPD1PlusConversion`	0.9572	K for HIF1α-driven PD-1 induction
`Core.CTL.vmaxPD1PlusToPD1MinusConversion`	0.0667	V for IFN-γ-driven PD-1 loss
`Core.CTL.c50PD1PlusToPD1MinusConversion`	2.134	K for IFN-γ-driven PD-1 loss

6.7 Treg

The following parameters describe Treg behavior in *TumorSim*.

Non-symbolic name	Value	Meaning
`Core.Treg.activeInitDensity`	7.24×10^{-4}	Initial Treg density
`Core.Treg.doublingTimeInterval`	720 Δt_s (5 d)	Treg doubling time
`Core.Treg.probProliferating`	0.03	Treg proliferating fraction
`Core.Treg.probDeactCTL`	0.25	Treg deactivates adjacent CTL

6.8 Macrophage

The following parameters describe both M1 and M2 behavior in *TumorSim*.

Non-symbolic name	Value	Meaning
`Core.Mac.initDensityM1`	4.576×10^{-5}	Initial M1 density
`Core.Mac.initDensityM2`	7.557×10^{-4}	Initial M2 density
`Core.Mac.m1DoublingTimeInterval`	216 Δt_s (36 h)	M1 doubling time
`Core.Mac.m2DoublingTimeInterval`	288 Δt_s (48 h)	M2 doubling time
`Core.Mac.probProliferating`	0.079	Macrophage proliferating fraction
`Core.Mac.m1KillProb`	0.19	M1 kills adjacent cancer cell
`Core.Mac.m1ActivateCTL`	0.37	M1 activates adjacent CTL
`Core.Mac.m2CTLDeactProb`	0.8341	M2 deactivates adjacent CTL
`Core.Mac.vmaxM1M2Conversion`	0.729	V for HIF1α-driven M1→M2
`Core.Mac.c50M1M2Conversion`	0.1144	K for HIF1α-driven M1→M2
`Core.Mac.vmaxM2M1Conversion`	0.8642	V for IFN-γ-driven M2→M1
`Core.Mac.c50M2M1Conversion`	1.647	K for IFN-γ-driven M2→M1
`Core.Mac.dCCL2`	0	Macrophage CCL2 secretion (off)
`Core.Mac.dCCL5`	0	Macrophage CCL5 secretion (off)

Non-symbolic name	Value	Meaning
`Core.Mac.c50CXCL9IFNSecretion`	90	K in H_+ for IFN-γ→CXCL9 in M1
`Core.Mac.dCXCL9`	12 molecules/s	V in H_+ for IFN-γ→CXCL9 in M1
`Core.Mac.movementM1`	23 Δt_s	M1 movement interval
`Core.Mac.movementM2`	6 Δt_s	M2 movement interval

6.9 Vascular sources

The following parameters describe vascular source density during simulation initialization.

Non-symbolic name	Value	Meaning
`Core.Source.sourceDensity`	0.0144	Source density
`Core.Source.sourceBorderSize`	1 compartment	Border exclusion for placement

Note that sourceBorderSize is implemented to make sure that no sources appear directly on the edge of the simulation domain. We do this in order to avoid potential artifacts in some of the submodel argorithms described in §7.

6.10 Recruitment

Recruitment rules use summed Michaelis–Menten (H_+) terms over the relevant chemokines (§7.4). Per-cell-type parameters:

Non-symbolic name	Value	Meaning
`Recruit.Prob.Mac.vmaxM1`	0.01312	V_max for M1 recruitment
`Recruit.Prob.Mac.c50M1`	3.816	K for M1 recruitment
`Recruit.Prob.Mac.vmaxM2`	0.01312	V_max for M2 recruitment
`Recruit.Prob.Mac.c50M2`	3.816	K for M2 recruitment
`Recruit.Prob.CTL.vmaxCTL`	0.0418	V_max for CTL recruitment
`Recruit.Prob.CTL.c50CTL`	7.46	K for CTL recruitment
`Recruit.Prob.Treg.vmaxTreg`	0.0244	V_max for Treg recruitment
`Recruit.Prob.Treg.c50Treg`	1.49	K for Treg recruitment

7. Submodels

This section gives the algorithmic rules and equations for each major subprocess in *TumorSim*. Equations use the H_+/H_-/H_+^h notation from §2.1; numerical values are given in §6 and not repeated here.

7.1 Soluble molecular fields

This subsection describes diffusion of four diffusing continuous agents representing molecular concentrations other than Oxygen (which is described in §7.2). These follow a reaction–diffusion–decay PDE on the periodic domain. For each field C:

$$\frac{\partial C}{\partial t} = D\nabla^2 C - k_{\text{deg}}C + S(x,t)$$

with field-specific D and k_{deg} (§6.1, §6.4) and a source term S accumulating contributions from all secreting cells in the compartment. The grid update advances this PDE through N_{diff} substeps per cellular step using a Strang split (secrete for $\Delta t_m/2$, then diffuse-and-degrade, then secrete for $\Delta t_m/2$). The diffusion routine uses an Alternating Direction Explicit (ADE) scheme with separate intermediate arrays for the two sweep directions, followed by averaging; degradation is applied as exponential decay (factor $\exp(-k_{\text{deg}} \cdot \Delta t_m)$) within each substep rather than as a linear Euler step.

Source terms. Cancer cells secrete CCL2 and CCL5 at constant rates `Core.CancerCell.dCCL2` and `Core.CancerCell.dCCL5`. M1 macrophages secrete IFN-γ at a constant rate `IFNg.m1MacSecretionRate`. M1 macrophages secrete CXCL9 in response to local IFN-γ:

$$s_{\mathrm{CXCL9,M1}}(C_{\mathrm{IFN}\gamma}) = H_{+}\big(C_{\mathrm{IFN}\gamma};, V_{\mathrm{max,CXCL9}},, C_{50,\mathrm{CXCL9}}\big).$$

CTLs secrete IFN-γ in a manner inhibited by HIF1α:

$$s_{\mathrm{IFN}\gamma,\mathrm{CTL}}(C_{\mathrm{HIF1}\alpha}) = H_{-}\big(C_{\mathrm{HIF1}\alpha};, V_{\mathrm{max,IFN}\gamma,\mathrm{CTL}},, C_{50,\mathrm{IFN}\gamma,\mathrm{CTL}}\big).$$

In the present configuration, cancer-cell CXCL9 secretion and macrophage CCL2/CCL5 secretion are off.

7.2 Oxygen, HIF1α, and vascular blockage

Here, we describe the diffusion and accumulation and consumption of Oxygen within the grid, and the downstream impacts on accumulation of HIF1α and subsequent vascular blockage. Every T_{O_2} cellular steps, *TumorSim* solves a steady-state diffusion–consumption system on the full grid:

$$\left(\frac{D_{O_2}}{\Delta x^2}, A - \lambda I\right) u = f,$$

where A is the grid Laplacian with periodic boundary conditions, I is the identity, u is the vector of unknown O_2 values, λ is the absorption parameter (`O2.lambda`), and f is the forcing vector. The forcing entry for grid compartment i is:

$$f_i = \begin{cases} -\lambda \cdot O_{2,\mathrm{init}}(i) & \text{if no source or sink,} \\ -\lambda \cdot O_{2,\mathrm{init}}(i) - f_{O_2,\mathrm{VS}} \cdot \mathrm{Blockage}\big(C_{\mathrm{HIF1}\alpha}(i)\big) & \text{if } i \text{ hosts a vascular source,} \\ -\lambda \cdot O_{2,\mathrm{init}}(i) + c_{O_2,\tau} & \text{if } i \text{ hosts a consuming cell of type } \tau. \end{cases}$$

Cancer cells and macrophages consume more oxygen than CTLs and Tregs (§6.2). The linear system is solved iteratively (sparse solver) with relative tolerance 10^{-8} and a 2000-iteration cap; failure to converge is reported as a fatal error. Local O_2 values are then converted from molecules/compartment to mg/L for HIF1α evaluation.

HIF1α from O_2. Relative HIF1α (unitless, 0–1) is computed pointwise as a Hill function inverted in O_2:

$$C_{\mathrm{HIF1}\alpha}(O_2) = 1 - H_{+}^{h}\big(O_2;, 1, b_{O_2}, h_{O_2}\big),$$

so that low oxygen produces high HIF1α.

Vascular blockage. Each vascular source has a blockage value computed from local HIF1α:

$$\mathrm{Blockage}(C_{\mathrm{HIF1}\alpha}) = H_{+}^{h}\big(C_{\mathrm{HIF1}\alpha};, 1, b_{\mathrm{blockage}}, h_{\mathrm{blockage}}\big).$$

Blockage modulates the effective vascular passage in the O_2 forcing vector and influences recruitment via the source state (§7.4, §7.9).

7.3 Movement and chemotaxis

This section describes how cellular movement occurs both with and without chemotaxis.

Motile immune cells (CTLs, Tregs, macrophages) attempt at most one Moore-step per Δt_s, subject to the macrophage movement intervals in §6.8. Cancer cells do not actively migrate.

For a moving cell c, the candidate destination set is $N_8(\mathrm{c.pos})$, which includes c's current compartment (so a cell may probabilistically remain stationary). Chemotaxis activates only when the local field at c.pos lies within $C_{\mathrm{min}} < C < C_{\mathrm{max}}$ (§6.1). For each candidate destination i, *TumorSim* computes a weight

$$w_i = \sum_{m \in M_c} C_m(i),$$

where M_c is the set of chemokines sensed by c's type (CTLs: CCL5, CXCL9; Tregs: CCL5; macrophages: CCL2, CCL5). If $\sum_j w_j > 0$, the destination is drawn from the categorical distribution $P_i = w_i / \sum_j w_j$; otherwise it is sampled uniformly from $N_8(\mathrm{c.pos})$. If the selected destination is occupied, the move fails and c remains at c.pos. Movement is sequential in current list order: subsequent attempts see the updated grid occupancy.

7.4 Recruitment

Here we describe how cells are recruited from vascular sources based on local chemokine concentrations. Each vascular source evaluates recruitment of each recruitable type once per Δt_s. The probability uses the type-specific summed-H_+ form over the chemokines that recruit that type:

$$p_{\text{rec,M1}} = p_{\text{rec,M2}} = b \cdot R_{\text{max},M} \left[H_+(C_{\text{CCL2}}; 1, K_M) + H_+(C_{\text{CCL5}}; 1, K_M) \right],$$

$$p_{\text{rec,CTL}} = b \cdot \frac{R_{\text{max,CTL}}}{2} \left[H_+(C_{\text{CCL5}}; 1, K_{\text{CTL}}) + H_+(C_{\text{CXCL9}}; 1, K_{\text{CTL}}) \right],$$

$$p_{\text{rec,Treg}} = b \cdot R_{\text{max,Treg}}, H_+(C_{\text{CCL5}}; 1, K_{\text{Treg}}).$$

Parameters per §6.10. M1 and M2 use separate XML parameters in the implementation but share the same functional form. Newly recruited cells are appended to a temporary new-cell list and placed during the growth/shrinkage phase. Newly recruited CTLs are entered as active, reflecting prior lymph-node priming. Here, b is vascular blockage (§7.2).

7.5 Proliferation, new-cell placement, and tumor growth/shrinkage

This submodel is the most intricate part of *TumorSim*: it reconciles continuous biological events (proliferation, recruitment, death, degradation) with the one-cell-per-grid compartment constraint while preserving a compact tumor morphology. It runs once per Δt_s during agent computeNextState during the cellular timestep process (§3.2 for pseudocode).

Proliferation timing. Each cellular agent is assigned a proliferating-fraction Bernoulli outcome at creation; proliferating agents become eligible to generate a daughter when `currentTime - birthTime $\geq$ doublingTime`. Representative intervals (from §6):

Cell type	Doubling time
Cancer cell	36–48 h
Active CTL	8 h
Inactive CTL	7.5 d
Treg	5 d
M1 macrophage	36 h
M2 macrophage	48 h

Phases of the growth/shrinkage step.

1. Determine the **tumor boundary**: occupied compartments containing a movable cell with at least one unoccupied Moore-neighbor. The full grid is scanned at the start of each step.
2. Collect removed agents (in the present configuration: degraded cancer cells) into a position-indexed map.
3. Place all new cells in the temporary new-cell list, growing or rearranging as needed.
4. Randomize the order of remaining removed agents.
5. Process each removed agent via a shrinkage cascade.
6. Clear temporary collections.

New-cell placement (growth cascade). Each new cell has a starting position startPos (typically the position of the cell or source event that generated it). An endpoint endPos is chosen by branch:

- *In-tumor new cells*: endPos = the boundary position with minimum squared Euclidean distance from startPos; ties broken randomly. The intuition here is that within a tumor, cells are added adjacent to where they are recruited to or in the position where their parent cell divided, and push a stack of other non-immovable agents toward a nearest tumor boundary.
- *Out-of-tumor new cells*: endPos = the nearest valid position that has a movable agent and has an unoccupied neighbor; ties broken randomly. The intuition here is that newly-recruited or proliferated cells outside of a tumor will be placed adjacent to where they enter the simulation and push a shortest-length stack of cells toward an unoccupied grid compartment.

Starting from startPos, the placement cascade displaces cells along a path toward endPos:

```
displaced ← newCell ; pos ← startPos
loop:
    next ← neighbor of pos closest to endPos, excluding pos and immovable agents
    if next contains a removed agent:
        place displaced at next; remove the agent from the removed map; done
    if next is empty:
        place displaced at next; done
    swap: place displaced at next; displaced ← cell previously at next; pos ← next
    if pos == endPos:
        place displaced at a randomly chosen open neighbor of endPos; done
```

Allowing empty positions as destinations prevents infinite ping-ponging when no all-occupied path exists. The boundary set is updated after the cascade.

Removal (shrinkage cascade). For each remaining removed agent at position endPos, *TumorSim* selects the boundary position startPos with *maximum* squared Euclidean distance from endPos (ties broken randomly). The cell at startPos is pulled inward along a path of nearest-to-endPos neighbors, with each step displacing the cell currently at that position outward, until the cell at endPos has been replaced. The removed agent is then discarded and the boundary set is updated.

Rationale. The cascade preserves three constraints: one-cell-per-compartment occupancy; compact tumor morphology (push toward nearest boundary on growth, pull from farthest on shrinkage); and avoidance of deterministic spatial artifacts (random tie-breaking; randomized removed-agent order). This algorithm is part of the model definition because it shapes how cell addition and removal alter spatial structure.

7.6 CTL, cancer-cell, and PD-1/PD-L1 interactions

This section describes our implementation of CTL activation state and PD-1 status, cancer cell PD-L1 status, and how this impacts cytotoxic activity.
These rules together represent the dynamics of CTL-based killing of cancer influenced by the PD-1/PD-L1 suppression axis. All probabilities are evaluated as independent Bernoulli draws per §2.1.

CTL activation. IFN-γ-dependent activation:

$$p_{\mathrm{act}}(C_{\mathrm{IFN}\gamma}) = H_+(C_{\mathrm{IFN}\gamma};, V_{\mathrm{max,act}}, C_{50,\mathrm{act}}).$$

Contact-mediated activation (CTL adjacent to a cancer cell) occurs with constant probability `probActivationByCancerCell`. M1-mediated activation (CTL adjacent to M1) occurs with constant probability `m1ActivateCTL`.

CTL PD-1 conversion. HIF1α-driven induction, as a proxy for hypoxia:

$$p(\mathrm{PD1}^- \to \mathrm{PD1}^+) = H_+(C_{\mathrm{HIF1}\alpha};, V_{\max}, C_{50}),$$

using the `Core.CTL.vmaxPD1MinusToPD1PlusConversion` / `c50PD1MinusToPD1PlusConversion` parameters. IFN-γ-driven loss:

$$p(\mathrm{PD1}^+ \to \mathrm{PD1}^-) = H_+(C_{\mathrm{IFN}\gamma};, V_{\max}, C_{50}),$$

using the `Core.CTL.vmaxPD1PlusToPD1MinusConversion` / `c50PD1PlusToPD1MinusConversion` parameters.

Cancer-cell PD-L1 induction. HIF1α-driven, as a proxy for hypoxia:

$$p(\mathrm{PDL1}^- \to \mathrm{PDL1}^+) = H_+(C_{\mathrm{HIF1}\alpha};, V_{\max}, C_{50}),$$

using the `Core.CancerCell.vmaxPDL1MinusToPDL1PlusConversion` / `c50PDL1MinusToPDL1PlusConversion` parameters.
Contact-mediated: a CTL induces PD-L1 on an adjacent cancer cell with probability `Core.CTL.probInducePDL1`.

CTL-mediated cancer-cell killing. An active CTL evaluates killing against each adjacent cancer cell using the (PD-1 status × PD-L1 status) probability matrix:

CTL state	Cancer-cell state	Kill probability
Active PD-1⁻	PD-L1⁻	`probActPD1MinusKillPDL1Minus`

CTL state	Cancer-cell state	Kill probability
Active PD-1^{-}	PD-L1^{+}	`probActPD1MinusKillPDL1Plus`
Active PD-1^{+}	PD-L1^{-}	`probActPD1PlusKillPDL1Minus`
Active PD-1^{+}	PD-L1^{+}	`probActPD1PlusKillPDL1Plus`

In the representative parameterization, killing is most effective for PD-1^{-} on PD-L1^{-} and effectively zero for PD-1^{+} on PD-L1^{+}. Killed cancer cells enter the dead state and degrade after `cellDegradedInterval` (§6.5), at which point they enter the removed-agent list for the next growth/shrinkage phase.

PD-L1-mediated CTL deactivation. A PD-L1^{+} cancer cell deactivates an adjacent active PD-1^{+} CTL with probability `Core.CancerCell.probPDL1PlusDeactActPD1PlusCTL`.

7.7 Treg actions

These rules show how we have implemented the recruitment and suppressive activity of Tregs. Tregs migrate by CCL5 chemotaxis (§7.3), are recruited via CCL5 (§7.4), proliferate per their assignment (§7.5), and at each step evaluate adjacent CTLs in N_8: a Treg deactivates an adjacent CTL with probability `Core.Treg.probDeactCTL`. Deactivated CTLs lose cytotoxic competence until reactivated.

7.8 Macrophage actions and polarization

M1 actions. M1 macrophages kill adjacent cancer cells with probability `Core.Mac.m1KillProb`; activate adjacent CTLs with probability `Core.Mac.m1ActivateCTL`; secrete IFN-γ at a constant rate (§7.1); and secrete CXCL9 in response to IFN-γ via H_+ (§7.1).

M2 actions. M2 macrophages deactivate adjacent CTLs with probability `Core.Mac.m2CTLDeactProb`.

Polarization. Both transitions use H_+:

$$p(\mathrm{M1} \to \mathrm{M2}) = H_+\big(C_{\mathrm{HIF1}\alpha};\, , V_{\mathrm{max,M1M2}}, C_{50,\mathrm{M1M2}}\big),$$

$$p(\mathrm{M2} \to \mathrm{M1}) = H_+\big(C_{\mathrm{IFN}\gamma};\, , V_{\mathrm{max,M2M1}}, C_{50,\mathrm{M2M1}}\big).$$

This couples local hypoxia to immunosuppressive polarization and local IFN-γ to anti-tumor repolarization.

7.9 Vascular sources

Vascular sources contribute to the O_2 forcing vector with effective input modulated by blockage (§7.2); recruit immune cells according to §7.4; and update their blockage and (if enabled) source-state transitions at each step.

7.10 In-tumor classification

The rule for defining cells as in-tumor or out-of-tumor is defined in §2.3, and is applied to CTLs, Tregs, and macrophages each step. Cancer cells are unconditionally in-tumor. The classification feeds the in-tumor placement branch of §7.5 and the observation outputs in §4.10.

7.11 Stochastic event resolution

All probabilistic events are independent Bernoulli draws as stated in §2.1. When multiple potential interactions are possible for one agent in one step (e.g., a CTL evaluating activation, PD-1 conversion, killing of multiple neighbors, and proliferation) each is drawn independently in the order specified by the submodel.

7.12 Periodic list shuffling

TumorSim applies list-based partial-asynchronous scheduling as described in the beginning of §3. Briefly, the ordering of actions is partially ordered as cells are grouped into lists based on cell types. Cell ordering within a list is shuffled, but each list is resolved in the order of: cancer → CTL → Treg → macrophage → vascular source.

Every T_{shuffle}, *TumorSim* randomly shuffles the within-type ordering of each agent list (cancer cells, CTLs, Tregs, macrophages, vascular sources). Shuffling changes only the order in which agents are processed; it does not alter positions, states, or grid occupancy.

7.13 Summary of key submodel couplings

Each of the submodels interface to mechanistically produce emergent effects; these effects are part of the foundational design principles for ABMs (see §3 for more details).
Here, we summarize how the submodels described above represent biological observations.

**Interactions of submodels**	**Mechanism**
Cancer ⇔ chemokines ⇔ recruitment and chemotaxis	Constitutive CCL2/CCL5 secretion shapes immune-cell arrival and movement
M1, CTL ⇔ IFN-γ ⇔ activation, polarization	IFN-γ activates CTLs, drives M2→M1, induces CXCL9 in M1
M1 ⇔ CXCL9 ⇔ CTL recruitment/chemotaxis	CXCL9-based signaling amplifies cytotoxic infiltration
Cellular consumption ⇔ O_2 depletion ⇔ HIF1α	Hypoxia emerges where demand exceeds supply
HIF1α ⇔ immunosuppression	PD-1, PD-L1, M1→M2, CTL IFN-γ inhibition
HIF1α ⇔ vascular blockage ⇔ reduced O_2/recruitment	Hypoxia degrades vascular function in the short term
CTL, M1 ⇔ cancer-cell death	Direct cytotoxicity
Treg, M2, PD-L1$^+$ cancer cells ⇔ CTL deactivation	Three parallel suppression pathways
Proliferation, degradation, placement cascades ⇔ Tumor morphology	Growth/shrinkage submodel shapes tumor structure

Together these couplings generate the tumor-level outcomes characterized in main text Section 3.
