# Supplementary material for: A Comparison of Mechanisms Driving Lesion Outcomes During Lung Tumor and Tuberculosis Granuloma Formation
Source: Bull Math Biol. 2026 Oct 6;88(11):197. doi: 10.1007/s11538-026-01763-8 (PMC13642502; doi:10.1007/s11538-026-01763-8)
Supplement: Supplementary file 2 [file 11538_2026_1763_MOESM2_ESM.pdf]

Supplementary Material: This document contains additional details for our model of oxygen in *TumorSim* (Section S1), multiple visualizations of lesion outcomes in both *TumorSim* and *GranSim* (Section S2), and scatterplots of *TumorSim* outcomes (Section S3).

S1 - Modeling steady-state oxygen distributions in *TumorSim*

The levels of O_2 on the simulation grid depends on O_2 sources (e.g., vascular sources, breathing) and O_2 sinks (cells that consume O_2 like macrophages, cancer cells and T cells). Since the change in spatial distribution of sources and sinks is slow compared to O_2 diffusion, we calculate steady-state O_2 concentrations on the grid based on the location of sources and sinks and update oxygen concentrations every 24 hours.

To calculate steady state O_2 levels on an n-by-n grid, we solve the heat equation, such that:

$$\left(\frac{D_{O2}}{\Delta x^2}A - \lambda I\right)u = f \qquad \text{(Eq. S1)}$$

where D_{O2} is diffusion coefficient of O_2 (diffusivityO2 in *TumorSim*, 3.08e-5 cm^2/s), Δx is the length of one grid compartment (20 μm), A is an n^2-by- n^2 Laplacian matrix, f is a n^2-by- 1 forcing vector for the O_2 contribution from sources and sinks (molecules/sec), λ is the absorption rate constant and u is a n^2-by- 1 vector of O_2 concentrations.

S1.1 - Forming the Laplacian matrix A

We form the matrix A based on the boundary condition we choose to use. It is possible to divide A into n^2 B_{nxn} submatrices for simplicity, once we have chosen a boundary condition. In[1], the Dirichlet boundary condition was used under the assumption that boundaries act as sources as the boundaries always have a constant level of O_2 due to breathing. Here, the diagonal Bs (i.e., C) have -4s at the diagonal and 1s off-diagonal, given by

$$C_{n\times n} = \begin{bmatrix} -4 & 1 & 0 & & & & \\ 1 & -4 & 1 & \cdots & & 0 & \\ 0 & 1 & -4 & & & & \\ & \vdots & & \ddots & & \vdots & \\ & & & & -4 & 1 & 0 \\ & 0 & & \cdots & 1 & -4 & 1 \\ & & & & 0 & 1 & -4 \end{bmatrix}$$

and

$$A_{n^2\times n^2} = \begin{bmatrix} C & I & 0 & & & & \\ I & C & I & \cdots & & 0 & \\ 0 & I & C & & & & \\ & \vdots & & \ddots & & \vdots & \\ & & & & C & I & 0 \\ & 0 & & \cdots & I & C & I \\ & & & & 0 & I & C \end{bmatrix},$$

where I is n-by-n identity matrix. Dirichlet boundary condition was creating higher O_2 concentrations in the center even when sources are distributed randomly in the grid and no sources present (Fig. S1).

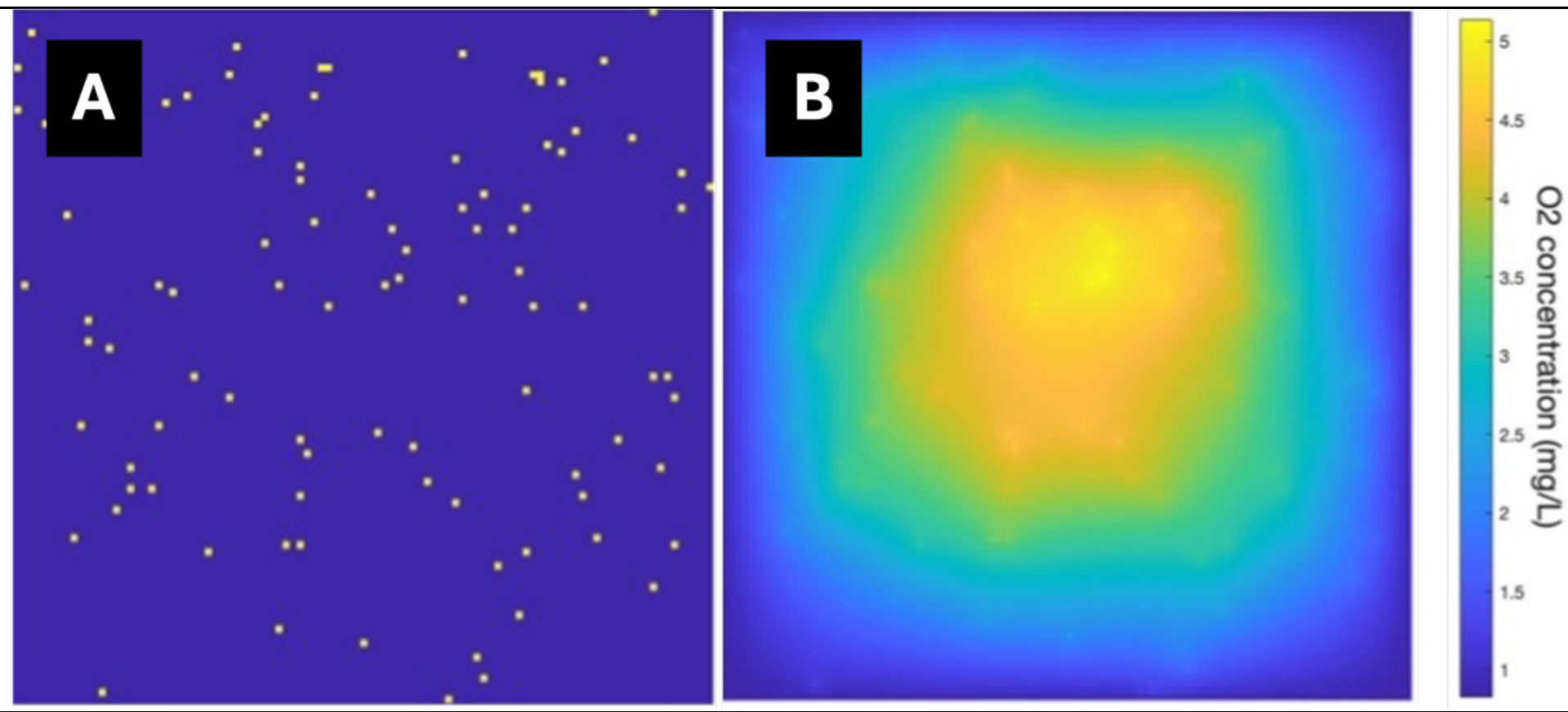

Fig. S1 Oxygen distribution in virtual tissue. (A) Locations of vascular sources. (B) O2 concentrations after steady state calculations have been performed using Dirichlet boundary conditions.

For *TumorSim*, we expect to have a constant O_2 levels throughout the grid when sources are distributed equally on the grid, therefore. For this reason, we instead use the periodic boundary condition. This changes the structure of C to have -4s at the diagonal,1s off-diagonal and also 1s at (1,n) and at (n,1):

$$C_{n\times n} = \begin{bmatrix} -4 & 1 & 0 & & 0 & 0 & 1 \\ 1 & -4 & 1 & \cdots & 0 & 0 & 0 \\ 0 & 1 & -4 & & I & 0 & 0 \\ & \vdots & & \ddots & & \vdots & \\ 0 & 0 & 0 & & -4 & 1 & 0 \\ 0 & 0 & 0 & \cdots & 1 & -4 & 1 \\ 1 & 0 & 0 & & 0 & 1 & -4 \end{bmatrix}$$

and

$$A_{n^2\times n^2} = \begin{bmatrix} C & I & 0 & & 0 & 0 & I \\ I & C & I & \cdots & 0 & 0 & 0 \\ 0 & I & C & & 0 & 0 & 0 \\ & \vdots & & \ddots & & \vdots & \\ 0 & 0 & 0 & & C & I & 0 \\ 0 & 0 & 0 & \cdots & I & C & I \\ I & 0 & 0 & & 0 & I & C \end{bmatrix}.$$

S1.2 - Rationale for λ

To reach steady state, we require equal source and sink terms, which is not true *a priori* in simulations. Therefore, we assume that the excess O_2 will be absorbed by the epithelial cells (i.e. each grid compartment) proportional to the amount of O_2 they have. We address this by introducing the absorption rate constant λ.

We choose $\lambda = 0.3$ which gives us physiological ranges of O_2 pressure. When we simulate a 100x100 tissue grid with 100 sources, we find a median O_2 level of 64 mmHg, 95th percentile of 111 mmHg and 5th percentile of 46 mmHg. We convert molecules to mmHg by multiplying molecules by 1.2083, assuming body temperature (PV = nRT). We know that O_2 partial pressure in alveolar sacs is 104mmHg, in oxygenated blood is 100mmHg, in epithelial tissue is 40 mmHg.

S1.3 - Formulation of the forcing vector *f*

The vector f_{n2x1} represents the O_2 contribution of each source and sink and the resting amount:

$$f_i = \begin{cases} -\lambda \cdot O2_{grid} & \text{if the grid } i \text{ does not have any sources or sinks} \\ -\lambda \cdot O2_{grid} - fO2_{VS} \cdot Blockage(C_{HIF1\alpha}) & \text{if grid } i \text{ has a vascular source} \\ -\lambda \cdot O2_{grid} + cO2_j & \text{if grid } i \text{ contains a cell that contains O2 and} \\ & j \text{ contains Tregs, Tcyts, M1 or M2 Macrophages} \end{cases}, \quad \text{(Eq. S2)}$$

Here, $i = (x-1) * dim + y$ where dim is the dimension of the grid and x and y are the coordinates on the grid.

S1.3.1: O_2 Boundary Value calculation

The variable $o2BoundaryValue$ represents the number of O_2 molecules diffusing from the inhaled air in the lungs to the lung tissue per second. It is calculated as follows[1]:

1. The average lung contains 1.2 liters of air per breathing, and normal air contains 21% oxygen, so 252 mL of O_2 are in the residual volume of the human lung. Of this amount 25% is transferred to tissue, so a volume of 63 ml O_2 is transferred to tissue per 1200 ml of air.
2. The lung surface is 130m^2, so 63 ml/130m^2 = 0.4846 ml/m^2 = 1.93385e-10 ml/grid as 1 grid is 4e-10m^2.
3. 1 mole of gas at body temperature and 1 atm pressure is 25.4 liters from the ideal gas law. Find number of O2 molecules per grid: 1.93385e-10 (ml/grid) / 25400 (ml/mole) * 6.022e23 (molecules/mole) = 4.5958e+09 molecules
4. This number is per breath, which we assume is per 5 seconds for humans. So, the number of O2 molecules at rest per second is 4.5958e+09/5 = **<u>9.2e+08 molecules /sec</u>**.

S1.3.2 Calculation of the effect of vascular sources on O_2

The variable $sourceO2Effect$ represents the number of O_2 molecules coming through vascular sources per second. We calculate this effect as follows:

1. Pulmonary blood volume is 322 ml/m^2/3.5s[1].
2. In *TumorSim*, one grid is 4e-10m^2: 322/3.5*4e-10 = 3.68e-8 ml/s of blood from one vascular source
3. 1/5 of blood is O_2, yielding 3.68e-8 / 5 = 7.36e-9 ml/s O_2 per vascular source
4. 1 mole of gas at body temperature and 1 atm pressure is 25.4 liters from the ideal gas law. We compute the number of O_2 molecules per vascular source as follows:
   7.36e-9 (ml/s)/25400 (ml/mole) * 6.022e23 (molecules/mole)
   = **<u>1.7e11 molecules/sec</u>**.

Table S1: Parameter values for O_2 supply from vascular sources.

**Parameter**	**Value (molecules/sec)**
o2BoundaryValue	9.2e8
sourceO2Effect	1.7e11

S1.4 - Conversion to mg/L per *TumorSim* timestep

Once we solve Eq.S1 for u, we obtain O_2 values as molecules for each grid compartment. Since TumorSim uses mg/L, we convert u to mg/L as:

u(molecules/grid)*(1mole/6.022e23molecules)*(32000mg/1mole)*(1grid/8e-12L) = u(molecules/grid)*6.6e-9((grid *mg)/(molecules*L)) = u(mg/L)

S2 - Capturing multiple lesion outcomes in *TumorSim* and *GranSim*

S2.1 - Visualization of individual tumor and granuloma agents in representative simulations

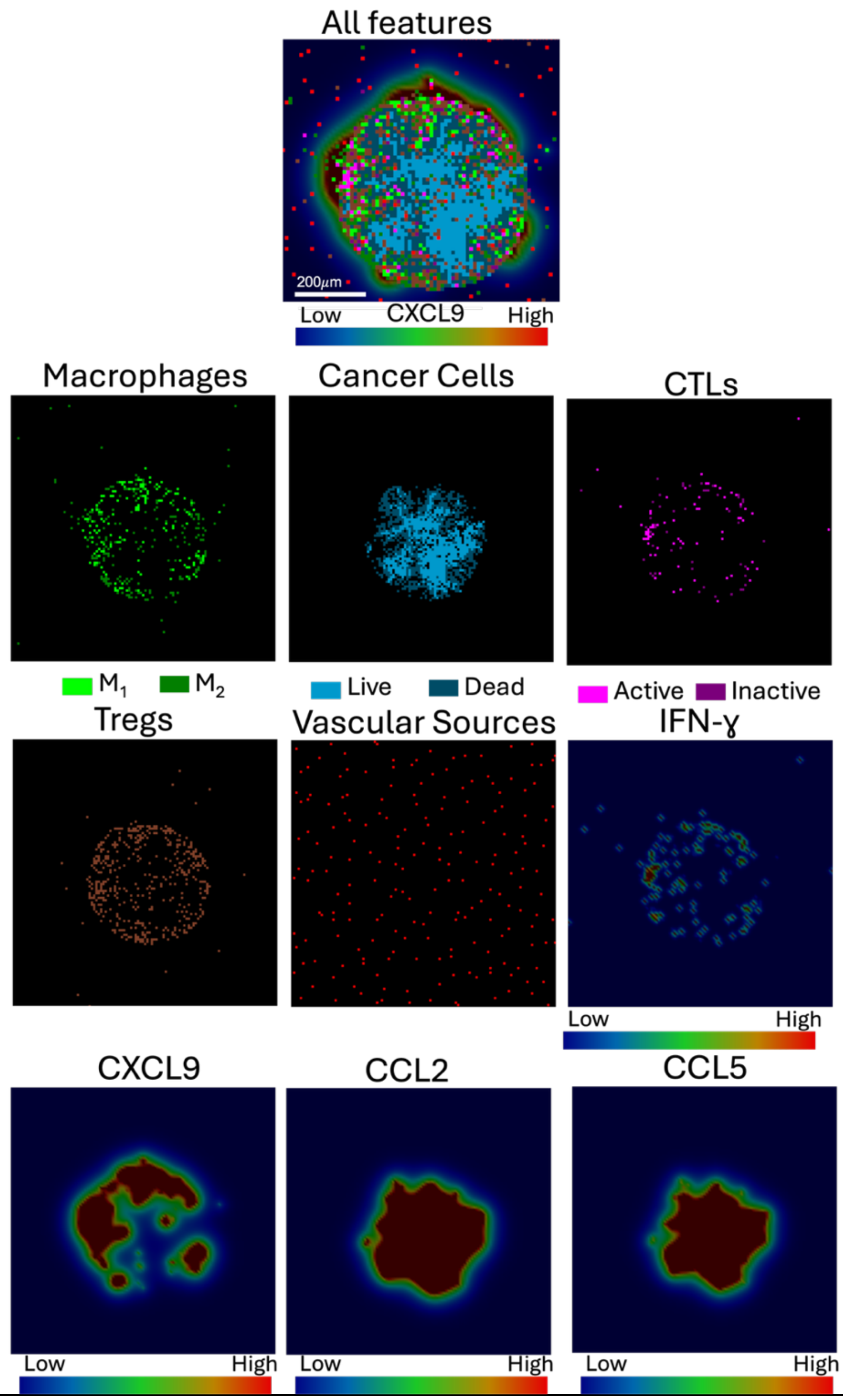

Fig. S2 Selected components of a virtual SCLC tumor. All panels above show the spatial distribution of agents throughout the virtual SCLC tumor shown in Fig. 2A of the main text, which we show here at 14 days after the initiation of the escape phase. CTLs and macrophages are denser in regions where CXCL9 are higher. The large number of Tregs in this tumor are likely responsible for the large number of inactive CTLs.

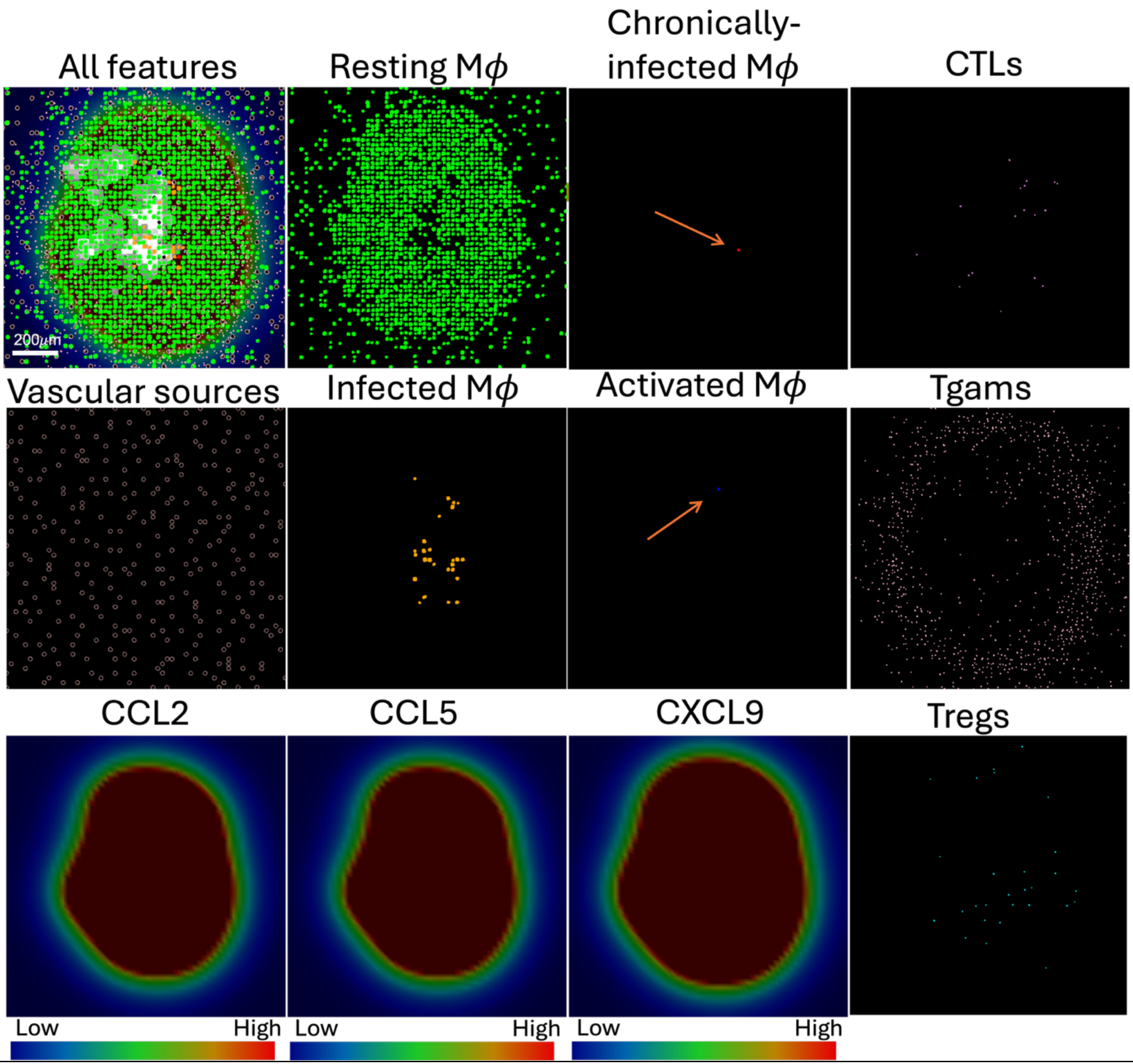

Fig. S3 Selected components of a virtual TB granuloma. All panels above show the spatial distribution of agents throughout the virtual granuloma shown in Fig. 2B of the main text, which we show here at 300 days post-infection. Despite the large number of resting macrophages, only a single macrophage has been activated at this timestep, which is likely preventing complete sterilization. Infected macrophages are localized near the center of the granuloma. Caseum accumulates where many cells have died.

S2.2 - Temporal trajectories of all virtual tumors and granulomas

In Fig. 2 of the main text, we display summary statistics of the 1,500 virtual tumors and 1,500 virtual granulomas we simulated for this study. Fig. S4 shows the individual trajectories used to compute those summary statistics.

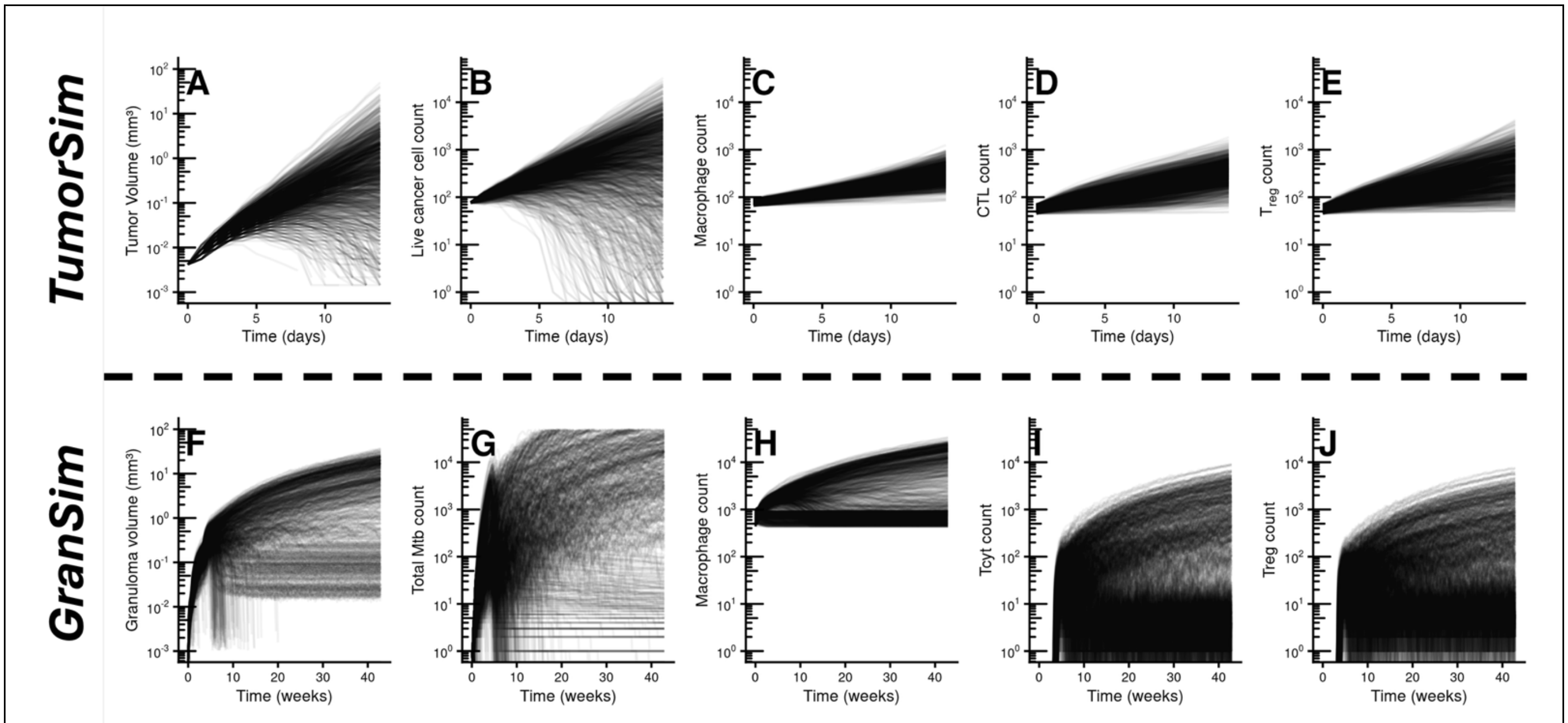

Fig. S4 Temporal trajectories of *TumorSim* and *GranSim* outcomes. Each panel shows the time-course trajectories for multiple temporal outcomes over time for both 1,500 *TumorSim* virtual SCLC tumors (A-E) and 1,500 *GranSim* virtual TB granulomas (F-J). Outcomes include lesion volume, Mtb/Cancer cell counts, and leukocyte counts *GranSim* simulations are run for 300 days to capture both early- and late-state dynamics. (A-B) A small number of tumors spontaneously regress, while many grow exponentially. (F-G) Most granulomas sterilize and/or remain small, while a small number grow slowly over the 300 days of simulation. (G) Note that in many virtual granulomas, the number of Mtb becomes nearly-fixed as a result of non-replicating Mtb being sequestered within caseum. (A-J) To plot outcomes on a $\log_{10}$ scale, we have added 1 to all cell counts and $10^{-3}mm^3$ to lesion volumes. Each trajectory was generated as black and with an alpha-value of 0.05 for visibility.

S2.3 - Visualized lesion development for several representative SCLC and TB lesions

In section 2.4 of the main text, we describe three qualitative outcome states of both SCLC tumors and TB granulomas.
Virtual SCLC tumors from *TumorSim* can be:

- Progressing, characterized by an increasing tumor volume over time;
- Dormant, characterized by a maintained tumor volume over time; or
- Regressing, characterized by a decreasing tumor volume over time.

Fig. S5 shows examples of each type of virtual tumor as simulated in *TumorSim*.

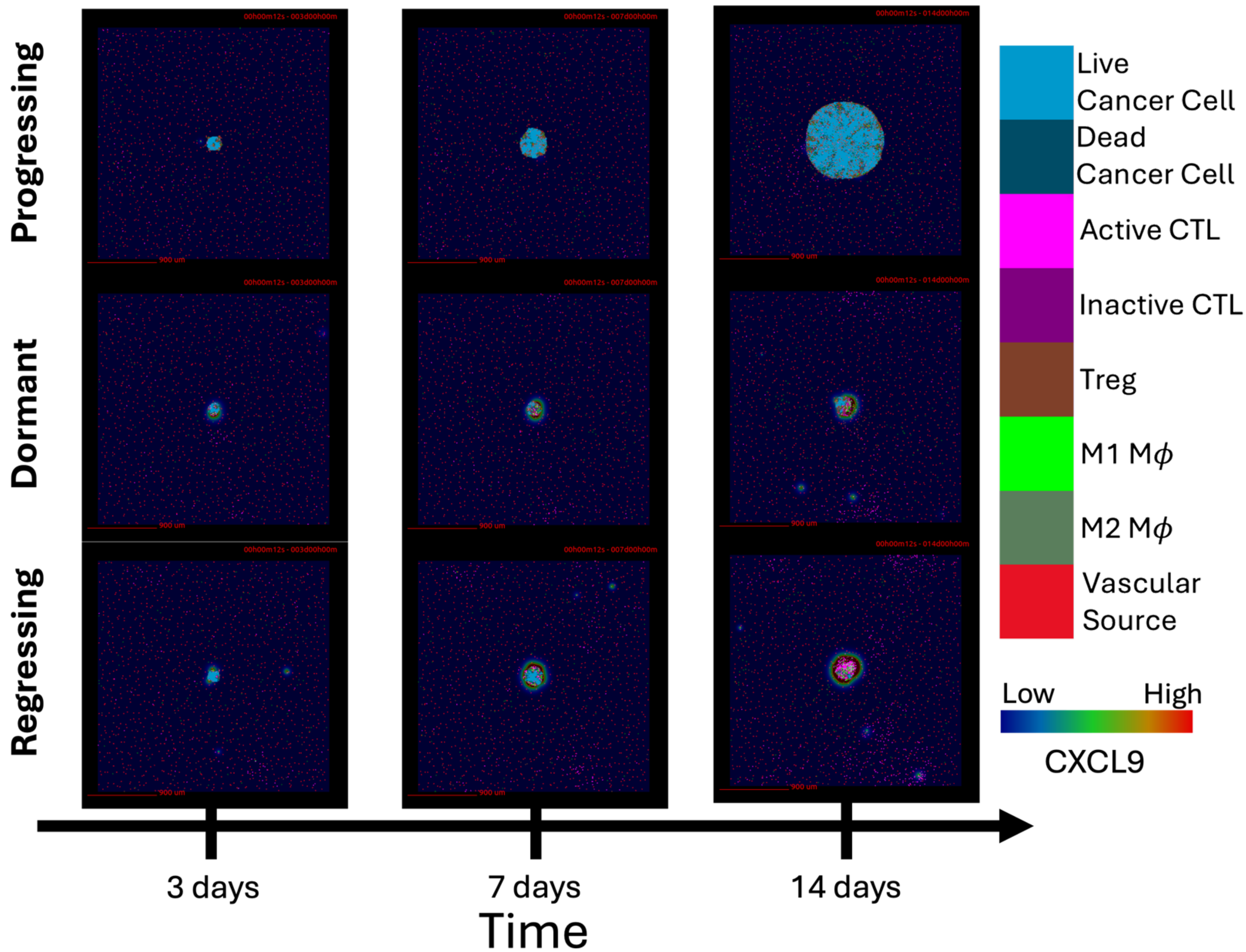

Fig. S5 Spectrum of virtual tumor outcomes in *TumorSim*. Each row shows the development of one virtual tumor from each of the qualitative tumor outcomes over two weeks. (Top row) Progressing tumor with a characteristic lack of Tcyts and M1 macrophages, sometimes called "immune cold". (Middle row) Dormant tumor that appeared with a moderate immune response sufficiently effective to keep the total number of cancer cells within an order of magnitude of its initial state for two weeks. (Bottom row) Regressing tumor characterized by an overwhelming cytotoxic immune response; this happens as the tumor is small and would likely not be detectable.

Virtual TB granulomas from *GranSim* can be:

- Disseminating, characterized by an increasing Mtb count throughout the simulation;
- Controlling, characterized by a persistent small number of Mtb over time; or
- Sterilizing, characterized by a complete lack of Mtb.

Fig. S6 shows examples of each type of virtual granuloma as simulated in *GranSim*.

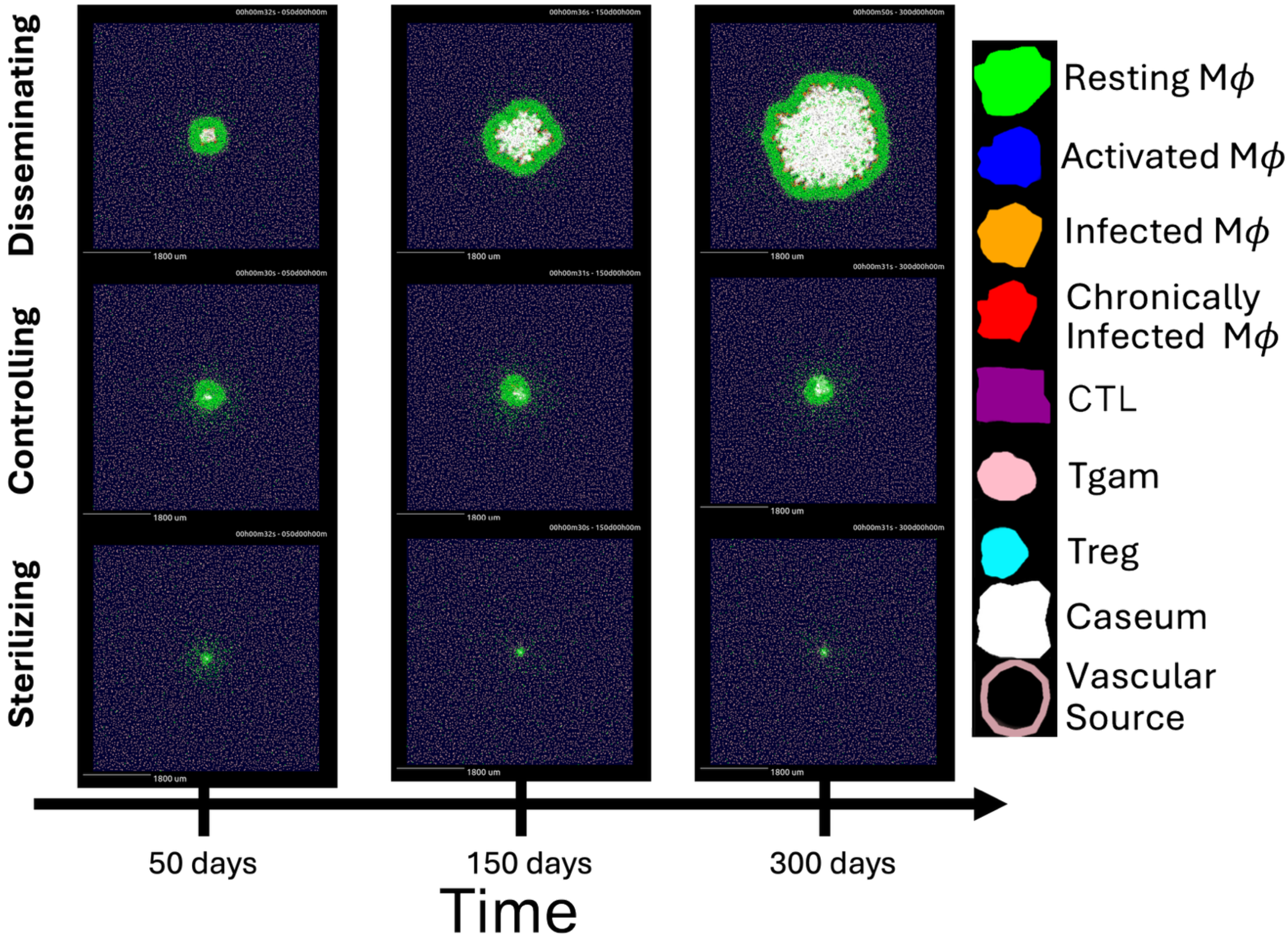

Fig. S6 Spectrum of virtual granuloma outcomes in *GranSim*. Each row shows the development of one virtual granuloma from each of the qualitative granuloma outcomes over 300 days. (Top row) Disseminating granuloma with a characteristic excess of caseum and continued growth. (Middle row) Controlling granuloma with an immune response sufficient to stabilize the total number of Mtb, with slight caseation after 300 days. (Bottom row) Sterilizing granuloma.

S3 - Scatterplots of selected *TumorSim* outputs by parameters

The following scatterplots (Figs. S7-S11) show significant parameter values (horizontal axes) plotted against the five Tumor outcomes shown in Fig. 2 (C-G). As this is the first iteration of *TumorSim*, we present these scatterplots to show monotonicity between parameter values and model outcomes to satisfy the assumptions of the Partial Rank Correlation Coefficients method[2].

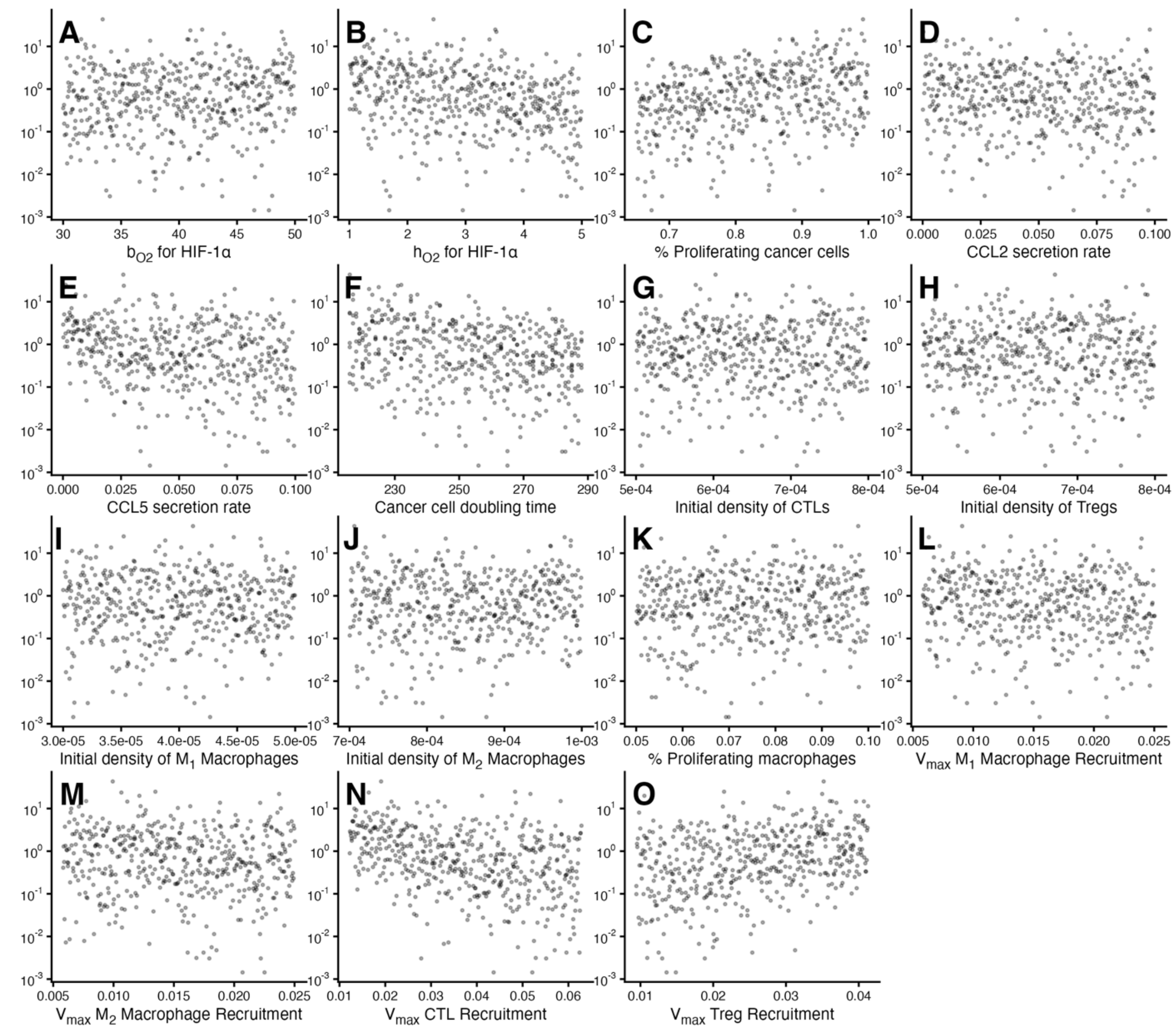

Fig. S7 Scatter plots of virtual tumor volume by PRCC-significant parameter values. For each panel, the y-axis is simulated tumor volume in mm^3. We add 10^{-3}, which was below the minimum tumor volume, to plot on a logarithmic scale. Each point shows the average *TumorSim* value across three replicates simulations run with the same values for all model parameters. Values shown are measured at the simulation end-point, which is 14 days after the initiation of the escape phase.

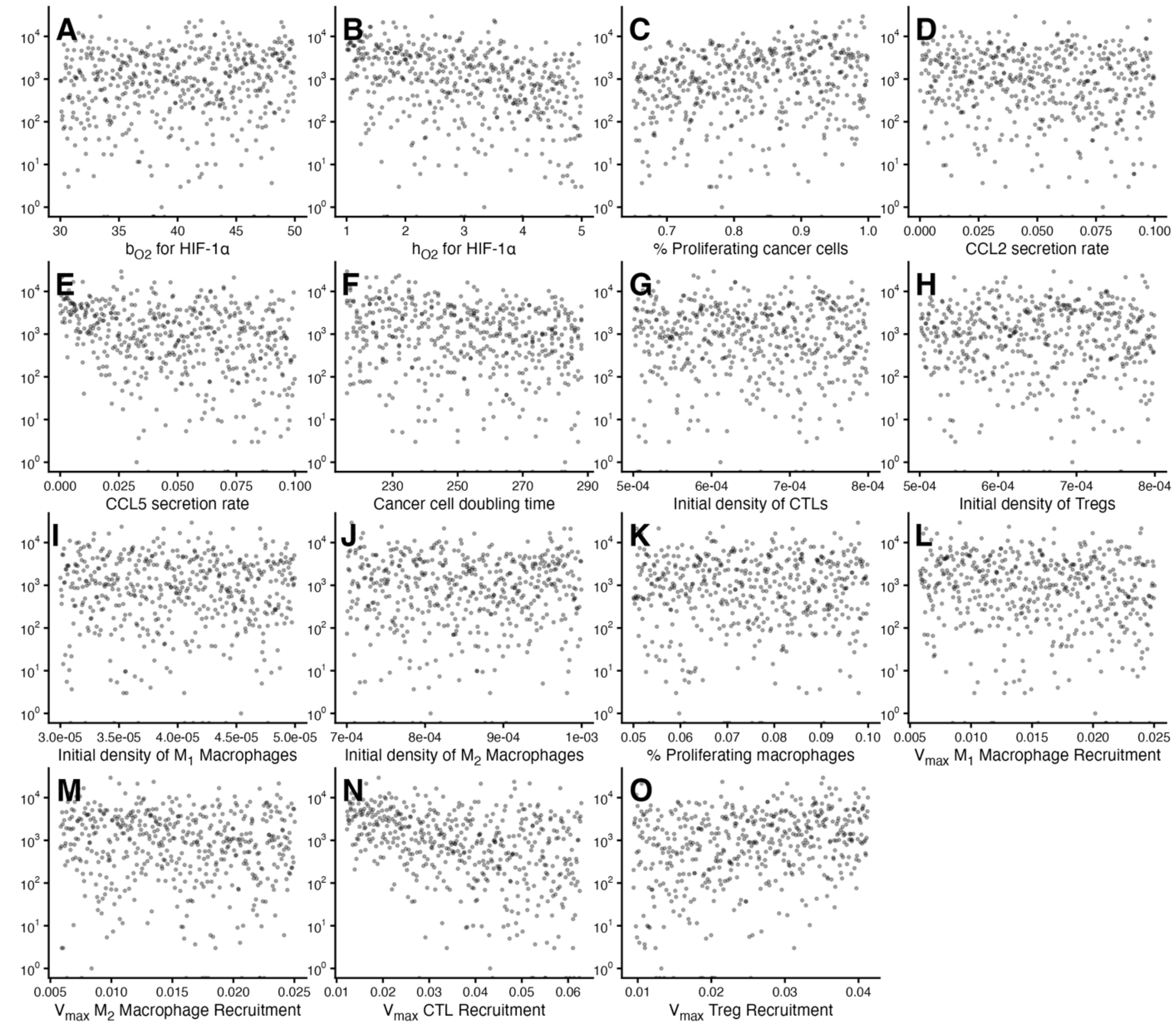

Fig. S8 Scatter plots of virtual cancer cell count by PRCC-significant parameter values. For each panel, the y-axis is 1+(#cancer cells). We add 1 to plot on a logarithmic scale. Each point shows the average *TumorSim* value across three replicates simulations run with the same values for all model parameters. Values shown are measured at the simulation end-point, which is 14 days after the initiation of the escape phase.

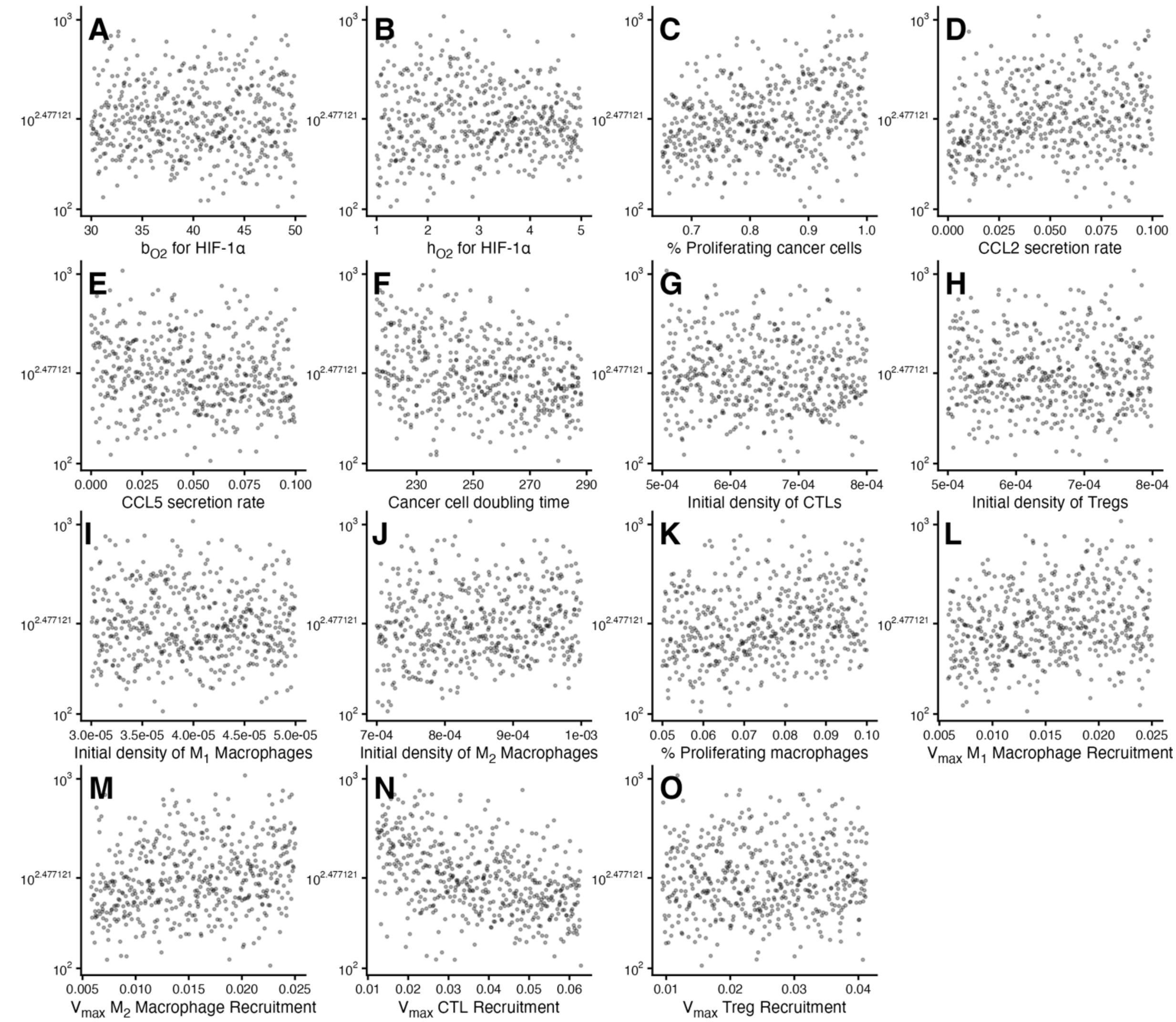

Fig. S9 Scatter plots of virtual macrophage count by PRCC-significant parameter values. For each panel, the y-axis is 1+(#macrophages). We add 1 to plot on a logarithmic scale. Each point shows the average *TumorSim* value across three replicates simulations run with the same values for all model parameters. Values shown are measured at the simulation end-point, which is 14 days after the initiation of the escape phase.

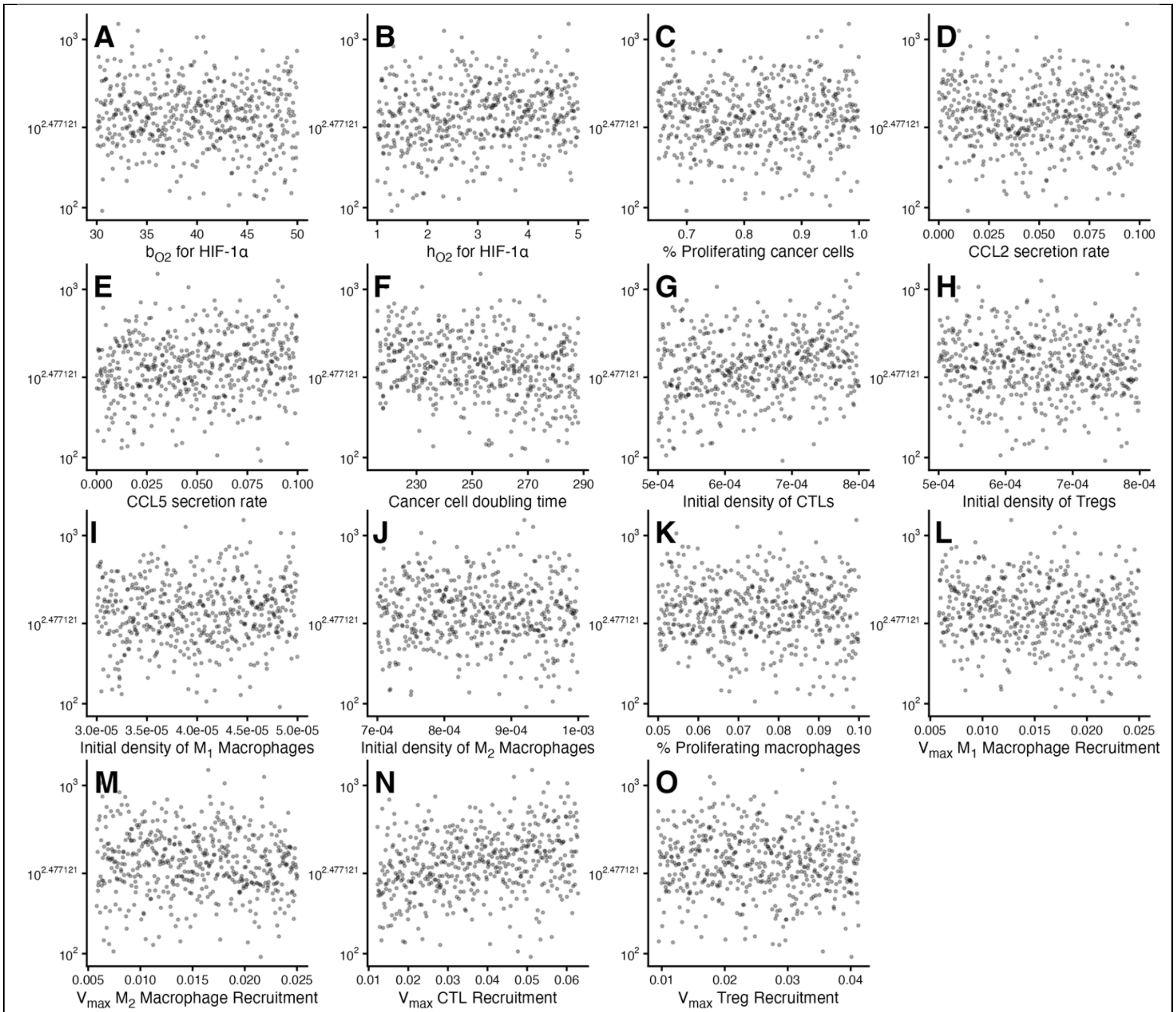

Fig. S10 Scatter plots of virtual CTL count by PRCC-significant parameter values. For each panel, the y-axis is 1+(#CTLs). We add 1 to plot on a logarithmic scale. Each point shows the average *TumorSim* value across three replicates simulations run with the same values for all model parameters. Values shown are measured at the simulation end-point, which is 14 days after the initiation of the escape phase.

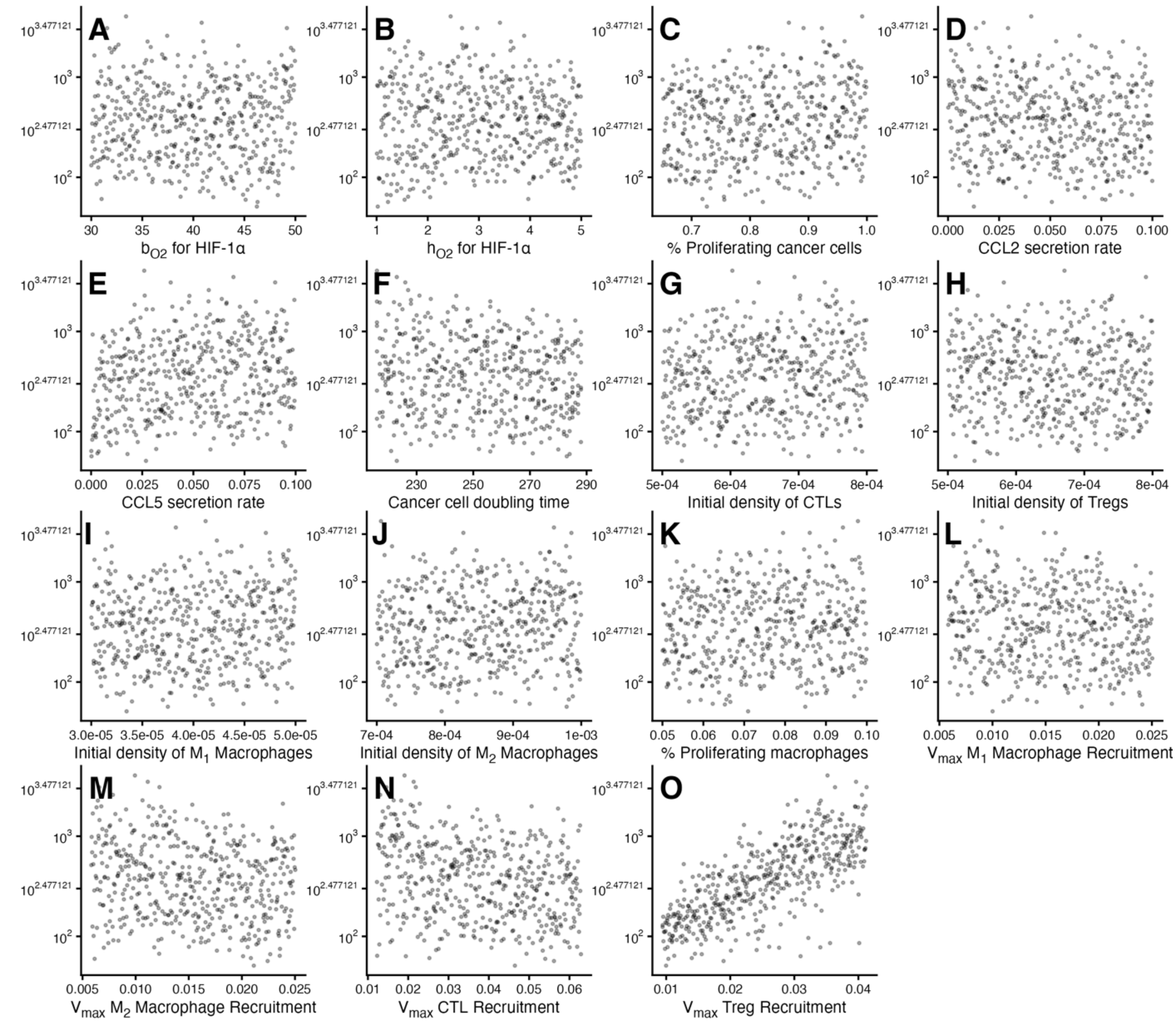

Fig. S11 Scatter plots of virtual T_{reg} count by PRCC-significant parameter values. For each panel, the y-axis is 1+(#Tregs). We add 1 to plot on a logarithmic scale. Each point shows the average *TumorSim* value across three replicates simulations run with the same values for all model parameters. Values shown are measured at the simulation end-point, which is 14 days after the initiation of the escape phase.

References

1 Sershen, C. L., Plimpton, S. J. & May, E. E. Oxygen Modulates the Effectiveness of Granuloma Mediated Host Response to Mycobacterium tuberculosis: A Multiscale Computational Biology Approach. *Frontiers in cellular and infection microbiology* **6**, 6 (2016). https://doi.org/10.3389/fcimb.2016.00006

2 Marino, S., Hogue, I. B., Ray, C. J. & Kirschner, D. E. A methodology for performing global uncertainty and sensitivity analysis in systems biology. *J Theor Biol* **254**, 178-196 (2008). https://doi.org/10.1016/j.jtbi.2008.04.011
